# LncRNA SNHG17 Contributes to Proliferation, Migration, and Poor Prognosis of Hepatocellular Carcinoma

**DOI:** 10.1155/2021/9990338

**Published:** 2021-09-14

**Authors:** Yue Luo, Junhao Lin, Jiakang Zhang, Zhenghui Song, Dayong Zheng, Fengsheng Chen, Xuefen Zhuang, Aimin Li, Xinhui Liu

**Affiliations:** ^1^Integrated Hospital of Traditional Chinese Medicine, Southern Medical University, Guangzhou 510315, China; ^2^Cancer Center, Southern Medical University, Guangzhou 510315, China

## Abstract

Long noncoding RNAs (lncRNAs) have been substantially reported to have critical roles in regulating tumorigenesis in recent years. However, the expression pattern and biological function of SNHG17 in hepatocellular carcinoma (HCC) remain unclear. Bioinformatics analysis and qRT-PCR were performed to detect the expression pattern of SNHG17 in HCC tissues, adjacent nontumorous tissues, and cell lines. The effect of SNHG17 on proliferation, migration, and apoptosis of HCC was investigated by knockdown and overexpressing SNHG17 in HCC cell lines. RNA sequencing was utilized to explore the underlying mechanism. Utilizing publicly available TCGA-LIHC, GSE102079 HCC datasets, and qRT-PCR, we found SNHG17 was significantly upregulated in HCC tissues and cell lines and was notably associated with larger tumor size, poorly differentiation, presence of vascular invasion, and advanced TNM stage. Furthermore, gain- and loss-of-function studies demonstrated that SNHG17 promoted cell proliferation and migration and inhibited apoptosis of HCC. By employing RNA sequencing, we found knockdown of SNHG17 caused 1037 differentially expressed genes, highly enriched in several pathways, including metabolic, PI3K-Akt, cell adhesion, regulation of cell proliferation, and apoptotic pathway; among them, 92 were overlapped with SNHG17-related genes in the TCGA-LIHC dataset. Furthermore, ERH, TBCA, TDO2, and PDK4 were successfully validated and found significantly dysregulated in HCC tissues. Moreover, HCC patients with higher SNHG17 expression had a relatively poor overall survival and disease-free survival, and ERH and PDK4 also played a marked role in the prognosis of HCC. Broadly, our findings illustrate that SNHG17 acts as a noncoding oncogene in HCC progression, suggesting its potential value as a novel target for HCC therapy.

## 1. Introduction

Hepatocellular carcinoma (HCC) is one of the major causes of cancer-related death worldwide [1]. It is particularly prevalent in China, sub-Saharan Africa, and southeast and eastern parts of Asia, over 50% of which were found in China [2]. Despite the diagnostic and therapeutic strategies of HCC that have been greatly improved in the last decade, the prognosis of HCC patients remains very poor. Investigating the molecular mechanism underlying the development of HCC and identifying effective biomarkers and therapeutic targets for HCC are extremely urgent [3].

Recently, with the development of high-throughput transcriptome analysis, long noncoding RNAs (lncRNAs), which are a subclass of functional ncRNAs without protein encoding abilities and consist of over 200 nucleotides, have been confirmed in a number of studies to be a vital player in human diseases including cancer [4–7]. Aberrant expression of lncRNAs exerts a suppressive or oncogenic role in numerous cancers including lung cancer, breast cancer, gastric cancer, and HCC [8–10]. Growing evidence reveals that lncRNAs plays an extensive function in the occurrence and progression of HCC. For example, HOTAIR, MEG3, and Lnc-SchLAH were found to be involved in HCC proliferation, autophagy, and metastasis [11–13].

Small nucleolar RNA host gene 17 (SNHG17), as a 1186-nt lncRNA and located on human chromosome 20, has been reported as an oncogenic gene in colorectal cancer, gastric cancer, non-small-cell lung cancer, breast cancer, melanoma, and glioma through regulating cellular proliferation, apoptosis, and metastasis [14–19]. However, the expression pattern, biological function, and underlying molecular mechanism of SNHG17 in HCC remain unclear.

Here, we uncovered SNHG17 was significantly upregulated in HCC tissues and cell lines and was significantly correlated with poor clinical characteristics and prognosis of HCC patients. Moreover, SNHG17 promoted cell proliferation and migration and inhibited apoptosis of HCC in vitro by gain- and loss-of-function of SNHG17 study. By RNA sequencing, 599 upregulated and 438 downregulated genes caused by knockdown of SNHG17 in HCC were found and were highly enriched in various pathways, such as metabolic pathways, PI3K-Akt, cell adhesion, regulation of cell proliferation, and positive regulation of the apoptotic signaling pathway; among them, 92 were overlapped with SNHG17-related genes in the TCGA-LIHC dataset. Also, ERH, TDO2, TBCA, and PDK4 were further successfully validated and found significantly dysregulated in HCC tissues. Moreover, ERH and PDK4 also play an important role in the prognosis of HCC. Overall, our data showed the vital roles of SNHG17 in HCC progression, indicating its potential value as a therapeutic target for HCC.

## 2. Methods

### 2.1. TCGA and GEO Dataset Analyses

The gene expression profile and clinical characteristics for HCC patients were downloaded from the TCGA database (https://www.cancer.gov/about-nci/organization/ccg/research/structural-genomics/tcga, TCGA-LIHC). The dataset includes 374 tumors and 50 adjacent samples. The GSE102079 dataset containing 152 tumorous and 91 adjacent liver tissues from HCC patients was downloaded from GEO (https://www.ncbi.nlm.nih.gov/geo). R software and its packages were used to process all these data. The Kaplan–Meier plot was determined online for survival analysis to show the correlation between gene expression and overall survival (OS) or disease-free survival (DFS) of HCC patients (https://gepia.cancer-pku.cn/index.html). Univariate and multivariate Cox regression analyses were employed to explore the association between gene expression and overall survival.

### 2.2. Tissue Samples and Ethics Statement

cDNA of 28 paired HCC tissues and matched adjacent nontumorous tissues were purchased from Outdo (Shanghai, China). Tumor tissue was the experimental group, and adjacent tissues were the control group. The study was approved by the Ethical Review Board for Research of TCM-Integrated Cancer Center of Southern Medical University.

### 2.3. Cell Lines and Cell Culture

The normal hepatic epithelial cell line (LO2) and human HCC cell line (SMMC-7721) were cultured in RPMI 1640 medium (Gibco, USA) containing 10% fetal bovine serum (FBS, Gibco, USA), 100 units/ml penicillin and streptomycin at 37°C, and 5% CO_2_. The other HCC cell lines, including HuH-7, Hep3B, HepG2, and PLC/PRF/5, were cultured in DMEM medium containing FBS, penicillin, and streptomycin as mentioned above. All cells were acquired from Zhong Qiao Xin Zhou Biotechnology (Shanghai, China) and cultured in cell culture dishes (Jet Bio-Filtration, Guangzhou, China).

### 2.4. RNA Interference and Plasmid Transfection

The specific siRNA of SNHG17 was designed and offered by RiboBio (Guangzhou, China), and overexpressed SNHG17 was produced with pcDNA3.1 vector (GENECHEM, Shanghai, China). The siRNA sequences were si-SNHG17-1: CGGATCCACTGTTCAATCT; si-SNHG17-2: GCCTGGAATGACTTTAATA. Lipofectamine 3000 transfection reagent (Invitrogen, USA) was used for transient transfection according to the manufacturer's instruction. siRNAs were transfected into Hep3B and PLC/PRF/5, while pcDNA3.1_SNHG17 plasmid was transfected into HuH-7 and SMMC-7721.

### 2.5. RNA Isolation and qRT-PCR

Total RNAs were extracted from cells by using the total RNA isolation kit (Foregene, Chengdu, China) and then reversely transcripted into cDNA with the PrimeScript RT reagent kit (TaKaRa, Japan). qRT-PCR analyses were carried out using SYBR Premix Ex Taq II (TaKaRa, Japan) on LightCycler 480 II (Roche, USA), following the manufacturer's instructions. Expressions of target RNAs were normalized to *β*-actin with the 2^−△△Ct^ method. The specific primers were listed as follows: SNHG17 : 5'-AGAGAATGGAGAGTGAGGCTACC-3' (forward) and 5'-CCAGGCATGGACAGAGGGAT-3' (reverse); ADAM9: 5'-TCACGCAGTTACTCGCTTCC-3' (forward) and 5'-AGGAAGCTACTAGGAGACACAA-3' (reverse); ERH: 5'-AAGAGAGTTTGGCGCGATGT-3' (forward) and 5'-AAGTTCTGCCTTCTGGCCTC-3' (reverse); GLI1: 5'-GGCTATTCTGGATGAGCCCC-3' (forward) and 5'-CATCTTGTGCATGGGACTGC-3' (reverse); PDK4: 5'-CAGACAGGAAACCCAAGCCA-3' (forward) and 5'-GACGAGAAATTGGCAAGCCG-3' (reverse); SIRT4: 5'-ACTGTGGGGTGTGAAGTGTC-3' (forward) and 5'-GGCCAGCCTACGAAGTTTCT-3' (reverse); TBCA: 5'-CAGGTTGGAAGCCGCATATT-3' (forward) and 5'-AGCGGTATAAAGGGCAAGTGA-3' (reverse); TDO2: 5'-GAGACGATGACAGCCTTGGA-3' (forward) and 5'-TGCAAACTCTGGAAGCCTGA-3' (reverse); and *β*-actin: 5'-TGGCACCCAGCACAATGAA-3' (forward) and 5'-CTAAGTCATAGTCCGCCTAGAAGCA-3'(reverse).

### 2.6. Cell Proliferation Assay

Cell proliferation assay was performed using the Cell Counting Kit 8 (Dojindo, Japan). After transfection was accomplished, cells were harvested and suspended into 96-well plates with 5000 cells per well. Cell viability in different groups was monitored by measuring the spectrophotometric absorbance of cells at 450 nm wavelength every 24 hours according to the manufacturer's instructions.

### 2.7. Cell Apoptosis Assay

The FITC Annexin V Apoptosis Detection Kit (BD Biosciences, USA) was employed to perform cell apoptosis assay according to the manufacturer's instructions. 10^5^ cells were plated into 6-well plates and transfected with siRNAs or plasmid the following day. After 48 hours of transfection, FACS caliber flow cytometry (BD Biosciences, USA) was used to assess apoptotic rate. The sum of early and late apoptotic cells was measured. Unstained-isotype cell was used as negative control (Figure S1).

### 2.8. Cell Migration Assay

Transwell chamber inserts (BD Biosciences, USA) were used to perform cell migration assay. 10^5^ cells were seeded in the upper chambers with serum-free medium while the lower chambers were in 500 *μ* l medium with 10% FBS and the cells were incubated for 24 h. Cells that migrated to the lower chamber were fixed and stained with 0.1% crystal violet. All experiments were executed in threefold.

### 2.9. RNA Sequencing

Total RNAs from Hep3B cells transfected with si-NC or si-SNHG17-1 were isolated by TRIzol reagent (TaKaRa, Japan), and the quality and quantity met the following standards: OD260/280 = 1.8–2.2, OD260/230 ≥ 2.0, RIN ≥ 6.5, and 28S : 18S ≥ 1.0 and >10 *μ*g. Then, 20 *μ*l of high-quality RNAs from each sample was sent for RNA sequencing on the Illumina HiSeq2000 platform. The clean reads were then aligned to the human genome (version GRCh38) using the HISAT2 [20, 21]. STRING TIE [21] was used to count genes. We employed the DESeq2 algorithm to filter the differentially expressed genes after the significant analysis and *P* value under the following criteria: (1) ≥2-fold change and (2) *P* < 0.05. KOBAS 3.0 (https://kobas.cbi.pku.edu.cn/index.php) and DAVID (version 6.8, https://david.ncifcrf.gov/) online tools were performed for functional and pathway enrichment, including Kyoto Encyclopedia of Genes and Genomes [22] (KEGG) pathway and Gene Ontology [23] (GO) enrichment analyses. All the RNA-seq data have been uploaded in the GEO dataset (GSE152256).

### 2.10. Statistical Analysis

All data were analyzed with GraphPad Prism 6 (GraphPad, USA). All results were displayed as means ± SD. Student's *t*-test with two-tailed or two-way ANOVA was utilized to determine the differences between groups. The log-rank test was employed to compare survival rates. A *P* value of <0.05 was considered to have statistical significance.

## 3. Results

### 3.1. SNHG17 Expression Is Upregulated in HCC Tissues and Cell Lines Compared with Controls

**Figure 1:**
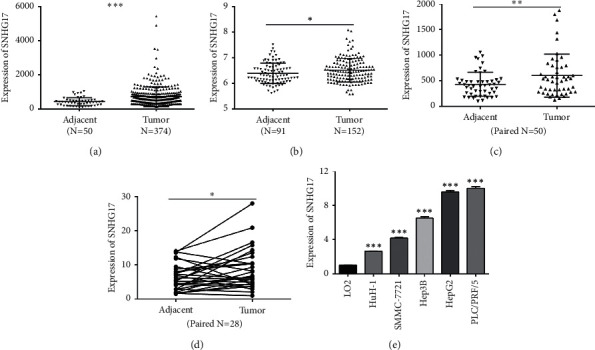
SNHG17 expression was upregulated in HCC tissues and cell lines. (a) SNHG17 was upregulated in HCC tissues (*n* = 374) compared with adjacent tissues (*n* = 50) in the TCGA-LIHC dataset. (b) SNHG17 was upregulated in HCC tissues (*n* = 152) compared with adjacent tissues (*n* = 91) in the GSE102079 dataset. (c) SNHG17 was upregulated in HCC tissues compared with corresponding nontumorous tissues in the TCGA-LIHC dataset (*n* = 50). (d) SNHG17 was upregulated in HCC tissues compared with corresponding nontumorous tissues (*n* = 28), which was examined by qRT-PCR. (e) Expression levels of SNHG17 were examined in HCC cell lines and normal hepatic epithelium cell line (LO2). ^*∗*^*P* < 0.05, ^*∗∗*^*P* < 0.01, and ^*∗∗∗*^*P* < 0.001.

To address the function of SNHG17 in HCC, we first examined the expression of SNHG17 in HCC tissues and adjacent tissues from two online-available datasets downloaded from TCGA-LIHC and GEO (GSE102079). As elucidated in Figures 1(a) and 1(b), SNHG17 were significantly upregulated in HCC tissues compared to adjacent tissues in both TCGA-LIHC (*P* < 0.001) and GSE102079 datasets (*P* < 0.05). Moreover, 35 of 50 HCC tissues were with elevated SNHG17 expression compared to paired adjacent tissues (Figure 1(c), *P* < 0.01, *n* = 50). Moreover, by performing qRT-PCR, we found the expression of SNHG17 in HCC tissues was consistently upregulated compared to that in paired noncancerous tissues (*P* < 0.05, Figure 1(d), *n* = 28). Furthermore, the level of SNHG17 in HCC cell lines was higher than that in LO2 (Figure 1(e)).

### 3.2. SNHG17 Increases Cell Proliferation in HCC Cells

**Figure 2:**
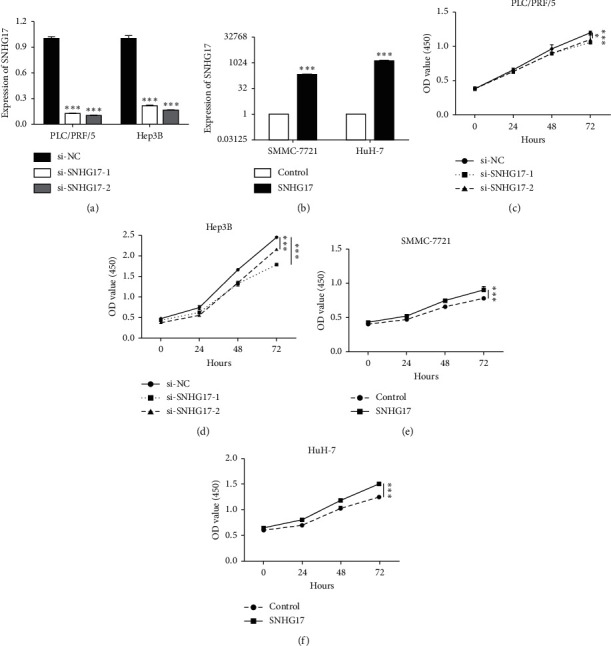
SNHG17 promoted cell proliferation of HCC. (a-b) SNHG17 expression level was determined by qRT-PCR in HCC cells as indicated. (c-d) CCK8 assays were employed to detected the effect of SNHG17 on the cell viability of PLC/PRF/5 and Hep3B cells. (e-f) CCK8 assays were employed to detect the effect of SNHG17 on the cell viability of SMMC-7721 and HuH-7 cells. ^*∗*^*P* < 0.05 and ^*∗∗∗*^*P* < 0.001.

For assessing the role of SNHG17 in HCC, SNHG17 was silenced in PLC/PRF/5 and Hep3B and overexpressed in SMMC-7721 and HuH-7. After transfection was accomplished for 48 h, qRT-PCR was used to reveal that the expression of SNHG17 was obviously decreased in both PLC/PRF/5 and Hep3B cells and increased in SMMC-7721 and HuH-7 cells (Figures 2(a) and 2(b), *P* < 0.001). As shown in Figures 2(c)–2(f), knockdown of SNHG17 notably repressed cell proliferation in PLC/PRF/5 and Hep3B cells (*P* < 0.05) while overexpression of SNHG17 significantly promoted cell proliferation of SMMC-7721 and HuH-7 cells (*P* < 0.001). Overall, SNHG17 promotes the cell proliferation ability of HCC cells.

### 3.3. SNHG17 Accelerates Cell Migration in HCC Cells

**Figure 3:**
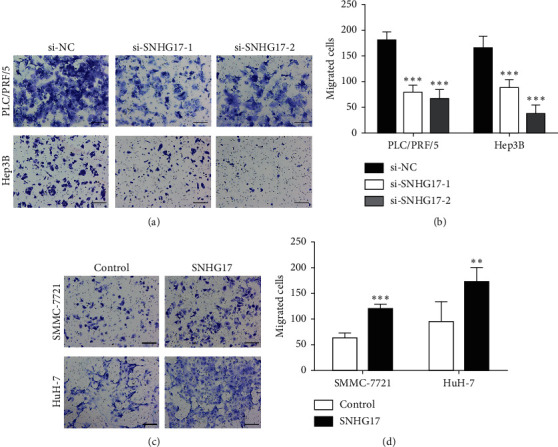
SNHG17 promoted cell migration of HCC. (a) The representative images of transwell assay in PLC/PRF/5 and Hep3B cells (magnification: 100X). (b) Quantitative data of transwell results in PLC/PRF/5 and Hep3B cells. (c) The representative images of transwell assay in SMMC-7721 and HuH-7 cells. Scale bar represents 200 pixels. (d) Quantitative data of transwell results in SMMC-7721 and HuH-7 cells. ^*∗∗*^*P* < 0.01 and ^*∗∗∗*^*P* < 0.001.

The role of SNHG17 in HCC metastasis was examined by transwell assays. The results showed that SNHG17 knockdown inhibited cell migration of PLC/PRF/5 and Hep3B cells (Figures 3(a) and 3(b), *P* < 0.001). Meanwhile, as presented in Figures 3(c) and 3(d), overexpression of SNHG17 significantly facilitated SMMC-7721 and HuH-7 cell migration (*P* < 0.01). Moreover, overexpression of SNHG17 significantly promoted the invasion ability of Huh-7 HCC cells (Figure S2). These results suggest that SNHG17 accelerates cell migration of HCC cells.

### 3.4. SNHG17 Inhibits Apoptosis in HCC Cells

**Figure 4:**
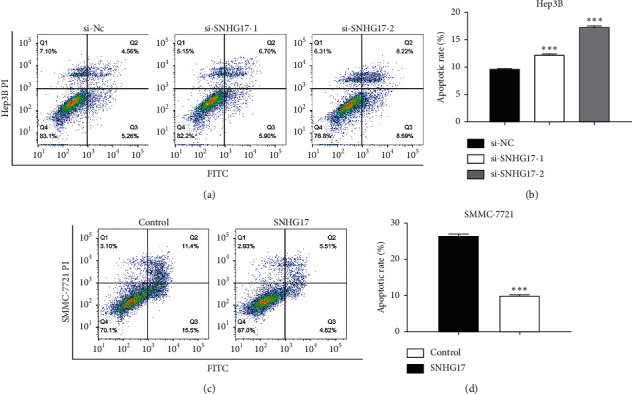
SNHG17 inhibited cell apoptosis of HCC. (a-b) Flow cytometry assays were employed to analyze the function of SNHG17 in apoptosis of Hep3B cells. (c-d) Flow cytometry assays were employed to analyze the function of SNHG17 in apoptosis of SMMC-7721. ^*∗∗∗*^*P* < 0.001.

For determining the effect of SNHG17 on HCC apoptosis, flow cytometric analysis was utilized. After transfection for 48 h, the FACS analyses were executed, and as presented in Figures 4(a) and 4(b), knockdown of SNHG17 obviously induced apoptosis of Hep3B cells transfected with si-SNHG17 (*P* < 0.001). Furthermore, overexpression of SNHG17 inhibited the apoptosis in SMMC-7721 cells (Figures 4(c) and 4(d), *P* < 0.001). These results demonstrated that SNHG17 may inhibit apoptosis of HCC cells.

### 3.5. RNA Sequencing and Bioinformatics Analysis Explore the Downstream Genes Regulated by SNHG17 in HCC

**Figure 5:**
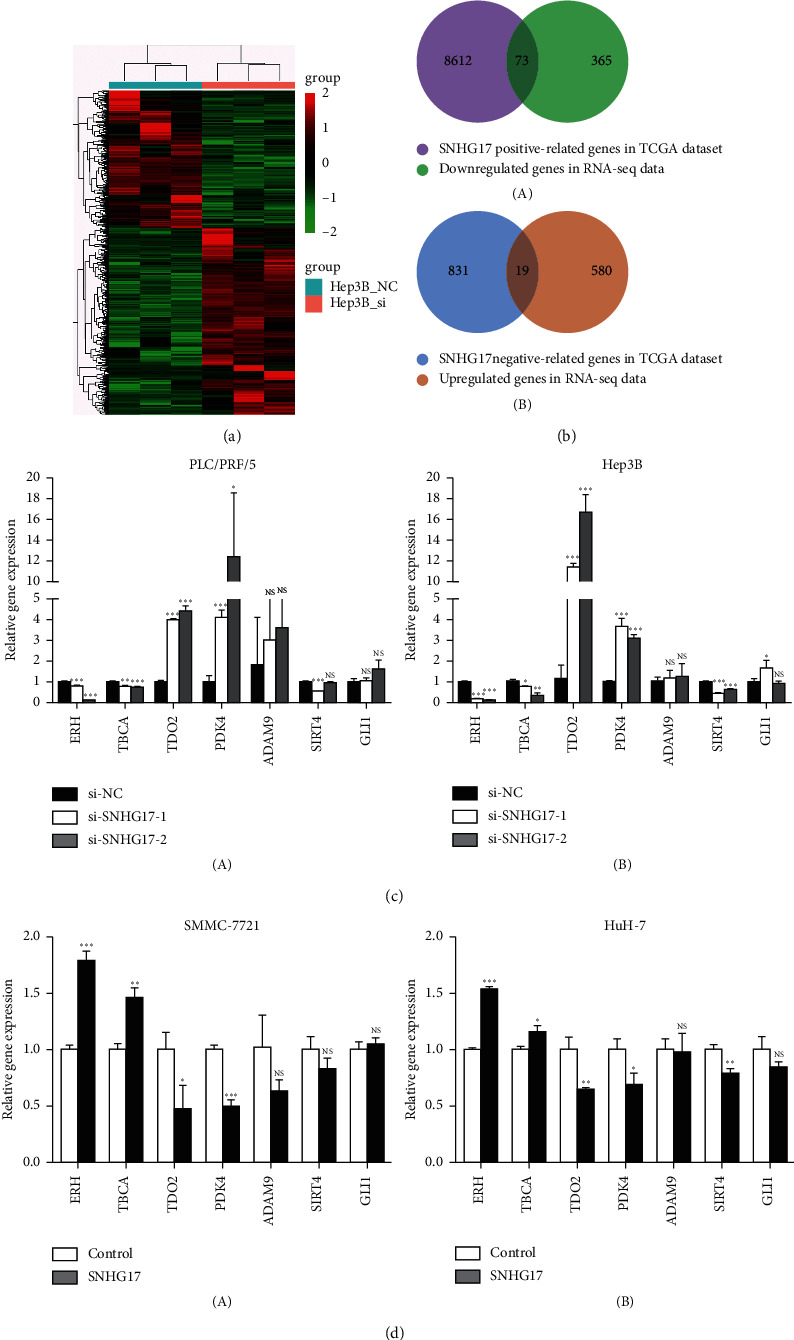
RNA sequencing and bioinformatics analysis explore the downstream genes regulated by SNHG17 in HCC. (a) Mean-centered, hierarchical clustering of 1037 genes altered (≥2-fold change, *P* < 0.05) after knockdown of SNHG17 in Hep3B cells, with three repeats. (b) The (A) Venn diagrams represent the overlap of downregulated genes in RNA-seq data and SNHG17-positive-related genes in TCGA-LIHC. The (B) Venn diagrams represent the overlap of upregulated genes in RNA-seq data and SNHG17-negative-related genes in TCGA-LIHC. (c) qRT-PCR was performed to detect the expression of indicated genes in (A) PLC/PRF/5 and (B) Hep3B cells. (d) qRT-PCR was performed to detect the expression of indicated genes in (A) SMMC-7721 and (B) HuH-7 cells. ^*∗*^*P* < 0.05, ^*∗∗*^*P* < 0.01, ^*∗∗∗*^*P* < 0.001, and NS, not significant.

To examine the molecular mechanism underlying the function of SNHG17 in HCC, we performed RNA sequencing of gene expression profiles of Hep3B cells transfected with si-NC or si-SNHG17-1. 599 genes were upregulated while 438 genes were downregulated after SNHG17 silenced by si-SNHG17 in HCC (≥2-fold change, *P* < 0.05, Figure 5(a) and Table S1). Furthermore, to further interpret the function of these genes in HCC, KEGG and GO annotations were performed. As shown in Figure S3 and Table S2, enriched pathways in KEGG included metabolic pathways, the PI3K-Akt signaling pathway, herpes simplex virus 1 infection, and cell adhesion molecules (CAMs). Regarding GO, these differentially expressed genes were predominantly enriched in cell adhesion, regulation of cell proliferation, and positive regulation of the apoptotic signaling pathway (Figure S4 and Table S3).

Moreover, Pearson correlation analysis was used to find SNHG17-related genes in HCC tissues in the TCGA-LIHC dataset. A total of 9535 SNHG17-related genes with relationship index over 0.2 were found in HCC tissues, and interestingly, among these genes, 92 were overlapped with 1037 differentially expressed genes in Table S1 (Figure 5(b) and Table S4). Seven genes identified above were then selectively validated using qRT-PCR. As shown in Figure 5(c), ERH and TBCA were downregulated after knockdown of SNHG17 in both PLC/PRF/5 and Hep3B cells (*P* < 0.05) while TDO2 and PDK4 were upregulated (*P* < 0.05). Subsequently, ERH and TBCA were upregulated in SMMC-7721 and HuH-7 with overexpression of SNHG17 (*P* < 0.05) while TDO2 and PDK4 were downregulated (Figure 5(d), *P* < 0.05). Moreover, the expression levels of ERH and TBCA were significantly upregulated in HCC tissues (Figure S5) while TDO2 and PDK4 were significantly downregulated in HCC tissues in both TCGA-LIHC and GEO datasets (Figure S6).

### 3.6. SNHG17 Predicts a Poor Prognosis of HCC

**Figure 6:**
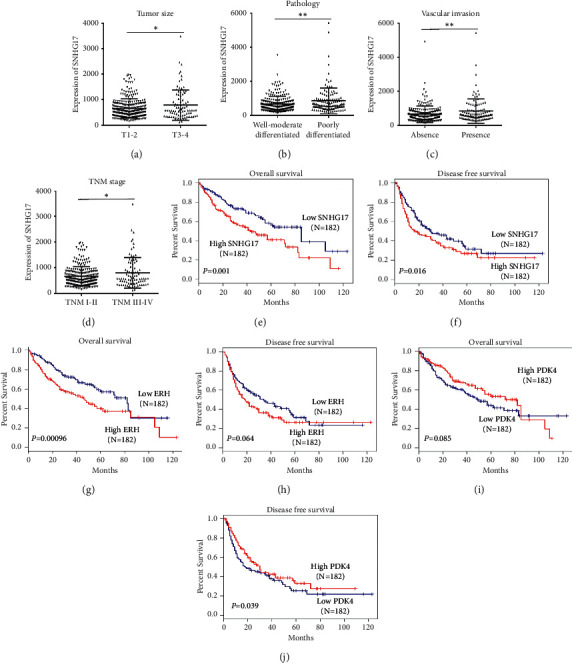
SNHG17 was correlated with poor prognosis of HCC. (a–d) Elevated SNHG17 expression was correlated with larger tumor size, poor differentiation, the presence of vascular invasion, and advanced TNM stage in HCC patients. (e-f) Kaplan–Meier curves for SNHG17 in HCC. (g-h) Kaplan–Meier curves for ERH in HCC. (i-j) Kaplan–Meier curves for PDK4 in HCC. ^*∗*^*P* < 0.05 and ^*∗∗*^*P* < 0.01.

Subsequently, to understand the clinical significance of SNHG17 in HCC, the association between SNHG17 expression and patients' prognosis and clinicopathological characteristics was evaluated. Results of this evaluation showed that higher SNHG17 expression was obviously correlated with larger tumor size, poor differentiation, the presence of vascular invasion, and advanced TNM stage (Figures 6(a)–6(d)). Furthermore, Kaplan–Meier survival analysis revealed that patients in the SNHG17-high group displayed a remarkably shorter OS (*P* < 0.01, Figure 6(e)) and DFS (*P* < 0.05, Figure 6(f)). Moreover, the expression of SNHG17 was significantly associated with overall survival in HCC as indicated by univariate (HR = 1.257, 95% CI = 1.046–1.511, *P*=0.015) and multivariate (HR = 1.229, 95% CI = 1.025–1.474, *P*=0.026) Cox regression analyses (Figure S7). Broadly, these findings suggested that SNHG17 was an independent prognosis predictor of HCC patients. Interestingly, as shown Figures 6(g)–6(j), HCC patients in the ERH-lower group had significantly longer OS (*P* < 0.001) and DFS (*P*=0.06) while patients in the PDK4-lower group had shorter OS (*P*=0.08) and significantly shorter DFS (*P* < 0.05), which was also validated by univariate and multivariate Cox regression analyses (Figures S8 and S9).

## 4. Discussion

Accumulating evidence showed the great significance of lncRNAs in tumorigenesis and progression of HCC, such as HOTAIR, MEG3, Lnc-SchLAH [11–13]. Apart from the well-characterized lncRNAs, the biological functions and potential mechanisms of most lncRNAs in HCC remain unclear [24, 25]. In this study, we discovered LncRNA SNHG17 was significant upregulated in HCC.

Previous studies have discovered SNHG17 plays an important role in the development of cancer, such as colorectal cancer, non-small-cell lung cancer, gastric cancer, breast cancer, melanoma, and glioma [8–10]. However, the role and molecular mechanisms of SNHG17 in HCC carcinogenesis remain unclear. Our present study showed that SNHG17 was significantly upregulated in both public databases and collected HCC tissues and HCC cell lines. Furthermore, elevated SNHG17 expression was noticeably associated with larger tumor size, poor differentiation, the presence of vascular invasion, advanced TNM stage, and poor prognosis, indicating SNHG17 may be an oncogene which predicts a poor prognosis of HCC patients. Gain- and loss-of-function experiments proved that SNHG17 promotes cell proliferation and migration and inhibits apoptosis of HCC, which confirmed the function of SNHG17 in HCC. However, as lack of predictors and prognostic indicators for HCC patients treated with immune checkpoint inhibitors (ICIs) or tyrosine kinase inhibitors (TKIs), HCC patients with ICIs or TKIs treatment history should be included for treatment response prediction regarding SNHG17 expression in future study, which would help clinicians for the choice of therapeutic method and daily management [26]. Since the BRAF pathway played a marked role in hepatocellular carcinoma, future studies may be needed to explore the relationship between the SNHG17 and BRAF pathway [27].

To explore the specific mechanism of SNHG17 in HCC, RNA sequencing was performed, and 1037 differentially expressed genes were found caused by knockdown SNHG17; among them, 92 were overlapped with SNHG17-related genes in the TCGA-LIHC dataset. According to KEGG and GO annotation, SNHG17 might influence the occurrence and progression of HCC by modulating several pathways, including metabolic pathways, the PI3K-Akt signaling pathway, cell adhesion, proliferation, and the apoptotic signaling pathway. Furthermore, ERH, TBCA, TDO2, and PDK4 genes were successfully validated.

Interestingly, ERH and TBCA, as the positive SNHG17-related gene, were also upregulated in HCC, and ERH also predicts a poor prognosis for HCC patients. ERH is critically required for genomic stability and cancer cell survival by regulating cell cycle through its mRNA splicing activity [28]. Weng et al. reported that ERH was upregulated in HCC and played a role as a regulator of DNA damage response genes [29], which is consistent with our results. TBCA has been found to regulate progression, invasion, and metastasis of clear cell renal cell carcinoma [30]. Subsequently, TDO2 and PDK4, as the negative SNHG17-related gene, were downregulated in HCC; moreover, PDK4 also predicts a relative better prognosis for HCC patients. Moreover, Strowitzki et al. reported that high hepatic expression of PDK4 improves the survival of multimodal treatment of colorectal liver metastasis [31], while Bai et al. found TDO2 was downregulated in liver cancer [32]. However, the role and underlying mechanism of TBCA, PDK4, and TDO2 in HCC have not yet been illustrated and deserve further study.

RNA sequencing and bioinformatics analysis of a public dataset could partially elucidate the mechanism by which SNHG17 regulates proliferation, migration, and apoptosis of HCC and provide a novel method for measuring the potential mechanism. However, further studies such as RNA pull down, mass spectrometry, rescue experiments, and western blot are needed to explore the downstream mechanism of SNHG17. Nevertheless, results may be different between experiments in vivo and in vitro, due to some causes such as the tumor microenvironment, and animal study is needed to further explore the role of SNHG17 in vivo.

## 5. Conclusions

Our studies confirmed that SNHG17, which is upregulated in HCC, promotes cell proliferation and migration and inhibits apoptosis and predicts a poor prognosis of HCC. By employing RNA sequencing, we preliminary explore the genes and pathways regulated by SNHG17 in HCC. Hence, our findings illustrate that SNHG17 acts as a noncoding oncogene in HCC progression and explores its potential target genes in HCC.

## Data Availability

The datasets analyzed and/or used in this current study can be obtained by requesting the corresponding author.

## Supplementary Materials

Supplementary Materials Supplementary figure legends: Figure S1. Unstained-isotype control of Hep3B (A) and SMMC-7721 (B). Figure S2. SNHG17 promoted cell invasion of HCC. (A) The representative images of transwell assay in HuH-7 cell (magnification: 100X). (B) Quantitative data of transwell results in HuH-7 cells. ^*∗∗∗*^*P* < 0.001. Figure S3. Distribution of KEGG terms for 1037 genes altered (≥2-fold change, *P* < 0.05) after knockdown of SNHG17 in Hep3B cells. Figure S4. Distribution of GO terms, including molecular function, biological process, and cellular component, for 1037 genes altered (≥2-fold change, *P* < 0.05) after knockdown of SNHG17 in Hep3B cells. Figure S5. The expression levels of ERH (A) and TBCA (B) in the TCGA-LIHC and GSE102079 HCC dataset. Figure S6. The expression levels of TDO2 (A) and PDK4 (B) in the TCGA-LIHC and GSE102079 HCC dataset. Figure S7. Univariate and multivariate Cox regression analyses of SNHG17 expression in HCC regarding overall survival. Figure S8. Univariate and multivariate Cox regression analyses of ERH expression in HCC regarding overall survival. Figure S9. Univariate and multivariate Cox regression analyses of PDK4 expression in HCC regarding overall survival. Supplementary tables: Table S1. The 1037 genes altered (≥2-fold change, *P* < 0.05) after knockdown of SNHG17 in Hep3B cells, with three repeats by RNA sequencing. Table S2. The list of KEGG terms for 1037 genes altered (≥2-fold change, *P* < 0.05) after knockdown of SNHG17 in Hep3B cells. Table S3. The list of GO terms for 1037 genes altered (≥2-fold change, *P* < 0.05) after knockdown of SNHG17 in Hep3B cells. Table S4. The overlap of SNHG17-related genes in RNA-sequencing results and HCC tissues (TCGA-LIHC).
