# Supplementary material for: LncRNA SNHG17 Contributes to Proliferation, Migration, and Poor Prognosis of Hepatocellular Carcinoma
Source: Can J Gastroenterol Hepatol. 2021 Sep 14;2021:9990338. doi: 10.1155/2021/9990338 (PMC8455207; doi:10.1155/2021/9990338)
Supplement: Supplementary Materials — Supplementary figure legends: Figure S1. Unstained-isotype control of Hep3B (A) and SMMC-7721 (B). Figure S2. SNHG17 promoted cell invasion of HCC. (A) The representative images of transwell assay in HuH-7 cell (magnification: 100X). (B) Quantitative data of transwell results in HuH-7 cells. ∗∗∗P < 0.001. Figure S3. Distribution of KEGG terms for 1037 genes altered (≥2-fold change, P < 0.05) after knockdown of SNHG17 in Hep3B cells. Figure S4. Distribution of GO terms, including molecular function, biological process, and cellular component, for 1037 genes altered (≥2-fold change, P < 0.05) after knockdown of SNHG17 in Hep3B cells. Figure S5. The expression levels of ERH (A) and TBCA (B) in the TCGA-LIHC and GSE102079 HCC dataset. Figure S6. The expression levels of TDO2 (A) and PDK4 (B) in the TCGA-LIHC and GSE102079 HCC dataset. Figure S7. Univariate and multivariate Cox regression analyses of SNHG17 expression in HCC regarding overall survival. Figure S8. Univariate and multivariate Cox regression analyses of ERH expression in HCC regarding overall survival. Figure S9. Univariate and multivariate Cox regression analyses of PDK4 expression in HCC regarding overall survival. Supplementary tables: Table S1. The 1037 genes altered (≥2-fold change, P < 0.05) after knockdown of SNHG17 in Hep3B cells, with three repeats by RNA sequencing. Table S2. The list of KEGG terms for 1037 genes altered (≥2-fold change, P < 0.05) after knockdown of SNHG17 in Hep3B cells. Table S3. The list of GO terms for 1037 genes altered (≥2-fold change, P < 0.05) after knockdown of SNHG17 in Hep3B cells. Table S4. The overlap of SNHG17-related genes in RNA-sequencing results and HCC tissues (TCGA-LIHC). [file 9990338.f1.zip › 9990338.f1/Table S1 (2).pdf]

Gene_Symbol	log2FoldChange	P value
AC009086.2	21.88161751	2.16E-08
AL158066.1	21.54480369	3.54E-08
AC138696.1	20.99742419	7.83E-08
AC068234.1	19.98331599	3.21E-07
AC006547.3	9.28990865	0.017435392
ZFP91-CNTF	9.125988496	0.019523274
BORCS7-ASMT	9.042662677	1.73E-12
MAT1A	7.86381279	1.84E-08
BIVM-ERCC5	7.723711157	0.002634526
TBC1D3I	7.664990369	0.049916186
COL25A1	6.904215429	1.31E-05
LINC02015	6.901184146	5.44E-05
MMP23B	6.643018529	8.98E-05
STRC	6.587360344	0.001717561
AC022028.2	6.418000023	0.003838423
C1QTNF9	6.352797033	0.000353348
PAQR9	6.304115748	0.000564449
TRIM6-TRIM34	6.267886078	0.018047157
AC005154.6	6.267886078	0.018047157
ABCA17P	6.265579516	0.000357027
AC009927.1	6.248229114	0.004775535
OLFML3	6.247519332	0.0007734
NPPC	6.213535639	0.000589468
DHH	6.200957124	0.004985836
LINGO1	6.195195346	0.000814455
PPFIA2	6.154671373	0.000618489
HULC	6.150096109	0.000613342
BTN1A1	6.143599822	0.000812209
AL139039.1	6.05990446	0.007836045
AL445483.1	6.034844168	0.001308044
CLEC20A	6.016680854	0.001169187
AP001107.5	5.984186624	0.001792001
TONSL-AS1	5.982998315	0.001386242
AL136309.2	5.962105327	0.012003639
GPR34	5.939073524	0.011234022
AC008982.1	5.938444456	0.000105183
AL713999.1	5.932115905	0.002193463
SLC4A10	5.929284059	0.011238256
ZNF98	5.912636184	0.001918073
RPL12P28	5.910630377	0.036013299
PDCD6IPP1	5.910630377	0.036013299
AP003419.4	5.867269364	0.003713502
AC009336.3	5.852353717	0.002683813
CLPSL2	5.835033066	0.012462822
LINC01885	5.832232419	0.013438427
SORCS1	5.828712438	0.002581586

AC068594.1	5.794297738	0.003553538
AC023824.7	5.774913667	0.017597086
AC100803.1	5.730316682	0.003393977
IL1R2	5.718715707	0.016401253
AP001062.3	5.698815983	0.017165549
CCDC197	5.696872061	0.022847477
SMTNL1	5.692130679	0.007096106
IGFBP7-AS1	5.686354647	3.73E-05
AC025165.1	5.68613588	0.018416123
MOBP	5.682735248	0.004045027
TMEM150C	5.67094482	0.01840496
AC009097.1	5.657768333	0.02225692
DHRS9	5.650385328	0.019171546
AC005034.4	5.650385328	0.019171546
TRIM60P18	5.643302262	0.005725363
AC091868.1	5.622494012	0.006831229
TEKT3	5.616624307	0.024736059
AC025287.1	5.582276685	0.034530666
COL6A5	5.56198328	0.02593279
CCDC54	5.52354198	0.014134553
AC005052.1	5.521125584	0.026790286
AC100788.2	5.515961545	0.008329946
NKAIN4	5.511645574	0.007409293
AC145423.2	5.497074424	0.010929524
CATSPERD	5.494693324	0.008607165
TGM7	5.484142161	0.029490296
AC145285.4	5.462218661	0.029904723
AC023644.1	5.453679064	0.000964561
FBXL7	5.446766033	0.031506484
HSD17B13	5.438502665	0.030450388
AL591475.1	5.421616813	0.033688058
TRIM63	5.414292549	0.032628133
AC084782.3	5.413407966	0.038775279
LY96	5.384794007	0.0112717
CCDC81	5.374248692	0.039207909
AC012512.1	5.366290539	0.040866487
AC021549.1	5.35700454	0.012673476
MAGEB3	5.353472477	0.040095772
PSMD4P1	5.351620354	0.039254588
HSPB2	5.32817065	0.016846411
HIST1H1T	5.326057192	0.038625353
HOXA11-AS1_2	5.32578304	8.57E-07
AC005096.1	5.312196437	0.041126804
RPL12P42	5.30907623	0.040749842
SOX5	5.298231107	0.015138543
CD80	5.287452129	0.042528967
SLAMF7	5.283038949	0.044453849

AC244102.1	5.269554114	0.046571337
AL359551.1	5.267450588	0.044350583
AL161719.1	5.263367686	0.01980768
AC113607.1	5.256221408	0.044100224
AC026464.2	5.254708065	0.000664458
LINC02404	5.228795705	0.046508961
AL158196.1	5.194613743	0.022744227
COX6CP2	5.166139458	0.02088942
AC113139.1	5.148027915	0.024091138
MIAT_exon5_3	5.118857111	0.026844056
GPR18	5.038410439	0.029998544
ZRANB2-AS1	5.021415702	0.000197956
AP002812.1	4.990188662	0.042919269
AL359853.2	4.958467427	0.033991396
CPLX2	4.933703676	0.036429331
ANKRD62	4.914804862	0.038021771
SNORD16	4.896055802	0.002740518
SIGLEC9	4.858814096	0.04204612
AC010240.2	4.84775439	0.044291928
AC093901.1	4.811897547	0.049884461
AC009163.3	4.791365262	0.048957682
AC100788.1	4.707336448	0.001218066
AC107982.1	4.699049086	0.021052575
AC010997.3	4.688126563	0.012065856
AC064801.1	4.605980955	0.023516864
PRLR	4.569906829	0.00028642
DIO1	4.523091369	0.021299116
SNORA58B	4.438954951	0.002412917
CCDC177	4.341589682	6.29E-05
SNORA1	4.269323956	0.006403504
AP001527.1	4.186251328	0.043801947
AC090617.6	4.17484569	8.13E-09
AC011489.1	4.144400937	1.91E-19
FYB1	3.88974542	0.003044312
RAB28P5	3.841208874	0.032206211
AL645465.1	3.831429233	0.001707125
LINC02009	3.780838718	0.00945867
TUBG1P	3.771007689	0.009751236
AL450326.1	3.75153097	0.005210336
RGS4	3.718072848	0.003753264
CLDN14	3.703682929	0.045474152
MYH16	3.701647261	0.008937936
AC087190.4	3.693886165	0.011464472
PLA1A	3.572487879	0.014982277
MAGI2-AS3	3.572087209	0.015561416
AC012254.2	3.463565284	0.01897132
RHCG	3.4621248	0.004561095

RN7SL832P	3.440745555	0.015793148
AL354919.2	3.43359894	0.022857141
IGDCC4	3.421472423	0.001863846
MAGEB18	3.401286321	0.035327838
HSPD1P11	3.400630701	0.046472687
HMGA1P8	3.321667526	0.001000878
AP002840.2	3.27227505	0.041470562
NME9	3.240677291	0.019847689
TNFRSF14	3.224846026	2.05E-61
VCX	3.195032792	0.010396094
PAEP	3.179096543	0.000396156
TP63	3.178323112	3.26E-05
AC026471.6	3.10479915	0.010639422
AC020907.1	3.029949364	0.006421475
AC037198.3	2.985980478	0.002720408
PNMA6A	2.940177737	0.000341854
KLHL10	2.918651098	0.036908764
NRIP3	2.903314454	2.34E-07
AC110015.1	2.858106829	0.017952511
RN7SL657P	2.84360587	0.033763473
FRMPD4	2.831968167	0.032371021
RHAG	2.8078256	0.006552119
AC004637.1	2.729609268	0.020712887
AC021028.1	2.705362565	0.037756715
PDXDC2P	2.700677124	0.017390108
LINC01293	2.68990238	0.025089666
AC121761.2	2.680081006	0.026189107
HTR3A	2.661732056	0.000564351
AC126603.1	2.661319384	0.027592503
ABHD14A-ACY1	2.649379043	0.014488888
CLVS1	2.631937237	0.008137735
DCLK1	2.581598489	0.007352646
HOXD4	2.579892049	0.002678799
SCN2A	2.561192707	0.001045339
AL049830.3	2.560166872	1.28E-05
AC245096.1	2.558653497	0.009616977
HLA-J	2.543106405	0.046386462
PLEKHB1	2.541910306	0.001023301
AL049697.1	2.509950768	0.001606759
AC138028.6	2.509538673	0.010994204
DLGAP3	2.492204065	0.008879647
AC015818.2	2.490529128	0.041202628
AC106820.3	2.485935113	0.021550859
TNFRSF14-AS1	2.479804782	1.50E-26
AC104452.1	2.463591356	0.001447395
CATSPERG	2.45720186	0.007241013
CELF5	2.454997855	0.031746064

AC120498.9	2.430892897	0.001817044
AGTR1	2.420062164	0.000186525
AC007731.5	2.383420012	0.014739798
SOX21-AS1	2.350214769	0.028385274
BHLHB9	2.347631477	7.35E-05
TDO2	2.337217199	0.018801704
RN7SL751P	2.324092771	0.035845937
MYO7A	2.323237738	0.000102718
BMS1P4	2.31941077	3.18E-06
PACSIN1	2.289110028	1.09E-07
GAGE12H	2.287282889	0.022182338
AL117329.1	2.280848426	0.034867737
CRISPLD1	2.264899627	0.00404762
LINC02274	2.259318669	0.004294675
AC110597.3	2.258143375	0.023800527
ZNF724	2.242834936	1.46E-15
LINC02535	2.241079849	0.000188059
SCHIP1	2.239876452	0.007284685
AC122129.1	2.234721443	0.002843588
ELFN2	2.233570022	0.038861777
AC005225.5	2.227464964	0.012225019
AL162258.2	2.227252407	4.51E-05
CCNT2-AS1	2.226965539	0.001790876
AREG	2.206185194	1.03E-39
HMCN1	2.179318571	4.06E-05
AC090970.3	2.161229917	0.008496253
P2RX2	2.160674451	0.035715934
PPP1R36	2.158459652	0.001540707
C11orf96	2.155869844	0.023950611
ZNF624	2.131796224	5.51E-05
MANEA-AS1	2.130400196	0.003482635
AC002470.1	2.107465281	0.028247332
AC245060.5	2.0971197	0.005951791
DDO	2.084812363	0.004379199
CYP1B1	2.060336444	2.88E-24
ZNF658B	2.041112176	0.013813271
CDRT4	2.027757289	4.45E-05
SYCE2	2.018427026	1.78E-08
AL353743.4	2.001749905	0.037407741
CDK14	1.986296765	1.64E-34
RASGRF2	1.980678188	0.048115337
PRKG1	1.94869574	0.000202636
MAPK8IP2	1.946836183	3.98E-14
AC100803.2	1.938747172	0.006138189
AC096887.1	1.919015396	0.003598064
DUX4L27	1.917764628	0.011916089
RBBP8NL	1.910546532	0.040160388

AL591684.2	1.910164579	0.039890393
AJ271736.1	1.896021807	0.030727846
LINC02029	1.877571445	0.01878968
LRRC37A11P	1.871869749	0.034254685
VCX3A	1.865551741	0.001486449
CD8A	1.8363032	0.001574851
SOBP	1.835816094	0.01030862
AL512408.1	1.826288676	0.000196254
RELN	1.819371821	0.007780266
ZNF100	1.81116056	4.67E-11
BMPER	1.804236284	0.00068207
POU5F1B	1.795395389	0.001936975
CDH5	1.79443984	5.18E-07
IL20	1.791930972	0.043275963
EGF	1.781948332	0.028291373
ACP5	1.773373024	0.009114053
AC024257.3	1.760786474	0.007568073
LINC00880	1.755742798	0.045219909
LINC02475	1.746804817	0.047932339
GPR3	1.746789896	4.29E-20
HIST1H2BJ	1.743342997	5.26E-09
AL138976.2	1.742015315	0.041897864
DLEU2_1	1.736446364	0.019081599
AC010503.4	1.734895209	0.000421915
AC093495.1	1.730542916	0.006730489
SP9	1.719625791	0.019992226
PIWIL2	1.717033481	0.031074502
CCDC150P1	1.71676432	4.96E-05
ZNF430	1.716754216	9.55E-07
AL137002.2	1.705298206	0.005451725
GAGE2A	1.70264206	0.000370044
AL117209.1	1.700712875	0.036841612
TARID	1.697380667	0.00055455
TEX14	1.696382456	2.80E-05
AC008406.3	1.695559407	0.004552669
LHX9	1.692768395	0.030514809
PTHLH	1.690344968	0.017091422
AC112220.4	1.684591242	0.00090188
RNF157	1.681556037	5.37E-07
C1QTNF2	1.67850905	2.01E-08
P4HA3	1.673784058	2.17E-06
PTN	1.66463002	0.000158959
TMEM200C	1.663160536	9.06E-05
KCNMB2-AS1	1.656173125	0.036393252
TPM4	1.655166153	2.26E-70
NAMPTP1	1.653249612	0.010115969
S1PR1	1.651685983	7.50E-13

ABCB4	1.651170617	0.015403978
AC242426.3	1.650531592	2.68E-06
AC092279.2	1.643407761	0.016248525
MXD1	1.638037681	0.021612097
ATF3	1.635620272	0.006852698
PELI2	1.62971691	0.020207679
AC009163.7	1.628658753	0.036248203
PLAU	1.624170556	0.000731587
AGT	1.621827654	2.49E-09
AC087623.4	1.618638552	0.038669359
PGM2L1	1.615335266	6.60E-17
AC008764.1	1.613274007	0.025298664
TRPM2-AS	1.610024178	9.34E-05
LINC01535	1.609913831	0.048957716
C3orf52	1.607887305	4.01E-12
AC016831.1	1.607834538	0.022062811
PTGS2	1.601122364	8.31E-46
TNC	1.600153399	7.61E-13
SLC6A14	1.599891422	0.048654938
DAND5	1.5914552	0.040806414
PCDH15	1.588885217	0.04908635
ARHGAP6	1.581592612	0.002773758
AC068768.1	1.5746918	2.46E-06
AC008894.3	1.572631962	3.51E-46
RASGEF1B	1.561060186	0.045596528
KLHL35	1.560915294	6.23E-12
PLPPR4	1.550199587	0.001937643
GANAB	1.549680924	8.21E-53
PLAT	1.527056175	1.64E-13
DLEU2_6	1.52588892	0.003131183
ANPEP	1.525088933	0.037128269
NFIX	1.520068943	0.000224206
NCAM1	1.51393806	0.006141004
ADGRF4	1.509459338	0.01051549
AC022400.6	1.508821919	0.000236859
PACERR	1.508470651	0.009063076
AC098614.1	1.499695879	0.003101473
LINC00882	1.496558028	0.01220656
COCH	1.496077769	7.33E-07
AC004477.1	1.49587274	0.013339447
LINC01556	1.494821996	0.016158391
DMC1	1.492425846	0.030168016
FNDC4	1.487692468	0.001171076
CADPS2	1.482395316	0.003829752
PRTFDC1	1.482026312	0.000131606
NR4A3	1.479189106	1.08E-14
ZNF253	1.478442485	0.005280403

VGF	1.47829605	0.000905702
AC126544.1	1.477055116	0.026773086
ZNF658	1.476796224	0.000338581
C4orf19	1.475670794	7.46E-07
NECAB1	1.47458162	0.016886355
LINC01126	1.472618009	0.038891051
ZNF616	1.468661984	4.52E-06
SPOCD1	1.460068893	3.80E-16
ZNF93	1.456721955	0.001825262
ZBTB43	1.456497319	0.023848128
KCNS3	1.454892806	0.012792647
CXCL2	1.452987003	3.75E-18
NEUROG2	1.450937089	0.022026476
EMP1	1.450098943	0.026986754
TMEM33	1.442806439	1.42E-16
AP001350.2	1.44184712	0.011966896
GEM	1.438285874	2.47E-10
DUSP10	1.438096736	3.05E-14
C1orf140	1.435440242	0.024501683
USP51	1.435413382	2.13E-06
G3BP2	1.428428845	6.76E-33
AC017100.1	1.427934605	0.041715551
STX12	1.42788391	3.83E-14
MESP2	1.427784866	0.000531486
SEPT4	1.421104306	0.022013194
AC092720.1	1.418285145	0.000979613
ZNF184	1.412327378	8.20E-18
AL162377.1	1.398359039	0.003671565
VASH2	1.394053872	0.005516828
ZNF14	1.381251972	0.024712814
CRYAB	1.380363014	0.040755711
CXCL8	1.372620904	2.62E-13
VDAC1P8	1.372457739	1.69E-05
ZNF714	1.372016431	6.76E-05
TAPT1-AS1	1.367517114	0.028642096
AL162595.1	1.36574469	0.047315346
MEMO1P1	1.365067171	0.040000172
SLC25A21	1.363739938	0.026883541
SYT11	1.358178729	0.042366696
MRAS	1.352793519	1.65E-11
OLFML2B	1.352268866	0.031085747
RPL18P13	1.351932449	0.020308961
LGALS8-AS1	1.348298152	0.036594173
AC004803.1	1.346498551	0.013212975
LYPD6	1.342734376	8.33E-09
RNF185	1.340646585	0.020234873
FGB	1.339049871	0.027740336

AL513523.1	1.333980713	0.049799956
RYR2	1.330161452	2.62E-05
ZNF736	1.327260391	0.000670985
HIST1H2BD	1.325199711	4.42E-08
AC087499.1	1.324830421	0.003276344
SGIP1	1.320684169	0.04862434
IGFBP1	1.319889918	0.002863664
ZFAND4	1.318273197	0.00068745
TOX3	1.316324575	0.007919638
COL24A1	1.311404002	0.024217056
AL451074.2	1.31087643	0.011167717
TM4SF1	1.307684496	6.88E-23
SELPLG	1.30306109	0.005507989
LINC01116	1.301323224	0.020985376
ZNF91	1.299575897	0.003274926
EXOC3-AS1	1.297136405	6.82E-12
ZNF204P	1.296140887	0.040267945
ZNF154	1.294930145	0.007371452
GNPDA1	1.292257215	9.34E-21
RDH5	1.29038837	0.00651346
YEATS2-AS1	1.288822672	0.004263747
AL135838.1	1.287384401	0.048780387
RNF138	1.286217857	3.63E-09
TSLP	1.282646341	0.002031934
NES	1.281526652	6.23E-10
DPY19L2P2	1.278363961	1.14E-05
INHBA	1.273883495	0.020783472
CRCP	1.272121958	0.000593647
CREB5	1.271292982	4.98E-08
SLC2A3	1.270024658	1.40E-07
LMTK3	1.267728068	0.000358589
PRSS22	1.263694514	0.0400536
EOMES	1.263013848	0.009496137
SESN2	1.262520184	4.20E-23
ERV3-1	1.261166567	0.001180821
ASB1	1.256300723	2.51E-10
ZNF25	1.253084527	0.005191609
AC092143.3	1.251935407	0.000125169
AC096733.2	1.249866877	0.00011052
ZNF426	1.244150549	2.64E-14
C19orf57	1.24383477	1.46E-07
PDK4	1.243592264	2.70E-10
MAGEA2B	1.242945814	0.005826161
NKX3-1	1.241752101	0.000435163
EFHC2	1.240412498	0.047327122
PAG1	1.239705289	4.92E-08
TRIB3	1.2393845	8.05E-27

NFATC2	1.239257357	0.005139582
CXXC4	1.239044198	0.038456552
ZNF680	1.236662938	7.31E-05
RUNDC3B	1.231264792	0.029483825
RRAGD	1.231170961	3.22E-13
SPINK1	1.227814672	0.040447653
AC074091.1	1.226683061	0.021085973
GOLT1A	1.220838088	0.002204185
AC080080.1	1.220143354	0.048351167
LINC00957	1.219706945	0.022904849
NACAD	1.218391522	0.003913802
AC133065.4	1.213552434	0.030646452
IL6R	1.212832375	2.13E-14
ZNF572	1.212763366	0.017834726
LIMK1	1.212412206	3.19E-12
LINC00518	1.211754156	0.000451284
LPP-AS2	1.206763892	0.002691522
ZNF766	1.206724065	7.27E-08
ITGA7	1.20600635	0.003657742
B4GALT1-AS1	1.205791342	2.54E-08
RRM2	1.20325458	2.04E-14
KLF6	1.202551502	3.29E-13
ZNF554	1.200891022	7.21E-06
THBS1	1.199519853	1.04E-30
AC009779.3	1.199079585	0.001815114
IGFN1	1.196517983	2.44E-06
ZDHHC16	1.194210374	8.96E-18
B4GALT4-AS1	1.192756445	0.044359044
SERPING1	1.188922824	0.011523712
RCN3	1.187615077	2.97E-09
ZMAT1	1.185776672	0.018766222
RGS2	1.182155019	2.69E-18
AL606834.2	1.182107098	0.001840236
AC073072.1	1.180475237	0.000320116
CTSH	1.178727658	7.21E-05
ZNF726	1.177561763	0.006593738
LONRF1	1.177490001	1.11E-08
THRB	1.177421882	0.000139231
SAMD12	1.176129455	0.000953426
TXNIP	1.174470853	1.02E-18
MIR503HG	1.166354707	0.002157656
GALNT13	1.164915907	0.001108508
TBC1D9	1.164190995	1.37E-17
ZNF845	1.163239529	0.000578486
FGD4	1.16321605	0.003772171
AC005332.7	1.162881334	0.000472312
SLC9A3	1.161254599	1.74E-11

TRIML2	1.159464641	1.65E-05
GEN1	1.158743124	0.001198685
ZNF429	1.157988367	0.042944524
AC015660.1	1.157679775	0.017027418
RASAL2	1.157122136	6.75E-18
MIATNB	1.156864947	0.020061266
ZNF528-AS1	1.154286223	0.04278525
C3orf67	1.153333847	0.007464841
PERP	1.152624239	1.64E-08
AC006252.1	1.148949249	0.026501915
TMX3	1.146931425	1.09E-06
ADAMTS3	1.146234556	0.013554745
BAALC-AS1	1.145741643	0.043465803
MOXD1	1.145362678	0.000331068
SCARB2	1.145102015	2.80E-19
GNA11	1.143981643	2.65E-06
AP000695.2	1.143691902	0.043515173
CHST14	1.142431442	1.47E-18
SNX10	1.1395065	3.22E-07
AL355001.2	1.139153024	5.60E-05
RGS19	1.138174329	6.65E-14
NTN3	1.136751157	8.85E-09
PARM1	1.135173424	0.01930262
C1QTNF6	1.131547019	2.15E-05
AC093010.3	1.130520277	0.001534379
FAM173B	1.12991812	2.74E-10
NLGN4X	1.12945116	0.003056901
TTC33	1.128587012	1.48E-16
AC073508.2	1.128482626	0.000109914
LINGO2	1.12831956	0.0003365
IKZF2	1.126160991	0.00401998
LINC02228	1.126046829	0.044257404
ZNF134	1.125472021	7.61E-08
AL358472.4	1.122601804	0.004237564
HSD17B14	1.122317254	0.034210107
AL158151.3	1.121392887	0.003334553
DGAT2	1.120798763	7.43E-06
GPR173	1.118303804	0.014691449
EPDR1	1.116017685	8.19E-12
HIVEP3	1.11528592	2.13E-05
C18orf54	1.114542016	7.60E-06
CTSL	1.113404776	1.49E-26
HIST1H4H	1.113368401	0.002150957
RDM1	1.112128482	0.013093549
MEX3D	1.111719734	3.98E-20
LBX2-AS1	1.108800258	0.000332849
FICD	1.106929318	3.33E-12

AC092645.1	1.105513915	3.22E-05
SNAI2	1.103652079	1.42E-15
FOXP4	1.102903215	1.20E-18
ZNF860	1.101330502	1.31E-06
GALNT3	1.101244091	1.23E-16
CLUHP3	1.099951016	0.001167851
SERPINB4	1.099673225	0.046581002
HABP4	1.098701543	8.86E-12
TCF19	1.097101022	0.016917796
ATP8B2	1.093047254	0.000109158
ZNF211	1.092581173	3.71E-06
AC096887.2	1.091745766	0.001665408
AL117336.3	1.091620471	0.011707427
DENND5B-AS1	1.088168764	0.045056584
GUCY1A2	1.086181477	0.002026385
ZNF615	1.08484987	0.023516535
WDFY3-AS2	1.084681686	0.018711772
CRISPLD2	1.083983302	1.06E-10
TVP23A	1.083940194	7.14E-08
ADAMTS5	1.079641048	1.64E-05
HIST1H3D	1.079337629	0.031551977
HIST2H2BE	1.079097982	9.35E-09
DLEU2	1.078886652	9.16E-05
SYT1	1.077989924	0.00624798
UBE2D1	1.075395494	4.32E-06
CXCL3	1.07491411	5.34E-05
ZNF165	1.071999986	4.98E-06
ADTRP	1.068076726	0.020418043
ZFP37	1.067329701	0.00259221
FLT3LG	1.067312552	0.0202049
SERPINB3	1.066098329	0.005797288
PSMB8-AS1	1.065115828	0.00038056
KALRN	1.064910032	0.03365974
AL133346.1	1.063009	3.76E-05
LCORL	1.062361095	2.54E-08
C14orf28	1.060808924	0.001145766
AC037198.1	1.060779683	0.037932964
PLCL1	1.057917738	0.021997075
KCNH1	1.055865739	0.036996619
CTAGE7P	1.054975882	0.03673163
RSPO3	1.05497262	2.12E-08
GPR183	1.054288027	0.022789551
BIRC7	1.052405824	0.023779076
MYBL1	1.052334714	1.13E-06
VIL1	1.046347939	5.66E-07
MED11	1.044621694	2.36E-09
AL513550.1	1.043370686	0.001991086

DLG2	1.04297068	0.030500077
AL606489.1	1.041653279	0.032815775
ZNF17	1.039259657	0.012380174
DDIT3	1.03582164	5.38E-05
KRBA2	1.034046505	0.01622818
ZNF675	1.032016291	0.000170222
SLC7A8	1.031784901	2.61E-08
AL121944.1	1.028302598	0.005186491
AC241585.1	1.027564905	0.036450122
ZNF26	1.027301579	1.12E-09
ZNF45	1.026054309	6.29E-05
SPOPL	1.025271639	5.92E-10
FUT4	1.024809413	0.001847865
FNDC10	1.023694439	3.01E-11
IL6	1.020878634	2.56E-15
ATF1	1.020642274	4.06E-14
BOLA3-AS1	1.020547257	0.004228794
ZMYND10	1.019395497	0.042803824
BHLHE41	1.017724241	0.000580742
LATS2	1.017368671	2.11E-16
MYO15B	1.015606744	0.000281117
B4GALT1	1.012748347	7.42E-24
GPR143	1.012150819	0.034141984
AC092295.2	1.010428012	0.002506667
GRIN1	1.009725399	0.011220199
PRR16	1.008937878	0.007838588
UBE2Q2P1	1.007787684	0.04753924
PDE9A	1.006754985	2.51E-05
CREBRF	1.006535771	9.20E-06
GPAT3	1.006346564	0.002735277
ZNF227	1.006302496	9.41E-06
ZNF484	1.005528933	3.82E-05
POMK	1.005353047	0.001511732
ZNF257	1.00353524	0.002257422
ZNF84	1.002724404	0.000313815
ETAA1	1.00140493	5.96E-06
ZBED3-AS1	-1.00057892	0.02896187
ALDH3A2	-1.002735103	2.01E-06
GCGR	-1.003171295	0.028725641
HPD	-1.005129768	0.000155073
ST6GALNAC6	-1.005381844	0.000133636
MAB21L3	-1.005464978	0.019265607
ADAM9	-1.006447458	3.15E-11
SLC1A3	-1.006915051	0.00024256
MAN2A1	-1.007282047	7.11E-13
MYEOV	-1.009194006	0.003261402
DMBT1	-1.009698129	0.017234824

AC010733.2	-1.010982079	0.008134817
EVI2B	-1.013072401	0.01745221
AC121761.1	-1.019708972	0.003267859
AC104447.1	-1.02011369	0.002932559
ZNF296	-1.020776843	0.000450145
NOL4L	-1.022897748	3.40E-11
IQCH	-1.023002394	0.00598031
NEK6	-1.025640131	1.69E-15
CCND1	-1.028347749	4.96E-26
AC017083.4	-1.030781976	0.000293786
TSSK5P	-1.032990867	0.015999312
MAMDC2	-1.033744125	0.014734697
AVIL	-1.03433244	0.022548774
AC245041.1	-1.036925684	5.45E-07
CCDC51	-1.037245318	1.82E-15
DDX60	-1.039102032	2.75E-08
AC127496.7	-1.040221101	0.026010523
AL513302.2	-1.043397844	0.005124862
ADGRG3	-1.04548658	0.000328399
RN7SL1	-1.049220257	0.031483685
CYSRT1	-1.051156901	4.40E-07
LINC01679	-1.053944194	0.025276975
NSF	-1.055875217	3.17E-13
AL513497.1	-1.05607642	0.029570166
AL132655.2	-1.05775716	0.037762513
NOL9	-1.058661232	2.27E-13
RIPOR2	-1.061157529	0.006447056
ABCC3	-1.065292318	8.04E-25
SUSD2	-1.066281867	3.44E-06
OASL	-1.069351752	3.49E-21
PRSS36	-1.070248947	0.016664167
TNS4	-1.073552161	2.87E-27
CCL5	-1.076162343	1.24E-05
NNAT	-1.076412087	0.037230027
USP32P3	-1.076965617	0.00286924
AC104825.2	-1.078244346	0.047785838
SRD5A3-AS1	-1.084238322	0.043828127
ANKRD34A	-1.084437519	0.000103671
TINAGL1	-1.08669291	3.62E-19
INPP4A	-1.086918236	2.92E-15
FRGCA	-1.087861293	0.003348928
C1orf116	-1.08838302	0.04112067
TJP3	-1.089248304	5.05E-06
LIMS1	-1.092238266	0.01336841
LINC01128	-1.092728972	0.005007948
UBASH3B	-1.094535001	7.14E-12
INHBB	-1.095165185	0.017128193

MMRN2	-1.102315218	0.023683926
ARMC10	-1.103017831	2.93E-06
FAM89A	-1.108644483	0.040564535
AC097461.1	-1.112720722	0.014706417
GXYLT2	-1.120448028	0.014697769
MLXIPL	-1.120732421	0.000287074
VLDLR	-1.123285546	8.17E-14
ACE	-1.12426016	0.016240069
GALNT4	-1.124747359	0.000197727
AC005586.2	-1.130892841	0.000345961
CYP1A1	-1.139893609	4.25E-06
SNX18P7	-1.141031227	0.005864268
NANOS1	-1.141184966	0.00130281
LRGUK	-1.141239483	0.008558985
PITX3	-1.146490251	0.001155976
CFAP54	-1.15314305	0.009851738
SPDYE3	-1.153331651	0.000489431
DAPP1	-1.164790065	0.014950133
CALM2P2	-1.168807357	0.046623837
RHPN1	-1.169741277	4.07E-06
AL451042.2	-1.178946196	0.019291892
AP002387.1	-1.179524222	0.039689529
SAPCD1	-1.180814478	0.013397348
AC092134.1	-1.180942672	0.017346
P2RY11	-1.182902319	0.004346094
AC010442.2	-1.183034577	0.048758846
RARRES3	-1.185459982	1.16E-12
AL096870.2	-1.185933544	0.019556857
UGT1A7	-1.186404401	4.46E-06
EPHB4	-1.186845218	1.56E-25
AC105285.1	-1.187926704	0.000141936
SAMD8	-1.189347505	8.18E-06
AL603750.1	-1.19215682	0.043876983
AC093525.6	-1.193280705	0.004057266
PPM1J	-1.196782265	0.031165521
CACNG6	-1.197325775	0.042871825
AC040162.1	-1.198909074	0.007244641
SOCS1	-1.199197282	0.016120617
PXK	-1.200247169	4.64E-08
AC084876.1	-1.201139422	0.019699401
CMTM4	-1.202178522	3.04E-26
LINC01759	-1.202881411	0.006809553
PECAM1	-1.202946518	0.043498255
LINC00570	-1.208250909	0.021043444
PPM1E	-1.214038579	1.28E-06
POLN	-1.215940696	0.013707417
TMEM63C	-1.221430701	0.039035663

SERPINB7	-1.223393457	0.000816679
KDM1B	-1.225511555	1.73E-11
ATP2A2	-1.234884743	9.58E-28
AC020978.10	-1.235043779	0.000451773
AC005034.3	-1.23600705	2.16E-08
KLHL30	-1.238397911	1.95E-05
AP003469.4	-1.254026674	0.038691171
LMO4	-1.256830616	2.14E-29
FLJ31104	-1.260467287	0.033888767
AC234582.1	-1.261382681	0.000512027
SRMS	-1.263569775	0.016358845
ASNS	-1.280246327	4.31E-11
GRIN3B	-1.281170602	0.049964201
SLC12A3	-1.288284473	5.83E-30
GACAT2	-1.28871477	0.009728349
HMGA2	-1.290891605	0.000514793
RNA5-8SN4	-1.293617777	0.001769115
EXOC3L4	-1.293653732	0.002242517
HTR1D	-1.295241885	2.23E-15
FBXO36	-1.296321621	0.00071956
HSPB6	-1.300215406	0.018280787
AC080038.3	-1.308173972	0.004465671
AKR1B15	-1.308363136	0.008449675
FAM71E1	-1.309614082	0.031790219
RNF38	-1.313306777	6.65E-14
BTBD19	-1.314102118	0.001426094
FAM86C2P	-1.316284584	1.46E-06
SEMA3G	-1.316316083	0.002399885
SSC4D	-1.319834433	2.16E-06
RNA5-8SN1	-1.320247654	0.013469732
ASPHD2	-1.32337242	0.041858942
NONOP2	-1.324111108	0.022052225
AC005034.5	-1.328282054	0.041715219
CLDN4	-1.329585754	1.36E-05
HCG25	-1.331743154	0.004074904
AL358790.1	-1.33208541	0.008968688
ZHX1-C8orf76	-1.33537967	0.014331428
CLIC3	-1.338669221	0.000102774
GARNL3	-1.342869354	0.002496337
RNA5-8SN5	-1.352691179	0.000744958
ENTPD7	-1.354387529	8.94E-16
RSPH4A	-1.356023672	0.041653337
VWCE	-1.361135367	0.012077378
A2M	-1.362042606	0.040981444
C20orf196	-1.363656968	0.006544525
CCNG1	-1.365895609	6.77E-11
PRELID2	-1.369922352	1.53E-10

PHOSPHO1	-1.372654047	0.037973347
BASP1	-1.37463577	8.15E-46
SIRT4	-1.381855537	0.000417659
TRIM22	-1.384012812	0.011187451
DNAAF3	-1.387823119	6.82E-05
AC005324.5	-1.394012844	6.73E-07
SLC12A5	-1.39445413	0.023401268
DDAH1	-1.399598463	8.97E-28
AC006262.2	-1.401545997	0.012310846
MIR22HG	-1.407500564	8.00E-08
AC089983.1	-1.410649484	5.64E-10
LINC00920	-1.42473044	0.000549714
CCL22	-1.432483233	0.03693134
DGCR9	-1.444036664	0.019578729
CLDN9	-1.450126492	0.031903288
AL023284.4	-1.451301865	0.032253958
AC004520.1	-1.453619357	0.018986449
AC090510.1	-1.469820568	0.041105238
AKR1C1	-1.472637331	2.83E-29
GGT5	-1.481429606	0.001235884
MERTK	-1.495289402	0.032502892
GSTM2	-1.495733238	0.007489138
AL035413.2	-1.497711285	0.033517043
AC139795.2	-1.498545479	0.002264119
GFM2	-1.500484289	2.97E-37
GSDMB	-1.504510059	7.70E-05
AP005264.1	-1.505918784	0.032028321
SNHG17	-1.506402759	5.08E-22
TPP1	-1.5082058	2.96E-34
BORCS8-MEF2B	-1.514190937	0.000240196
TOMM6	-1.538073071	5.30E-08
GBP4	-1.559034661	0.006857229
LINC01970	-1.580897245	0.011420628
G6PD	-1.582887916	3.55E-45
TMEM52	-1.588692021	0.024541138
AC018629.1	-1.597983196	1.95E-06
SLC25A23	-1.608210875	1.84E-27
DDIAS	-1.612551323	5.52E-08
EFCAB12	-1.612814495	0.042093057
STMN3	-1.616341947	0.003650373
AC097662.1	-1.61955443	0.049568464
ACE2	-1.620129709	0.002325772
PHC1	-1.622473501	0.005154436
GLI1	-1.63798015	0.000639198
TMEM171	-1.652436269	0.010432719
TMIE	-1.652895346	0.017988654
AC116366.1	-1.652952152	0.003384858

AC105052.1	-1.665763343	0.012569028
FLJ22447	-1.675029234	0.003291363
EXTL3-AS1	-1.681221072	0.014431227
NDUFC2-KCTD14	-1.682751649	0.000456864
CARNMT1	-1.685103847	1.00E-19
SCO2	-1.68658873	0.002874421
AC007114.1	-1.692125502	0.014562017
AC007161.3	-1.697997287	0.000542252
SLC4A8	-1.701511523	0.028968551
SOCS2-AS1	-1.707251452	0.032457925
HIST1H4C	-1.710904858	0.017008378
AMY2B	-1.713811616	0.000779145
CLSTN2	-1.71405695	0.003184912
CDH1	-1.723882834	0.02322824
KRT87P	-1.73074135	9.68E-07
KCNH2	-1.735654532	0.019821675
RASA4CP	-1.759490285	0.010431505
AC007238.1	-1.767597731	0.041048863
AL669918.1	-1.769750487	0.012587226
NFAM1	-1.769830751	0.019020727
LINC02361	-1.783667081	0.019230587
ACSM5	-1.79102745	0.029495274
PCAT6	-1.816623112	0.002464398
CD7	-1.820453151	0.001663175
HNRNPA1P49	-1.826974008	0.020668662
C1orf220	-1.83187848	0.002314371
AP002336.2	-1.839563643	0.044352921
KLRG1	-1.848146619	0.002150762
ANKRD2	-1.861036726	3.19E-09
CTRB2	-1.866407933	0.003735817
AC020978.8	-1.900872172	0.000104735
AL355802.3	-1.912196531	0.000149913
AC017074.1	-1.924784577	0.023469849
ZNF571-AS1	-1.935717011	0.044945711
AKR1C2	-1.959465688	3.09E-12
AC007192.2	-1.974790846	0.002581221
NHLH1	-1.984744889	0.003360532
MILR1	-1.993425578	0.006952929
LCTL	-2.00573627	0.003414172
ERH	-2.024561071	7.92E-65
AC022400.3	-2.081932951	0.004125967
HOXA2	-2.092491169	0.021725992
APOBR	-2.099172089	0.000250715
AC023043.2	-2.113239603	0.00906188
AL359922.1	-2.117052039	0.003568544
PLA2G4B	-2.131986127	0.012570372
RN7SK	-2.144264498	1.20E-12

RRAD	-2.157401032	0.043187134
C1orf228	-2.159640207	0.003609144
MIR31HG	-2.166931813	1.35E-11
AC087392.3	-2.180271186	0.014671864
ROBO1	-2.18778612	2.28E-43
AP001148.1	-2.210380372	0.005435903
AC013643.3	-2.215377967	0.044482704
TBCA	-2.278903462	5.86E-63
AL390066.1	-2.314365604	0.009330169
AC006486.1	-2.320507359	0.02748444
AVPR2	-2.382239471	0.024360784
TTC28-AS1_2	-2.386488201	0.007195789
KCNK9	-2.405495843	0.045288257
CA14	-2.42835676	0.029794111
KCTD19	-2.428396147	0.041617137
CPA2	-2.489530306	0.031707044
AP000777.3	-2.494206648	0.024355811
IFNWP19	-2.520001934	2.12E-15
AP001160.2	-2.538017074	0.010866602
OXTR	-2.556174291	0.017626559
AC080162.1	-2.572098412	0.030646197
GABRR2	-2.5797233	0.022083863
LGALS9B	-2.583676573	2.71E-05
FER1L4	-2.606241999	1.16E-05
CFAP43	-2.608362139	0.010791925
AC012377.1	-2.635495571	0.028236395
AC100827.3	-2.656904457	0.000265646
GJA5	-2.658051859	0.007413743
CLCA2	-2.662540161	0.009930451
ALG1L7P	-2.663451991	0.01811064
ELOCP19	-2.685888623	0.046194751
AL161937.2	-2.713405155	0.0253121
BCRP7	-2.737786513	0.027005132
LINC01219	-2.748967484	0.00385038
CYP4F3	-2.808597682	0.005682226
MIR3074	-2.865275456	0.022404475
TUBA8	-2.893850561	9.62E-06
AC011445.1	-2.90757466	1.52E-08
AC073610.1	-3.022545271	0.014052893
LINC02280	-3.074809439	0.03068403
SNORD22	-3.095586566	0.046299783
TSPAN6	-3.102142386	1.42E-46
ATP6V1C2	-3.105482626	0.00204579
USP12-AS2	-3.106091854	0.048610949
RPS20P21	-3.18012928	0.019632036
MIR34AHG	-3.185688469	0.046853273
AC084337.2	-3.320133294	0.028446976

AL691442.1	-3.38169834	0.001332749
C1QTNF4	-3.401391849	0.028706096
SLC23A3	-3.4581885	0.020597993
MIR137	-3.477228854	0.014315351
KRT8P45	-3.493483114	0.020027504
AC027307.3	-3.511779925	0.020823604
AC087632.1	-3.535503603	1.94E-14
CAPN3	-3.58121113	0.013491435
CD226	-3.581628905	0.009149055
AC020907.4	-3.663860362	0.019702859
Z98884.2	-3.6638898	0.031503218
CYP4F11	-3.679679651	6.32E-05
PKN2-AS1	-3.693780377	0.005988314
AC020634.1	-3.695125265	0.018262475
AL512274.1	-3.761588774	0.020283877
FGF7P3	-3.799381406	7.43E-05
PKP4-AS1	-3.85687297	0.00850322
MICD	-3.89794832	0.045372734
SLC47A1P1	-3.924683013	0.032051987
AC141557.1	-4.100399699	0.044273105
AC126182.2	-4.13396809	0.049917746
LINC01376	-4.205637832	0.002757828
HIST1H3J	-4.212581994	0.00021516
AC090695.1	-4.268481545	0.009705529
AC073487.1	-4.340466396	0.042216146
UBE2L5P	-4.346864852	0.028453452
AC005041.1	-4.381539103	0.01030345
AC067968.1	-4.493268786	0.001487304
RFPL3S	-4.725609473	0.01436918
PCDHGA12	-4.731303017	0.049727326
AL583722.4	-4.731303017	0.049727326
AC008013.1	-4.761534843	0.047411841
TAS2R19	-4.761810954	0.047204721
NOS2P3	-4.761810954	0.047204721
AL627389.1	-4.805183303	0.046706654
TCTE1	-4.83454448	0.048996903
AL353719.1	-4.837713004	0.038963087
AC004461.2	-4.851496753	0.042020429
GKN2	-4.851985656	0.03949223
CYCSP10	-4.861730395	0.001096847
RPSAP13	-4.915622905	0.038079134
ZFP57	-4.916622885	0.032598376
ABCA9	-4.926631067	0.031814009
AC007272.1	-4.926631067	0.031814009
AC064807.2	-4.926631067	0.031814009
ERVMER34-1	-4.968770834	0.032379721
ALG1L2	-4.984752246	0.027188597

AL583810.1	-4.995563603	0.008654663
AL138995.1	-5.017024647	0.028070169
DCDC1	-5.040759501	0.026833312
LMO2	-5.044002259	0.026144752
AC011498.5	-5.052994372	0.02360884
TXNP6	-5.062113036	0.022393645
AL121894.2	-5.062560396	0.023170337
SNORA18	-5.063479972	0.001986257
AC026954.2	-5.074628124	0.010970448
AP001266.2	-5.07477395	0.022622313
AC005618.1	-5.077466311	0.021723256
GYG2	-5.092972313	0.023793249
AP001025.1	-5.109266182	0.024203235
APOBEC3A	-5.135966633	0.018211724
LINC00221	-5.136179839	0.019252501
AC118658.2	-5.16477425	0.048622837
ARL11	-5.165127844	0.020308208
KRTAP5-2	-5.168149243	0.021593214
ANO5	-5.168336232	0.047819083
AL355922.3	-5.176863175	0.047970697
AC073326.1	-5.18228178	0.047079817
C3orf20	-5.205406674	0.016593799
AC046134.1	-5.207487653	0.029670805
AC063944.1	-5.222597014	0.015446754
AL162456.1	-5.229448811	0.046649288
AL590302.2	-5.236716333	0.041841998
PRG2	-5.238185827	0.001969586
SCG3	-5.240420496	0.044841689
AC127496.5	-5.2585562	0.017907103
AC132938.5	-5.258790736	0.015510342
WDR72	-5.27007836	0.012404451
PRR35	-5.289143615	0.038567245
P2RX6P	-5.313752535	0.012068106
MIRLET7I	-5.328775794	0.010424884
PSAPL1	-5.331414333	0.010288561
AC087385.1	-5.362002692	0.033170744
AL158835.3	-5.362002692	0.033170744
ITGA11	-5.370537781	0.010278734
AC010503.2	-5.373514964	0.032580602
AL158071.4	-5.383343913	0.009998654
AL121839.2	-5.416202458	0.01040272
AC107067.1	-5.422342303	0.011186768
KLHDC1	-5.433684308	0.031313736
LINC00441	-5.442485889	0.032159101
AC244033.1	-5.449563455	0.007752092
CORO2B	-5.465227133	0.027696199
OR1F12	-5.512956238	0.024213533

TMX2-CTNND1	-5.513771105	0.026741548
SCTR	-5.528960232	0.008385624
ST8SIA6-AS1	-5.545660839	0.026425438
AL031731.1	-5.555540941	0.005755488
AC241644.3	-5.586130336	0.021490899
TXNRD3NB	-5.594425704	0.020154924
AC007663.1	-5.614151824	0.007005918
AC099336.1	-5.625403989	0.018631834
PRAP1	-5.645717623	0.022817023
MGARP	-5.677620575	0.016921512
FCN2	-5.689303784	0.018887057
AC138356.1	-5.717767158	0.004060481
NPC1L1	-5.725700286	0.004334137
TTLL13P	-5.754925987	0.017445537
MYO16-AS1	-5.759506778	0.002959992
AL365226.1	-5.769062473	0.004320725
AC244034.3	-5.808683279	0.012727841
PSPC1P1	-5.811678816	0.038703581
AC018616.1	-5.838940845	0.002033177
AC105339.2	-5.887706321	0.00990506
CELF6	-5.90081513	0.002713874
AGAP11	-5.903505686	0.001878873
AL358075.4	-5.932329002	0.016343631
AC040977.2	-5.938770635	0.031429361
C1orf127	-5.949493363	0.00116072
LINC01776	-5.982317759	0.007979737
SOCS5P4	-5.986183542	0.000982259
AC091100.1	-6.03625251	0.006693387
UBASH3A	-6.040382562	0.001252443
LRRC32	-6.049424518	0.001188898
LINC01979	-6.256732681	0.000298783
FBXO15	-6.259869392	0.000498043
AC008734.1	-6.299828334	0.000220047
AL109955.1	-6.325829637	0.000276434
AC092159.2	-6.386541522	0.000144218
AL355987.3	-6.406488001	1.07E-05
DAB1	-6.410107056	0.012746148
F2RL2	-6.423444101	0.000220799
AL683813.1	-6.451225861	0.000154939
AC233702.10	-6.502204361	0.001821716
AL139300.1	-6.515040698	0.010266159
ADAM32	-6.692552804	4.47E-05
AC114755.5	-6.707682068	2.80E-05
AS3MT	-8.577988993	7.11E-11
ATP6V1G2-DDX39B	-9.219874354	0.012161272
AL158151.1	-12.231728	1.44E-24
TNXA	-22.06287811	1.65E-08

AC027682.3	-22.86762953	4.86E-09
FP565260.2	-22.96935839	4.15E-09
AC092849.1	-23.98447524	8.33E-10
DNAAF4-CCPG1	-24.64244874	2.84E-10
