# Supplementary material for: LncRNA SNHG17 Contributes to Proliferation, Migration, and Poor Prognosis of Hepatocellular Carcinoma
Source: Can J Gastroenterol Hepatol. 2021 Sep 14;2021:9990338. doi: 10.1155/2021/9990338 (PMC8455207; doi:10.1155/2021/9990338)
Supplement: Supplementary Materials — Supplementary figure legends: Figure S1. Unstained-isotype control of Hep3B (A) and SMMC-7721 (B). Figure S2. SNHG17 promoted cell invasion of HCC. (A) The representative images of transwell assay in HuH-7 cell (magnification: 100X). (B) Quantitative data of transwell results in HuH-7 cells. ∗∗∗P < 0.001. Figure S3. Distribution of KEGG terms for 1037 genes altered (≥2-fold change, P < 0.05) after knockdown of SNHG17 in Hep3B cells. Figure S4. Distribution of GO terms, including molecular function, biological process, and cellular component, for 1037 genes altered (≥2-fold change, P < 0.05) after knockdown of SNHG17 in Hep3B cells. Figure S5. The expression levels of ERH (A) and TBCA (B) in the TCGA-LIHC and GSE102079 HCC dataset. Figure S6. The expression levels of TDO2 (A) and PDK4 (B) in the TCGA-LIHC and GSE102079 HCC dataset. Figure S7. Univariate and multivariate Cox regression analyses of SNHG17 expression in HCC regarding overall survival. Figure S8. Univariate and multivariate Cox regression analyses of ERH expression in HCC regarding overall survival. Figure S9. Univariate and multivariate Cox regression analyses of PDK4 expression in HCC regarding overall survival. Supplementary tables: Table S1. The 1037 genes altered (≥2-fold change, P < 0.05) after knockdown of SNHG17 in Hep3B cells, with three repeats by RNA sequencing. Table S2. The list of KEGG terms for 1037 genes altered (≥2-fold change, P < 0.05) after knockdown of SNHG17 in Hep3B cells. Table S3. The list of GO terms for 1037 genes altered (≥2-fold change, P < 0.05) after knockdown of SNHG17 in Hep3B cells. Table S4. The overlap of SNHG17-related genes in RNA-sequencing results and HCC tissues (TCGA-LIHC). [file 9990338.f1.zip › 9990338.f1/Table S2 (2).pdf]

Term	Count	Ratio	Corrected_P_Value
Metabolic pathways	45	0.031402652	1.14E-05
Herpes simplex virus 1 infection	38	0.077235772	2.26E-14
Neuroactive ligand-receptor interaction	15	0.044378698	0.002798878
Cytokine-cytokine receptor interaction	14	0.047619048	0.002473245
PI3K-Akt signaling pathway	14	0.039548023	0.009534175
Cell adhesion molecules (CAMs)	12	0.082191781	0.000113287
Transcriptional misregulation in cancer	12	0.064516129	0.000781924
MicroRNAs in cancer	12	0.040133779	0.018006855
Human papillomavirus infection	12	0.036363636	0.032918595
cGMP-PKG signaling pathway	10	0.05988024	0.004457159
Alcoholism	10	0.055555556	0.006816266
Oxytocin signaling pathway	9	0.058823529	0.008790115
Hypertrophic cardiomyopathy (HCM)	8	0.088888889	0.002175118
Systemic lupus erythematosus	8	0.060150376	0.012898088
Jak-STAT signaling pathway	8	0.049382716	0.033020421
Rheumatoid arthritis	7	0.076923077	0.009102869
Prostate cancer	7	0.072164948	0.011057824
Hematopoietic cell lineage	7	0.072164948	0.011057824
Apelin signaling pathway	7	0.051094891	0.049265375
p53 signaling pathway	6	0.083333333	0.013565295
