# Supplementary material for: LncRNA SNHG17 Contributes to Proliferation, Migration, and Poor Prognosis of Hepatocellular Carcinoma
Source: Can J Gastroenterol Hepatol. 2021 Sep 14;2021:9990338. doi: 10.1155/2021/9990338 (PMC8455207; doi:10.1155/2021/9990338)
Supplement: Supplementary Materials — Supplementary figure legends: Figure S1. Unstained-isotype control of Hep3B (A) and SMMC-7721 (B). Figure S2. SNHG17 promoted cell invasion of HCC. (A) The representative images of transwell assay in HuH-7 cell (magnification: 100X). (B) Quantitative data of transwell results in HuH-7 cells. ∗∗∗P < 0.001. Figure S3. Distribution of KEGG terms for 1037 genes altered (≥2-fold change, P < 0.05) after knockdown of SNHG17 in Hep3B cells. Figure S4. Distribution of GO terms, including molecular function, biological process, and cellular component, for 1037 genes altered (≥2-fold change, P < 0.05) after knockdown of SNHG17 in Hep3B cells. Figure S5. The expression levels of ERH (A) and TBCA (B) in the TCGA-LIHC and GSE102079 HCC dataset. Figure S6. The expression levels of TDO2 (A) and PDK4 (B) in the TCGA-LIHC and GSE102079 HCC dataset. Figure S7. Univariate and multivariate Cox regression analyses of SNHG17 expression in HCC regarding overall survival. Figure S8. Univariate and multivariate Cox regression analyses of ERH expression in HCC regarding overall survival. Figure S9. Univariate and multivariate Cox regression analyses of PDK4 expression in HCC regarding overall survival. Supplementary tables: Table S1. The 1037 genes altered (≥2-fold change, P < 0.05) after knockdown of SNHG17 in Hep3B cells, with three repeats by RNA sequencing. Table S2. The list of KEGG terms for 1037 genes altered (≥2-fold change, P < 0.05) after knockdown of SNHG17 in Hep3B cells. Table S3. The list of GO terms for 1037 genes altered (≥2-fold change, P < 0.05) after knockdown of SNHG17 in Hep3B cells. Table S4. The overlap of SNHG17-related genes in RNA-sequencing results and HCC tissues (TCGA-LIHC). [file 9990338.f1.zip › 9990338.f1/Table S3 (2).pdf]

Category	Term	Count	PValue
GOTERM_MF_DIRECT	metal ion binding	92	9.05E-05
GOTERM_MF_DIRECT	DNA binding	63	0.045323263
GOTERM_MF_DIRECT	zinc ion binding	46	0.049080945
GOTERM_MF_DIRECT	nucleic acid binding	43	0.013086382
GOTERM_MF_DIRECT	transcription factor activity, sequence-specific DNA binding	41	0.022166909
GOTERM_MF_DIRECT	protein homodimerization activity	32	0.032691732
GOTERM_MF_DIRECT	RNA polymerase II core promoter proximal region sequence-specific DNA binding	22	0.002427141
GOTERM_MF_DIRECT	virus receptor activity	8	0.004850987
GOTERM_MF_DIRECT	transcriptional repressor activity, RNA polymerase II core promoter proximal region sequence-specific binding	8	0.049030087
GOTERM_MF_DIRECT	metallopeptidase activity	7	0.034314727
GOTERM_CC_DIRECT	extracellular region	70	0.0013419
GOTERM_CC_DIRECT	extracellular space	68	2.32E-05
GOTERM_CC_DIRECT	intracellular	59	0.002283486
GOTERM_CC_DIRECT	integral component of plasma membrane	57	0.017489128
GOTERM_CC_DIRECT	cell surface	29	0.00353868
GOTERM_CC_DIRECT	external side of plasma membrane	14	0.011676679
GOTERM_CC_DIRECT	postsynaptic density	13	0.009247856
GOTERM_CC_DIRECT	nucleosome	8	0.022180842
GOTERM_CC_DIRECT	brush border	6	0.032906234
GOTERM_BP_DIRECT	transcription, DNA-templated	76	0.018342059
GOTERM_BP_DIRECT	cell adhesion	23	0.023547693
GOTERM_BP_DIRECT	response to drug	19	0.005453425
GOTERM_BP_DIRECT	cell surface receptor signaling pathway	16	0.020360037
GOTERM_BP_DIRECT	regulation of cell proliferation	13	0.01089654
GOTERM_BP_DIRECT	platelet degranulation	9	0.013012244
GOTERM_BP_DIRECT	response to endoplasmic reticulum stress	8	0.007580686
GOTERM_BP_DIRECT	viral entry into host cell	8	0.010671725
GOTERM_BP_DIRECT	epithelial cell differentiation	7	0.019256613
GOTERM_BP_DIRECT	positive regulation of apoptotic signaling pathway	4	0.0388358
