# Supplementary material for: LncRNA SNHG17 Contributes to Proliferation, Migration, and Poor Prognosis of Hepatocellular Carcinoma
Source: Can J Gastroenterol Hepatol. 2021 Sep 14;2021:9990338. doi: 10.1155/2021/9990338 (PMC8455207; doi:10.1155/2021/9990338)
Supplement: Supplementary Materials — Supplementary figure legends: Figure S1. Unstained-isotype control of Hep3B (A) and SMMC-7721 (B). Figure S2. SNHG17 promoted cell invasion of HCC. (A) The representative images of transwell assay in HuH-7 cell (magnification: 100X). (B) Quantitative data of transwell results in HuH-7 cells. ∗∗∗P < 0.001. Figure S3. Distribution of KEGG terms for 1037 genes altered (≥2-fold change, P < 0.05) after knockdown of SNHG17 in Hep3B cells. Figure S4. Distribution of GO terms, including molecular function, biological process, and cellular component, for 1037 genes altered (≥2-fold change, P < 0.05) after knockdown of SNHG17 in Hep3B cells. Figure S5. The expression levels of ERH (A) and TBCA (B) in the TCGA-LIHC and GSE102079 HCC dataset. Figure S6. The expression levels of TDO2 (A) and PDK4 (B) in the TCGA-LIHC and GSE102079 HCC dataset. Figure S7. Univariate and multivariate Cox regression analyses of SNHG17 expression in HCC regarding overall survival. Figure S8. Univariate and multivariate Cox regression analyses of ERH expression in HCC regarding overall survival. Figure S9. Univariate and multivariate Cox regression analyses of PDK4 expression in HCC regarding overall survival. Supplementary tables: Table S1. The 1037 genes altered (≥2-fold change, P < 0.05) after knockdown of SNHG17 in Hep3B cells, with three repeats by RNA sequencing. Table S2. The list of KEGG terms for 1037 genes altered (≥2-fold change, P < 0.05) after knockdown of SNHG17 in Hep3B cells. Table S3. The list of GO terms for 1037 genes altered (≥2-fold change, P < 0.05) after knockdown of SNHG17 in Hep3B cells. Table S4. The overlap of SNHG17-related genes in RNA-sequencing results and HCC tissues (TCGA-LIHC). [file 9990338.f1.zip › 9990338.f1/Table S4 (2).pdf]

SNHG17 positive-related genes in TCGA	Downregulated genes in RNA-seq data	Overlap
ZYX	ZBED3-AS1	ADAM9
ZXDC	ALDH3A2	DMBT1
ZWINT	GCGR	ZNF296
ZWILCH	HPD	NOL4L
ZW10	ST6GALNAC6	NEK6
ZUP1	MAB21L3	CCDC51
ZSWIM9	ADAM9	CYSRT1
ZSWIM7	SLC1A3	NSF
ZSWIM5	MAN2A1	NOL9
ZSWIM4	MYEOV	ANKRD34A
ZSWIM3	DMBT1	INPP4A
ZSWIM1	AC010733.2	C1orf116
ZSCAN9	EVI2B	LIMS1
ZSCAN5C	AC121761.1	UBASH3B
ZSCAN5A	AC104447.1	ARMC10
ZSCAN31	ZNF296	FAM89A
ZSCAN30	NOL4L	GXYLT2
ZSCAN29	IQCH	ACE
ZSCAN26	NEK6	NANOS1
ZSCAN25	CCND1	LRGUK
ZSCAN22	AC017083.4	PITX3
ZSCAN20	TSSK5P	SPDYE3
ZSCAN2	MAMDC2	RHPN1
ZSCAN16	AVIL	SAPCD1
ZSCAN12	AC245041.1	P2RY11
ZRSR2	CCDC51	SOCS1
ZRANB2	DDX60	CMTM4
ZPR1	AC127496.7	TMEM63C
ZP3	AL513302.2	ASNS
ZP1	ADGRG3	GRIN3B
ZNRF1	RN7SL1	HMGA2
ZNRD2	CYSRT1	HTR1D
ZNRD1	LINC01679	FAM71E1
ZNHIT6	NSF	SSC4D
ZNHIT3	AL513497.1	ASPHD2
ZNHIT2	AL132655.2	ZHX1-C8orf76
ZNHIT1	NOL9	CLIC3
ZNF93	RIPOR2	RSPH4A
ZNF92	ABCC3	PRELID2
ZNF91	SUSD2	SIRT4
ZNF90	OASL	SLC12A5
ZNF891	PRSS36	GSDMB

ZNF888
ZNF880
ZNF878
ZNF875
ZNF865
ZNF862
ZNF853
ZNF852
ZNF850
ZNF85
ZNF846
ZNF841
ZNF84
ZNF836
ZNF830
ZNF83
ZNF823
ZNF821
ZNF814
ZNF813
ZNF8
ZNF799
ZNF793
ZNF792
ZNF791
ZNF79
ZNF789
ZNF787
ZNF786
ZNF785
ZNF784
ZNF783
ZNF782
ZNF780B
ZNF780A
ZNF778
ZNF777
ZNF775
ZNF774
ZNF773
ZNF771
ZNF77
ZNF768
ZNF766

TNS4
CCL5
NNAT
USP32P3
AC104825.2
SRD5A3-AS1
ANKRD34A
TINAGL1
INPP4A
FRGCA
C1orf116
TJP3
LIMS1
LINC01128
UBASH3B
INHBB
MMRN2
ARMC10
FAM89A
AC097461.1
GXYLT2
MLXIPL
VLDLR
ACE
GALNT4
AC005586.2
CYP1A1
SNX18P7
NANOS1
LRGUK
PITX3
CFAP54
SPDYE3
DAPP1
CALM2P2
RHPN1
AL451042.2
AP002387.1
SAPCD1
AC092134.1
P2RY11
AC010442.2
RARRES3
AL096870.2

G6PD
DDIAS
STMN3
PHC1
GLI1
SCO2
SLC4A8
KCNH2
CD7
ANKRD2
NHLH1
LCTL
ERH
HOXA2
APOBR
ROBO1
TBCA
KCNK9
KCTD19
CPA2
OXTR
CFAP43
ATP6V1C2
TAS2R19
ERVMER34-1
ALG1L2
SCG3
PRR35
PSAPL1
C1orf127
ADAM32

ZNF765	UGT1A7
ZNF764	EPHB4
ZNF763	AC105285.1
ZNF761; ZNF765-ZNF761	SAMD8
ZNF76	AL603750.1
ZNF75A	AC093525.6
ZNF749	PPM1J
ZNF747	CACNG6
ZNF746	AC040162.1
ZNF740	SOCS1
ZNF74	PXK
ZNF738	AC084876.1
ZNF736	CMTM4
ZNF732	LINC01759
ZNF730	PECAM1
ZNF724	LINC00570
ZNF721	PPM1E
ZNF718	POLN
ZNF714	TMEM63C
ZNF713	SERPINB7
ZNF711	KDM1B
ZNF710	ATP2A2
ZNF71	AC020978.10
ZNF709	AC005034.3
ZNF708	KLHL30
ZNF707	AP003469.4
ZNF706	LMO4
ZNF704	FLJ31104
ZNF703	AC234582.1
ZNF701	SRMS
ZNF700	ASNS
ZNF70	GRIN3B
ZNF7	SLC12A3
ZNF696	GACAT2
ZNF695	HMGA2
ZNF692	RNA5-8SN4
ZNF691	EXOC3L4
ZNF687	HTR1D
ZNF682	FBXO36
ZNF681	HSPB6
ZNF678	AC080038.3
ZNF675	AKR1B15
ZNF672	FAM71E1
ZNF669	RNF38

ZNF668	BTBD19
ZNF665	FAM86C2P
ZNF664	SEMA3G
ZNF660	SSC4D
ZNF66	RNA5-8SN1
ZNF655	ASPHD2
ZNF653	NONOP2
ZNF652	AC005034.5
ZNF646	CLDN4
ZNF641	HCG25
ZNF639	AL358790.1
ZNF638	ZHX1-C8orf76
ZNF629	CLIC3
ZNF628	GARNL3
ZNF627	RNA5-8SN5
ZNF624	ENTPD7
ZNF623	RSPH4A
ZNF622	VWCE
ZNF621	A2M
ZNF620	C20orf196
ZNF618	CCNG1
ZNF614	PRELID2
ZNF613	PHOSPHO1
ZNF610	BASP1
ZNF607	SIRT4
ZNF606	TRIM22
ZNF605	DNAAF3
ZNF600	AC005324.5
ZNF599	SLC12A5
ZNF598	DDAH1
ZNF594	AC006262.2
ZNF593	MIR22HG
ZNF592	AC089983.1
ZNF589	LINC00920
ZNF587B	CCL22
ZNF587	DGCR9
ZNF586	CLDN9
ZNF584	AL023284.4
ZNF581	AC004520.1
ZNF580	AC090510.1
ZNF579	AKR1C1
ZNF577	GGT5
ZNF576	MERTK
ZNF574	GSTM2

ZNF573	AL035413.2
ZNF572	AC139795.2
ZNF571	GFM2
ZNF570	GSDMB
ZNF57	AP005264.1
ZNF567	SNHG17
ZNF566	TPP1
ZNF565	BORCS8-MEF2B
ZNF562	TOMM6
ZNF561	GBP4
ZNF559-ZNF177	LINC01970
ZNF559	G6PD
ZNF557	TMEM52
ZNF556	AC018629.1
ZNF555	SLC25A23
ZNF554	DDIAS
ZNF552	EFCAB12
ZNF551	STMN3
ZNF550	AC097662.1
ZNF548	ACE2
ZNF547	PHC1
ZNF546	GLI1
ZNF544	TMEM171
ZNF543	TMIE
ZNF532	AC116366.1
ZNF530	AC105052.1
ZNF529	FLJ22447
ZNF527	EXTL3-AS1
ZNF526	NDUFC2-KCTD14
ZNF525	CARNMT1
ZNF524	SCO2
ZNF519	AC007114.1
ZNF518A	AC007161.3
ZNF517	SLC4A8
ZNF516	SOCS2-AS1
ZNF514	HIST1H4C
ZNF513	AMY2B
ZNF512B	CLSTN2
ZNF512	CDH1
ZNF511	KRT87P
ZNF507	KCNH2
ZNF506	RASA4CP
ZNF503	AC007238.1
ZNF500	AL669918.1

ZNF496	NFAM1
ZNF488	LINC02361
ZNF487	ACSM5
ZNF485	PCAT6
ZNF480	CD7
ZNF48	HNRNPA1P49
ZNF473	C1orf220
ZNF467	AP002336.2
ZNF461	KLRG1
ZNF451	ANKRD2
ZNF45	CTRB2
ZNF449	AC020978.8
ZNF446	AL355802.3
ZNF445	AC017074.1
ZNF444	ZNF571-AS1
ZNF443	AKR1C2
ZNF442	AC007192.2
ZNF440	NHLH1
ZNF44	MILR1
ZNF439	LCTL
ZNF438	ERH
ZNF436	AC022400.3
ZNF433	HOXA2
ZNF432	APOBR
ZNF431	AC023043.2
ZNF430	AL359922.1
ZNF43	PLA2G4B
ZNF428	RN7SK
ZNF426	RRAD
ZNF420	C1orf228
ZNF419	MIR31HG
ZNF416	AC087392.3
ZNF414	ROBO1
ZNF408	AP001148.1
ZNF404	AC013643.3
ZNF398	TBCA
ZNF397	AL390066.1
ZNF394	AC006486.1
ZNF391	AVPR2
ZNF385C	TTC28-AS1_2
ZNF385A	KCNK9
ZNF383	CA14
ZNF382	KCTD19
ZNF37A	CPA2

ZNF362	AP000777.3
ZNF358	IFNWP19
ZNF354B	AP001160.2
ZNF354A	OXTR
ZNF350	AC080162.1
ZNF35	GABRR2
ZNF346	LGALS9B
ZNF345	FER1L4
ZNF343	CFAP43
ZNF341	AC012377.1
ZNF34	AC100827.3
ZNF33B	GJA5
ZNF33A	CLCA2
ZNF337	ALG1L7P
ZNF335	ELOCP19
ZNF333	AL161937.2
ZNF329	BCRP7
ZNF324B	LINC01219
ZNF324	CYP4F3
ZNF320	MIR3074
ZNF32	TUBA8
ZNF319	AC011445.1
ZNF318	AC073610.1
ZNF317	LINC02280
ZNF316	SNORD22
ZNF304	TSPAN6
ZNF302	ATP6V1C2
ZNF300	USP12-AS2
ZNF30	RPS20P21
ZNF3	MIR34AHG
ZNF296	AC084337.2
ZNF292	AL691442.1
ZNF286B	C1QTNF4
ZNF286A	SLC23A3
ZNF285	MIR137
ZNF284	KRT8P45
ZNF283	AC027307.3
ZNF282	AC087632.1
ZNF280C	CAPN3
ZNF280B	CD226
ZNF280A	AC020907.4
ZNF276	Z98884.2
ZNF273	CYP4F11
ZNF267	PKN2-AS1

ZNF266	AC020634.1
ZNF263	AL512274.1
ZNF260	FGF7P3
ZNF26	PKP4-AS1
ZNF256	MICD
ZNF253	SLC47A1P1
ZNF251	AC141557.1
ZNF250	AC126182.2
ZNF248	LINC01376
ZNF239	HIST1H3J
ZNF235	AC090695.1
ZNF234	AC073487.1
ZNF233	UBE2L5P
ZNF232	AC005041.1
ZNF230	AC067968.1
ZNF227	RFPL3S
ZNF226	PCDHGA12
ZNF225	AL583722.4
ZNF224	AC008013.1
ZNF223	TAS2R19
ZNF222	NOS2P3
ZNF22	AL627389.1
ZNF219	TCTE1
ZNF213	AL353719.1
ZNF212	AC004461.2
ZNF211	GKN2
ZNF207	CYCSP10
ZNF205	RPSAP13
ZNF202	ZFP57
ZNF200	ABCA9
ZNF20	AC007272.1
ZNF2	AC064807.2
ZNF197; ZNF660-ZNF197	ERVMER34-1
ZNF195	ALG1L2
ZNF19	AL583810.1
ZNF189	AL138995.1
ZNF185	DCDC1
ZNF184	LMO2
ZNF182	AC011498.5
ZNF180	TXNP6
ZNF174	AL121894.2
ZNF17	SNORA18
ZNF169	AC026954.2
ZNF165	AP001266.2

ZNF16	AC005618.1
ZNF146	GYG2
ZNF143	AP001025.1
ZNF142	APOBEC3A
ZNF14	LINC00221
ZNF138	AC118658.2
ZNF134	ARL11
ZNF133	KRTAP5-2
ZNF131	ANO5
ZNF124	AL355922.3
ZNF121	AC073326.1
ZNF12	C3orf20
ZNF114	AC046134.1
ZNF112	AC063944.1
ZNF107	AL162456.1
ZNF101	AL590302.2
ZNF100	PRG2
ZNF10	SCG3
ZMYND8	AC127496.5
ZMYND19	AC132938.5
ZMYND10	WDR72
ZMYM6	PRR35
ZMYM5	P2RX6P
ZMYM3	MIRLET7I
ZMYM2	PSAPL1
ZMIZ2	AC087385.1
ZMIZ1	AL158835.3
ZMAT5	ITGA11
ZMAT4	AC010503.2
ZMAT2	AL158071.4
ZKSCAN8	AL121839.2
ZKSCAN5	AC107067.1
ZKSCAN4	KLHDC1
ZKSCAN3	LINC00441
ZIC5	AC244033.1
ZIC2	CORO2B
ZHX2	OR1F12
ZHX1-C8orf76	TMX2-CTNND1
ZGRF1	SCTR
ZGLP1	ST8SIA6-AS1
ZG16B	AL031731.1
ZFYVE27	AC241644.3
ZFYVE26	TXNRD3NB
ZFYVE19	AC007663.1

ZFR	AC099336.1
ZFPL1	PRAP1
ZFP90	MGARP
ZFP82	FCN2
ZFP69B	AC138356.1
ZFP69	NPC1L1
ZFP64	TTLL13P
ZFP62	MYO16-AS1
ZFP41	AL365226.1
ZFP36L2	AC244034.3
ZFP14	PSPC1P1
ZFHX2	AC018616.1
ZFC3H1	AC105339.2
ZFAT	CELF6
ZFAND3	AGAP11
ZFAND2B	AL358075.4
ZFAND2A	AC040977.2
ZFAND1	C1orf127
ZER1	LINC01776
ZDHHC9	SOCS5P4
ZDHHC7	AC091100.1
ZDHHC6	UBASH3A
ZDHHC5	LRRC32
ZDHHC4	LINC01979
ZDHHC3	FBXO15
ZDHHC24	AC008734.1
ZDHHC18	AL109955.1
ZDHHC17	AC092159.2
ZDHHC16	AL355987.3
ZDHHC14	DAB1
ZDHHC13	F2RL2
ZDHHC12	AL683813.1
ZDHHC1	AC233702.10
ZCRB1	AL139300.1
ZCCHC9	ADAM32
ZCCHC8	AC114755.5
ZCCHC7	AS3MT
ZCCHC4	ATP6V1G2-DDX39B
ZCCHC3	AL158151.1
ZCCHC17	TNXA
ZCCHC12	AC027682.3
ZCCHC10	FP565260.2
ZC3HC1	AC092849.1
ZC3HAV1L	DNAAF4-CCPG1

ZC3H8
ZC3H4
ZC3H3
ZC3H18
ZC3H15
ZC3H12B
ZC3H11A
ZC3H10
ZC2HC1A
ZBTB9
ZBTB8OS
ZBTB8B
ZBTB7A
ZBTB5
ZBTB49
ZBTB48
ZBTB46
ZBTB45
ZBTB41
ZBTB40
ZBTB34
ZBTB32
ZBTB3
ZBTB26
ZBTB24
ZBTB22
ZBTB2
ZBTB17
ZBTB12
ZBTB11
ZBED8
ZBED6CL
ZBED5
ZBED4
ZBED2
ZBBX
ZAR1L
ZACN
YY1AP1
YY1
YWHAZ
YWHAQ
YWHAH
YWHAG

YWHAE
YWHAB
YTHDF2
YTHDF1
YTHDC1
YRDC
YPEL5
YPEL4
YPEL3
YOD1
YKT6
YJU2
YJEFN3
YIPF7
YIPF4
YIPF3
YIPF2
YIPF1
YIF1B
YIF1A
YEATS4
YEATS2
YDJC
YBX3
YBX1
YBEY
YARS2
YARS1
YAE1
XYLT2
XXYLT1
XRN2
XRCC6
XRCC5
XRCC4
XRCC3
XRCC2
XRCC1
XPR1
XPOT
XPO7
XPO6
XPO5
XPO4

XPO1
XPNPEP1
XPA
XIRP1
XCL1
XAB2
WWP2
WWC3
WWC1
WTIP
WTAP
WSCD1
WSB2
WSB1
WRNIP1
WRAP73
WRAP53
WNT6
WNT3A
WNT2B
WNT10B
WNT10A
WNK4
WNK2
WIZ
WIPI2
WIPF2
WIF1
WHRN
WHAMM
WFS1
WFDC3
WFDC13
WFDC10A
WDYHV1
WDSUB1
WDR97
WDR93
WDR92
WDR91
WDR90
WDR89
WDR88
WDR87

WDR83OS
WDR83
WDR82
WDR77
WDR76
WDR75
WDR74
WDR73
WDR70
WDR62
WDR61
WDR60
WDR6
WDR5B
WDR55
WDR54
WDR53
WDR5
WDR48
WDR46
WDR45B
WDR45
WDR43
WDR41
WDR4
WDR38
WDR37
WDR35
WDR34
WDR33
WDR3
WDR27
WDR26
WDR25
WDR18
WDR13
WDR12
WDR1
WDHD1
WDFY2
WDCP
WBP2NL
WBP2
WBP11

WBP1
WASHC5
WASHC2C
WASHC2A
WASHC1
WASF2
WASF1
WARS2
WARS1
WAPL
WAC
VWA5B2
VWA1
VTI1A
VSX2
VSX1
VSTM5
VSIG8
VSIG10L
VSIG10
VSIG1
VRTN
VRK1
VPS9D1
VPS8
VPS72
VPS54
VPS53
VPS51
VPS50
VPS4A
VPS45
VPS41
VPS39
VPS37C
VPS37B
VPS35
VPS33B
VPS33A
VPS29
VPS28
VPS26A
VPS25
VPS18

VPS16
VPS13B
VPS11
VOPP1
VN1R5
VN1R1
VMP1
VMAC
VMA21
VKORC1L1
VIRMA
VIPAS39
VIM
VILL
VIL1
VHL
VGF
VEZT
VEZF1
VENTX
VEGFB
VEGFA
VDR
VDAC3
VDAC2
VDAC1
VCPKMT
VCP
VCL
VBP1
VAX2
VAV3
VAT1
VASP
VASH1
VARS2
VARS1
VAPB
VAPA
VANGL2
VANGL1
VAMP8
VAMP5
VAMP4

VAC14
UXT
UXS1
UVSSA
UTS2
UTRN
UTP6
UTP4
UTP3
UTP25
UTP23
UTP20
UTP18
UTP15
UTP14A
UTP11
UTF1
USP6
USP54
USP5
USP49
USP48
USP46
USP42
USP40
USP39
USP36
USP35
USP3
USP27X
USP24
USP22
USP21
USP20
USP19
USP14
USP11
USP1
USH1G
USF2
USF1
USE1
USB1
UROS

UROD
URM1
URI1
URGCP
URB2
URB1
URAD
UQCRHL
UQCRH
UQCRFS1
UQCRC1
UQCRB
UQCR11
UQCR10
UQCC3
UQCC2
UQCC1
UPP1
UPK3A
UPK2
UPK1A
UPF3B
UPF3A
UPF2
UPF1
UNK
UNG
UNC93B1
UNC5CL
UNC5A
UNC50
UNC45A
UNC13D
UNC13A
UNC119B
UNC119
UMPS
UMODL1
ULK3
ULK1
ULBP1
UIMC1
UHRF2
UHRF1BP1

UHRF1
UGT8
UGGT2
UGGT1
UGCG
UFSP1
UFD1
UFC1
UCP3
UCP2
UCP1
UCN2
UCN
UCKL1
UCK2
UCK1
UCHL5
UCHL3
UCHL1
UBXN7
UBXN6
UBXN2A
UBXN11
UBXN1
UBTF
UBTD2
UBTD1
UBR5
UBQLN4
UBP1
UBOX5
UBN2
UBN1
UBLCP1
UBL7
UBL5
UBL4A
UBIAD1
UBFD1
UBE3D
UBE3B
UBE2Z
UBE2W
UBE2V2

UBE2T
UBE2S
UBE2R2
UBE2Q2L
UBE2Q2
UBE2Q1
UBE2O
UBE2N
UBE2M
UBE2L5
UBE2L3
UBE2K
UBE2J2
UBE2J1
UBE2I
UBE2H
UBE2G2
UBE2F
UBE2E3
UBE2E1
UBE2D2
UBE2D1
UBE2C
UBE2B
UBE2A
UBD
UBASH3B
UBAP2L
UBAP2
UBAP1
UBALD2
UBAC2
UBAC1
UBA52
UBA5
UBA3
UBA2
UBA1
UAP1L1
U2SURP
U2AF2
U2AF1L4
TYW5
TYW3

TYW1
TYSND1
TYROBP
TYRO3
TYMSOS
TYMS
TYK2
TXNRD2
TXNRD1
TXNL4B
TXNL4A
TXNL1
TXNDC9
TXNDC2
TXNDC17
TXNDC12
TXN2
TXN
TXLNG
TXLNA
TWNK
TWISTNB
TWIST1
TWF2
TVP23C
TVP23A
TUT1
TUSC2
TULP4
TULP3
TULP1
TUFT1
TUFM
TUBGCP6
TUBGCP2
TUBG2
TUBG1
TUBD1
TUBB8
TUBB6
TUBB4B
TUBB3
TUBB2B
TUBB2A

TUBB
TUBA1C
TUBA1B
TUBA1A
TTYH3
TTYH2
TTYH1
TTLL9
TTLL6
TTLL5
TTLL4
TTLL3
TTLL12
TTLL11
TTLL1
TTL
TTK
TTI1
TTF2
TTF1
TTC9C
TTC9B
TTC5
TTC4
TTC39A
TTC34
TTC32
TTC3
TTC29
TTC27
TTC26
TTC23L
TTC21B
TTC21A
TTC13
TTC1
TSTD3
TSTA3
TSSK6
TSSC4
TSR3
TSR2
TSR1
TSPYL2

TSPOAP1
TSPO2
TSPO
TSPAN4
TSPAN3
TSPAN19
TSPAN17
TSPAN15
TSPAN14
TSPAN10
TSNAX
TSNARE1
TSN
TSGA10IP
TSGA10
TSG101
TSFM
TSEN54
TSEN34
TSEN2
TSEN15
TSC22D4
TSC1
TSACC
TRUB2
TRRAP
TRPV2
TRPT1
TRPM5
TRPM4
TRPM2
TRPC7
TRPC4AP
TRPC1
TROAP
TRNT1
TRNP1
TRNAU1AP
TRMU
TRMT61A
TRMT6
TRMT5
TRMT44
TRMT2B

TRMT2A
TRMT13
TRMT12
TRMT112
TRMT10C
TRMT10B
TRMT1
TRMO
TRIT1
TRIR
TRIP6
TRIP4
TRIP13
TRIP12
TRIP10
TRIOBP
TRIO
TRIM8
TRIM72
TRIM71
TRIM7
TRIM67
TRIM65
TRIM6
TRIM59
TRIM56
TRIM54
TRIM52
TRIM50
TRIM47
TRIM46
TRIM45
TRIM41
TRIM39-RPP21; RPP21
TRIM39
TRIM37
TRIM33
TRIM32
TRIM31
TRIM3
TRIM28
TRIM27
TRIM25
TRIM24

TRIM17
TRIM16L
TRIM16
TRIM13
TRIM11
TRIB3
TRIAP1
TREX2
TREM2
TREM1
TRDMT1
TRAPPC9
TRAPPC6A
TRAPPC5
TRAPPC4
TRAPPC3
TRAPPC2L
TRAPPC2B
TRAPPC2
TRAPPC12
TRAPPC1
TRAM1L1
TRAM1
TRAK2
TRAK1
TRAIP
TRAFD1
TRAF7
TRAF5
TRAF4
TRAF3
TRAF2
TRAF1
TRADD
TRABD
TRA2B
TRA2A
TPX2
TPT1
TPRN
TPRKB
TPRA1
TPR
TPPP3

TPM4
TPM3
TPM2
TPM1
TPI1
TPH1
TPGS2
TPGS1
TPD52L2
TPD52
TPCN1
TPBGL
TP53TG5
TP53RK
TP53I3
TP53I13
TP53I11
TP53BP2
TP53
TOX4
TOR4A
TOR3A
TOR2A
TOPBP1
TOP3A
TOP2B
TOP2A
TOP1MT
TONSL
TOMM70
TOMM7
TOMM5
TOMM40L
TOMM40
TOMM34
TOMM22
TOMM20L
TOMM20
TOM1L2
TOM1
TOGARAM2
TOE1
TNRC18
TNPO3

TNPO2
TNPO1
TNP1
TNNT2
TNNT1
TNNI3
TNNI2
TNNC1
TNK2
TNIP2
TNIP1
TNFSF9
TNFSF4
TNFSF15
TNFRSF9
TNFRSF8
TNFRSF4
TNFRSF25
TNFRSF21
TNFRSF18
TNFRSF14
TNFRSF13C
TNFRSF12A
TNFRSF11A
TNFRSF10C
TNFAIP8L3
TNFAIP8L2-SCNM1; SCNM1
TNFAIP8
TNFAIP6
TMX3
TMX2
TMUB2
TMUB1
TMSB4X
TMSB15B
TMSB15B
TMSB15A
TMSB10
TMPRSS7
TMPRSS5
TMPO
TMIGD1
TMEM9B
TMEM99

TMEM98
TMEM94
TMEM91
TMEM9
TMEM8B
TMEM89
TMEM88B
TMEM87B
TMEM87A
TMEM81
TMEM80
TMEM79
TMEM74B
TMEM70
TMEM69
TMEM68
TMEM67
TMEM65
TMEM63C
TMEM63B
TMEM63A
TMEM62
TMEM61
TMEM60
TMEM54
TMEM52B
TMEM51
TMEM50B
TMEM50A
TMEM44
TMEM43
TMEM42
TMEM41B
TMEM41A
TMEM39B
TMEM39A
TMEM38B
TMEM31
TMEM272
TMEM268
TMEM267
TMEM266
TMEM263
TMEM262

TMEM260
TMEM259
TMEM258
TMEM256
TMEM253
TMEM251
TMEM250
TMEM249
TMEM248
TMEM243
TMEM241
TMEM240
TMEM237
TMEM236
TMEM234
TMEM230
TMEM225B
TMEM223
TMEM222
TMEM219
TMEM218
TMEM216
TMEM214
TMEM213
TMEM209
TMEM208
TMEM203
TMEM201
TMEM199
TMEM198
TMEM191C
TMEM191B
TMEM190
TMEM189
TMEM186
TMEM185B
TMEM185A
TMEM184B
TMEM183A
TMEM182
TMEM181
TMEM18
TMEM179B
TMEM178A

TMEM177
TMEM17
TMEM168
TMEM167B
TMEM167A
TMEM165
TMEM164
TMEM161A
TMEM160
TMEM158
TMEM155
TMEM151B
TMEM151A
TMEM150A
TMEM14C
TMEM14B
TMEM147
TMEM145
TMEM141
TMEM138
TMEM134
TMEM132A
TMEM128
TMEM127
TMEM126B
TMEM126A
TMEM121B
TMEM120B
TMEM120A
TMEM117
TMEM116
TMEM115
TMEM11
TMEM109
TMEM108
TMEM107
TMEM106C
TMEM104
TMEM102
TMEM101
TMEFF1
TMED9
TMED4
TMED3

TMED2
TMED1
TMCO3
TMCO1
TMCC2
TMC7
TMC6
TMC5
TMC2
TMBIM4
TMBIM1
TMA7
TMA16
TM9SF4
TM9SF3
TM9SF1
TM4SF19
TM4SF1
TM2D1
TLX2
TLR9
TLNRD1
TLL2
TLK2
TLK1
TLE5
TLE3
TLE1
TLDC2
TLCD5
TLCD3B
TLCD3A
TLCD1
TKTL2
TKT
TK1
TJAP1
TIPRL
TIPIN
TINF2
TINAG
TIMMDC1
TIMM9
TIMM8B

TIMM8A
TIMM50
TIMM44
TIMM29
TIMM23
TIMM22
TIMM21
TIMM17B
TIMM17A
TIMM13
TIMM10B
TIMM10
TIMELESS
TIGD7
TIGD6
TIGD5
TIGD3
TIGD1
TIGAR
TICRR
TIAM2
TIAL1
TIA1
THYN1
THY1
THUMPD3
THUMPD2
THOP1
THOC7
THOC6
THOC5
THOC3
THOC2
THOC1
THG1L
THEM6
THEM5
THEM4
THBS3
THAP8
THAP7
THAP4
THAP3
THAP2

THAP12
THAP11
THADA
TGS1
TGM4
TGM3
TGM1
TGIF2
TGIF1
TGFBRAP1
TGFBI
TGFB2
TGFB1
TFRC
TFPT
TFG
TFF3
TFEB
TFE3
TFDP2
TFDP1
TFCP2L1
TFCP2
TFB2M
TFAP4
TFAP2E
TFAP2B
TFAP2A
TFAM
TEX48
TEX46
TEX45
TEX38
TEX29
TEX264
TEX261
TEX22
TEX19
TEX10
TET3
TET1
TESMIN
TESK1
TESC

TES
TERT
TERF2IP
TERF2
TERF1
TERB1
TEPSIN
TENT5B
TENT4A
TELO2
TEKT4
TEKT3
TEKT2
TEFM
TEDC2
TEDC1
TECR
TECPR1
TEAD4
TEAD3
TEAD2
TDRKH
TDRD5
TDRD3
TDRD12
TDP2
TDP1
TDG
TCTN3
TCTN2
TCTN1
TCTEX1D2
TCTE3
TCP11L1
TCP1
TCOF1
TCIRG1
TCHP
TCFL5
TCF7L1
TCF7
TCF3
TCF25
TCF24

TCF20
TCF19
TCF15
TCERG1
TCEANC2
TCEAL9
TCEAL5
TCEAL4
TCEAL3
TCEA1
TBX6
TBX5
TBX4
TBX2
TBX19
TBRG4
TBRG1
TBPL1
TBP
TBL3
TBL2
TBL1XR1
TBKBP1
TBCD
TBCC
TBCB
TBCA
TBC1D7-LOC100130357;
TBC1D7
TBC1D3L; TBC1D3
TBC1D3B; TBC1D3C
TBC1D31
TBC1D30
TBC1D25
TBC1D22B
TBC1D22A
TBC1D20
TBC1D17
TBC1D16
TBC1D13
TBC1D12
TBC1D10B
TBC1D1
TAZ

TAX1BP3
TAX1BP1
TATDN3
TATDN2
TATDN1
TASP1
TASOR2
TASOR
TAS2R5
TAS2R43; TAS2R45
TAS2R4
TAS2R31
TAS2R20
TAS2R19
TAS2R14; PRH1-TAS2R14
TAS2R10
TAS2R1
TARS3
TARS1
TARDBP
TARBP2
TARBP1
TAPBP
TAP2
TAP1
TAOK2
TAOK1
TANGO2
TAMM41
TALDO1
TAL2
TAGLN3
TAGLN2
TAFA3
TAFA2
TAF9
TAF8
TAF7
TAF6
TAF5L
TAF5
TAF4
TAF3
TAF1D

TAF1C
TAF1B
TAF1A
TAF15
TAF12
TAF11
TAF10
TADA3
TADA2A
TACR2
TACC3
TACC2
TAC3
TAB1
SZRD1
SYTL1
SYT8
SYT5
SYT16
SYS1
SYPL1
SYP
SYNRG
SYNPR
SYNJ2BP-COX16
SYNJ2
SYNGR4
SYNGR3
SYNGR2
SYNGR1
SYNGAP1
SYNE4
SYNCRIP
SYN3
SYMPK
SYF2
SYDE1
SYCP2L
SYCP2
SYCE1L
SWT1
SWSAP1
SWI5
SVOPL

SVIP
SVBP
SV2A
SUZ12
SUV39H2
SUV39H1
SUSD1
SURF6
SURF4
SURF2
SUPV3L1
SUPT7L
SUPT6H
SUPT5H
SUPT4H1
SUPT3H
SUPT20H
SUPT16H
SUN1
SUMO3
SUMO2
SUMO1
SUMF2
SULT1C2
SULT1A3
SUGT1
SUGP2
SUGP1
SUFU
SUDS3
SUCO
SUB1
STYXL1
STXBP6
STXBP5L
STXBP5
STXBP4
STXBP2
STX8
STX7
STX6
STX5
STX4
STX3

STX2
STX1A
STX18
STX16
STX10
STT3B
STT3A
STRN4
STRIP1
STRC
STRBP
STRAP
STRADA
STRA8
STRA6
STPG1
STOX1
STOML3
STOML2
STOML1
STN1
STMP1
STMN3
STMN1
STKLD1
STK4
STK39
STK38
STK36
STK35
STK33
STK32C
STK32B
STK3
STK25
STK19
STK17A
STK11IP
STK11
STK10
STIP1
STIMATE-MUSTN1
STIM2
STIL

STEAP1B
STC2
STC1
STAU2
STAU1
STAT5A
STARD9
STARD7
STARD6
STARD3NL
STARD3
STAMBPL1
STAMBP
STAM
STAC3
STAC2
ST8SIA5
ST8SIA1
ST7L
ST6GALNAC5
ST6GALNAC4
ST3GAL4
ST3GAL2
ST20
ST14
SSUH2
SSU72
SSTR5
SSTR3
SSRP1
SSR4
SSR3
SSR2
SSR1
SSNA1
SSH3
SSH2
SSC4D
SSBP4
SSBP1
SSB
SS18L2
SS18
SRXN1

SRSF9
SRSF7
SRSF6
SRSF4
SRSF3
SRSF2
SRSF12
SRSF11
SRSF10
SRSF1
SRRT
SRRM5
SRRM4
SRRM3
SRRD
SRPX2
SRPRB
SRPK3
SRPK2
SRPK1
SRP9
SRP72
SRP68
SRP19
SRP14
SRM
SRI
SRGAP2
SRF
SREK1IP1
SREK1
SREBF2
SRD5A3
SRCAP
SRC
SRA1
SQOR
SQLE
SPTY2D1OS
SPTLC2
SPTBN5
SPTAN1
SPSB2
SPRYD3

SPRTN
SPRN
SPRED3
SPRED1
SPR
SPPL3
SPPL2B
SPP1
SPOUT1
SPOP
SPON2
SPOCK1
SPOCD1
SPIRE2
SPINT1
SPINK4
SPINK13
SPINK1
SPINDOC
SPIN3
SPIN2B
SPIDR
SPICE1
SPIB
SPHK1
SPG7
SPG21
SPEG
SPEF2
SPEF1
SPECC1L
SPECC1
SPDYE6
SPDYE5
SPDYE3
SPDYA
SPDL1
SPDEF
SPCS2
SPCS1
SPC25
SPC24
SPATS2L
SPATS2

SPATC1L
SPATC1
SPATA7
SPATA5L1
SPATA45
SPATA33
SPATA2L
SPATA25
SPATA24
SPATA20
SPATA2
SPATA17
SPATA12
SPAST
SPARC
SPANXC
SPANXB1
SPAG8
SPAG7
SPAG6
SPAG5
SPAG4
SPAG17
SPAG1
SPACA6
SPACA4
SPA17
SP9
SP8
SP6
SP5
SP140L
SP1
SOX9
SOX4
SOX21
SOX2
SOX18
SOX13
SOX12
SOX11
SOWAHD
SORT1
SORCS3

SORCS1
SOGA1
SOCS7
SOCS5
SOCS1
SOBP
SOAT1
SNX8
SNX5
SNX33
SNX32
SNX30
SNX3
SNX27
SNX24
SNX22
SNX21
SNX17
SNX16
SNX15
SNX14
SNX12
SNX11
SNW1
SNURF
SNUPN
SNU13
SNTA1
SNRPN
SNRPG
SNRPF
SNRPE
SNRPD3
SNRPD2
SNRPD1
SNRPC
SNRPB2
SNRPB
SNRPA1
SNRPA
SNRNP70
SNRNP48
SNRNP40
SNRNP35

SNRNP27
SNRNP25
SNRNP200
SNPH
SNORC
SNN
SNHG32
SNF8
SND1
SNCG
SNCB
SNAPIN
SNAPC5
SNAPC4
SNAPC3
SNAPC2
SNAPC1
SNAP91
SNAP47
SNAP29
SNAP25
SMYD5
SMYD4
SMYD3
SMYD2
SMURF2
SMURF1
SMUG1
SMU1
SMTNL2
SMTN
SMS
SMPD4
SMPD2
SMOX
SMNDC1
SMN1; SMN2
SMN1
SMKR1
SMIM8
SMIM7
SMIM5
SMIM4
SMIM32

SMIM30
SMIM3
SMIM29
SMIM27
SMIM26
SMIM23
SMIM22
SMIM20
SMIM15
SMIM13
SMIM10L1
SMG9
SMG7
SMG5
SMDT1
SMCR8
SMCO2
SMCHD1
SMC3
SMC2
SMC1B
SMARCE1
SMARCD3
SMARCD2
SMARCD1
SMARCC1
SMARCB1
SMARCAL1
SMARCA4
SMAP1
SMAGP
SMAD5
SMAD3
SMAD2
SLX4IP
SLX4
SLX1B
SLX1A
SLU7
SLTM
SLN
SLIT1
SLIRP
SLF2

SLF1
SLCO5A1
SLCO4A1
SLCO1C1
SLC9C1
SLC9B1
SLC9A5
SLC9A3R1
SLC9A2
SLC9A1
SLC8A2
SLC7A7
SLC7A6OS
SLC7A6
SLC7A5
SLC7A11
SLC7A10
SLC7A1
SLC6A9
SLC6A8
SLC6A7
SLC6A4
SLC6A3
SLC6A17
SLC6A15
SLC6A14
SLC66A1L
SLC66A1
SLC5A5
SLC5A10
SLC52A2
SLC51B
SLC50A1
SLC4A9
SLC4A8
SLC4A7
SLC4A5
SLC4A3
SLC4A2
SLC4A1AP
SLC4A11
SLC48A1
SLC45A4
SLC44A4

SLC44A3
SLC43A2
SLC41A3
SLC41A1
SLC3A2
SLC39A7
SLC39A6
SLC39A4
SLC39A3
SLC39A13
SLC39A10
SLC39A1
SLC38A9
SLC38A8
SLC38A7
SLC38A6
SLC38A5
SLC38A1
SLC37A3
SLC37A1
SLC36A3
SLC36A1
SLC35G5
SLC35F6
SLC35F3
SLC35F2
SLC35E4
SLC35E3
SLC35E1
SLC35D2
SLC35C2
SLC35B3
SLC35B2
SLC35B1
SLC35A4
SLC35A2
SLC35A1
SLC34A3
SLC30A8
SLC30A6
SLC30A5
SLC30A3
SLC2A8
SLC2A6

SLC2A4RG
SLC2A11
SLC2A1
SLC29A4
SLC29A3
SLC29A2
SLC27A4
SLC26A6
SLC26A2
SLC26A11
SLC25A6
SLC25A53
SLC25A51
SLC25A5
SLC25A45
SLC25A40
SLC25A39
SLC25A36
SLC25A35
SLC25A32
SLC25A3
SLC25A29
SLC25A28
SLC25A26
SLC25A19
SLC25A17
SLC25A14
SLC25A12
SLC25A11
SLC25A1
SLC24A5
SLC22A8
SLC22A5
SLC22A4
SLC22A23
SLC22A16
SLC22A15
SLC20A1
SLC1A5
SLC1A4
SLC19A1
SLC18B1
SLC17A9
SLC17A5

SLC16A6
SLC16A3
SLC15A4
SLC15A2
SLC13A4
SLC12A9
SLC12A8
SLC12A7
SLC12A5
SLC11A2
SLC11A1
SLC10A4
SLC10A3
SLBP
SLAMF8
SKP2
SKP1
SKIV2L
SKA3
SKA2
SKA1
SIX5
SIX4
SIX3
SIX2
SIX1
SIVA1
SIRT7
SIRT6
SIRT4
SIRT2
SIRPG
SIPA1L3
SIPA1L2
SIPA1
SINHCAF
SIN3B
SIN3A
SIM2
SIL1
SIAH3
SIAH1
SHROOM1
SHQ1

SHOX2
SHOC2
SHLD3
SHLD1
SHKBP1
SHISAL2B
SHISAL2A
SHISA8
SHISA5
SHISA2
SHCBP1
SHC3
SHC2
SHC1
SHARPIN
SH3TC1
SH3PXD2B
SH3PXD2A
SH3KBP1
SH3GLB2
SH3GLB1
SH3GL1
SH3D21
SH3BP5L
SH3BP1
SH3BGRL3
SH2D7
SH2D5
SH2D3A
SH2D2A
SH2B2
SH2B1
SGTB
SGTA
SGSM3
SGSM2
SGSM1
SGSH
SGPP2
SGO2
SGO1
SGF29
SGCE
SFXN4

SFXN3
SFT2D2
SFT2D1
SFSWAP
SFRP2
SFR1
SFPQ
SFN
SFI1
SF3B6
SF3B5
SF3B4
SF3B3
SF3B2
SF3B1
SF3A3
SF3A2
SF3A1
SF1
SEZ6L2
SEZ6
SETMAR
SETDB1
SETD6
SETD5
SETD4
SETD1B
SETD1A
SET
SESTD1
SERTAD3
SERPINI2
SERPINI1
SERPINH1
SERPINE2
SERPINB6
SERPINB11
SERPINB1
SERP1
SERHL2
SERGEF
SERF2
SERF1A; SERF1B
SERBP1

SERAC1
SEPTIN9
SEPTIN8
SEPTIN7
SEPTIN5
SEPTIN3
SEPTIN2
SEPTIN1
SEPHS1
SENP6
SENP5
SENP3
SENP1
SEMA7A
SEMA6C
SEMA6B
SEMA5B
SEMA4F
SEMA4D
SEMA4C
SEMA4A
SEMA3F
SEMA3B
SEM1
SELENOW
SELENOS
SELENON
SELENOM
SELENOK
SELENOH
SELENOF
SEL1L3
SEH1L
SECTM1
SEC61G
SEC61B
SEC61A2
SEC61A1
SEC23IP
SEC23B
SEC22C
SEC22A
SEC13
SEC11C

SEC11A
SDHAF4
SDHAF2
SDHAF1
SDF2L1
SDF2
SDCCAG8
SCYL1
SCX
SCUBE3
SCT
SCRT1
SCRIB
SCO2
SCN5A
SCN1B
SCML2
SCMH1
SCLY
SCLT1
SCIN
SCGB2A1
SCG3
SCG2
SCFD2
SCFD1
SCARA3
SCAP
SCAND1
SCAMP5
SCAMP4
SCAMP3
SCAMP2
SCAF1
SBSPON
SBSN
SBNO2
SBNO1
SBK1
SBF1
SAYSD1
SAXO2
SATL1
SASS6

SART3
SART1
SARS2
SARS1
SARNP
SAR1A
SAPCD2
SAPCD1
SAP30BP
SAP30
SAP25
SAP18
SAP130
SAMM50
SAMD4B
SAMD14
SAMD13
SAMD10
SAMD1
SALL4
SALL3
SALL2
SAG
SAFB2
SAFB
SAE1
SACS
SAC3D1
SAAL1
S1PR2
S100PBP
S100P
S100B
S100A9
S100A6
S100A5
S100A4
S100A3
S100A2
S100A16
S100A14
S100A13
S100A11
S100A10

S100A1
RYR3
RYK
RXYLT1
RXRB
RXFP4
RWDD3
RWDD2A
RWDD1
RUVBL2
RUVBL1
RUSC2
RUSC1
RUNX1
RUNDC3A
RUNDC1
RUFY4
RUFY2
RUFY1
RUBCN
RTRAF
RTN4IP1
RTN3
RTN2
RTL8C
RTL8A
RTL10
RTL1
RTKN2
RTKN
RTF2
RTF1
RTCB
RTCA
RSU1
RSRP1
RSRC2
RSRC1
RSPO4
RSPH9
RSPH6A
RSPH4A
RSPH14
RSPH1

RSL24D1
RSL1D1
RSKR
RSBN1L
RSAD1
RS1
RRS1
RRP9
RRP8
RRP7A
RRP36
RRP1B
RRP15
RRP12
RRP1
RRNAD1
RRM2
RRM1
RRBP1
RRAS
RRAGD
RRAGC
RRAGB
RRAGA
RPUSD4
RPUSD3
RPUSD2
RPUSD1
RPTOR
RPSA
RPS9
RPS8
RPS7
RPS6KL1
RPS6KC1
RPS6KB2
RPS6KB1
RPS6KA4
RPS6
RPS5
RPS4X
RPS3A
RPS3
RPS29

RPS28
RPS27A
RPS27
RPS26
RPS25
RPS24
RPS23
RPS21
RPS20
RPS2
RPS19BP1
RPS19
RPS18
RPS17
RPS16
RPS15A
RPS15
RPS14
RPS13
RPS12
RPS11
RPS10
RPRM
RPRD2
RPRD1A
RPP40
RPP38-DT
RPP38
RPP30
RPP25L
RPP25
RPN2
RPN1
RPLP2
RPLP1
RPLP0
RPL9
RPL8
RPL7L1
RPL7A
RPL7
RPL6
RPL5
RPL41

RPL4
RPL39L
RPL39
RPL38
RPL37A
RPL37
RPL36AL
RPL36A-HNRNPH2
RPL36A
RPL36
RPL35A
RPL35
RPL34
RPL32
RPL31
RPL30
RPL3
RPL29
RPL28
RPL27A
RPL27
RPL26L1
RPL26
RPL24
RPL23A
RPL23
RPL22L1
RPL22
RPL21
RPL19
RPL18A
RPL18
RPL17
RPL15
RPL14
RPL13A
RPL13
RPL12
RPL11
RPL10A
RPL10
RPIA
RPGRIP1L
RPGR

RPF2
RPF1
RPE
RPAP3
RPAP2
RPAP1
RPAIN
RPA3
RPA2
RPA1
RP9
ROMO1
ROM1
ROBO3
ROBO1
RNPS1
RNPEPL1
RNPEP
RNPC3
RNMT
RNH1
RNFT2
RNF8
RNF7
RNF5
RNF44
RNF41
RNF4
RNF39
RNF34
RNF32
RNF26
RNF25
RNF24
RNF227
RNF225
RNF224
RNF222
RNF220
RNF216
RNF215
RNF214
RNF207
RNF20

RNF2
RNF187
RNF183
RNF181
RNF167
RNF166
RNF157
RNF149
RNF148
RNF145
RNF144A
RNF139
RNF126
RNF122
RNF121
RNF115
RNF114
RNF113A
RNF103-CHMP3
RNF10
RNASET2
RNASEK
RNASEH2C
RNASEH2B
RNASEH2A
RNASEH1
RNASE1
RMND5B
RMI2
RMDN1
RLN2; RLN1
RITA1
RIT1
RIPPLY2
RIPOR1
RIPK2
RIOX2
RIOX1
RIOK2
RIOK1
RINT1
RINL
RING1
RIN1

RIMS3
RIMBP3C
RIMBP3B
RILPL1
RIF1
RIC8B
RIC8A
RIBC2
RIBC1
RHPN1
RHOV
RHOT2
RHOT1
RHOQ
RHOG
RHOF
RHOC
RHOBTB2
RHOA
RHO
RHNO1
RHEBL1
RHEB
RHBDL2
RHBDL1
RHBDF2
RHBDF1
RHBDD3
RHBDD2
RGS9BP
RGS3
RGS20
RGS2
RGS19
RGS17
RGS14
RGS12
RGS10
RGR
RGP1
RGL4
RGL3
RGL2
RFXAP

RFXANK
RFX8
RFX7
RFX6
RFX5
RFX4
RFX3
RFX2
RFX1
RFWD3
RFT1
RFNG
RFLNA; ZNF664-RFLNA
RFC5
RFC4
RFC3
RFC2
RFC1
REXO5
REXO4
REXO2
REXO1
REX1BD
RETREG3
RETREG2
RERE
REPIN1
REP15
RENBP
REM2
RELT
RELL2
RELCH
RELB
RELA
REG4
REG1A
REEP4
REEP2
RECQL5
RECQL4
REC8
RDM1
RDH14

RDH13
RCOR3
RCOR2
RCN3
RCN2
RCN1
RCE1
RCCD1
RCC2
RCC1L
RCC1
RCAN3
RBX1
RBSN
RBPMS
RBPJL
RBPJ
RBP2
RBMX2
RBMX
RBMS1
RBM8A
RBM6
RBM5
RBM4B
RBM48
RBM45
RBM44
RBM42
RBM4
RBM39
RBM38
RBM34
RBM33
RBM3
RBM28
RBM27
RBM26
RBM25
RBM24
RBM22
RBM19
RBM18
RBM17

RBM15B
RBM15
RBM14-RBM4
RBM14
RBM12B
RBM12
RBM10
RBL1
RBIS
RBFOX3
RBFOX2
RBFA
RBCK1
RBBP8
RBBP7
RBBP5
RBBP4
RAVER2
RASSF7
RASSF4
RASSF3
RASSF1
RASL12
RASL10A
RASGEF1A
RASD2
RASAL3
RASAL2
RASAL1
RASA4
RARS2
RARS1
RARG
RARA
RAPSN
RAPGEFL1
RAPGEF6
RAPGEF1
RAP2B
RAP2A
RAP1GAP2
RAP1GAP
RANGRF
RANGAP1

RANBP3
RANBP1
RAN
RAMAC
RALY
RALGDS
RALGAPB
RALB
RALA
RAI1
RAE1
RADIL
RAD9A
RAD54L
RAD54B
RAD52
RAD51D
RAD51C
RAD51AP1
RAD51
RAD23A
RAD21
RAD18
RAD17
RAD1
RACK1
RACGAP1
RAC1
RABL6
RABL2B
RABL2A
RABIF
RABGGTB
RABGGTA
RABGEF1
RABEP2
RABAC1
RAB9B
RAB7B
RAB7A
RAB6B
RAB5IF
RAB5C
RAB5B

RAB4B
RAB4A
RAB42
RAB41
RAB40C
RAB40B
RAB40AL
RAB40A
RAB3IP
RAB3IL1
RAB3GAP2
RAB3GAP1
RAB3D
RAB3B
RAB3A
RAB35
RAB34
RAB33A
RAB32
RAB2A
RAB29
RAB28
RAB24
RAB22A
RAB21
RAB1B
RAB1A
RAB15
RAB13
RAB11FIP5
RAB11FIP4
RAB11B
RAB11A
RAB10
R3HDM4
R3HDM1
R3HCC1L
R3HCC1
QTRT2
QTRT1
QSOX2
QSOX1
QSER1
QRSL1

QRICH2
QRICH1
QRFP
QPCTL
QPCT
QARS1
PYURF
PYROXD1
PYM1
PYGO2
PYGB
PYCR3
PYCR2
PYCR1
PYCARD
PXYLP1
PXN
PXMP4
PWWP3A
PWWP2B
PWWP2A
PWP1
PVR
PUSL1
PUS7L
PUS7
PUS1
PURG
PURB
PUM3
PUF60
PUDP
PTTG1IP
PTTG1
PTRHD1
PTRH2
PTRH1
PTPRN
PTPRG
PTPRA
PTPN7
PTPN6
PTPN23
PTPN2

PTPN18
PTPN14
PTPN12
PTPN1
PTPMT1
PTPDC1
PTPA
PTP4A3
PTP4A2
PTOV1
PTMA
PTK7
PTK2
PTHLH
PTGFRN
PTGES3L-AARSD1
PTGES3L
PTGES3
PTGES2
PTGES
PTGDR2
PTF1A
PTDSS2
PTDSS1
PTCD3
PTCD1
PTBP3
PTBP2
PTBP1
PSTPIP1
PSTK
PSRC1
PSPN
PSPH
PSPC1
PSORS1C2
PSORS1C1
PSMG4
PSMG3
PSMG2
PSMG1
PSMF1
PSME4
PSME3IP1

PSME3
PSME2
PSME1
PSMD9
PSMD8
PSMD7
PSMD6
PSMD4
PSMD3
PSMD2
PSMD14
PSMD13
PSMD11
PSMD10
PSMD1
PSMC6
PSMC5
PSMC4
PSMC3IP
PSMC3
PSMC2
PSMB7
PSMB6
PSMB5
PSMB4
PSMB3
PSMB2
PSMB1
PSMA7
PSMA5
PSMA4
PSMA3
PSMA2
PSMA1
PSKH1
PSIP1
PSG6
PSENEN
PSEN2
PSEN1
PSD
PSCA
PSAPL1
PSAP

PRXL2C
PRXL2B
PRX
PRUNE1
PRTFDC1
PRSS41
PRSS38
PRSS27
PRSS21
PRSS2
PRRX2
PRRX1
PRRT3
PRRT2
PRRG3
PRRC2C
PRRC2A
PRR7
PRR5
PRR4; PRH1
PRR36
PRR35
PRR3
PRR29
PRR22
PRR19
PRR15L
PRR14L
PRR14
PRR13
PRR12
PRR11
PRPSAP2
PRPH2
PRPH
PRPF8
PRPF6
PRPF4B
PRPF40B
PRPF40A
PRPF4
PRPF39
PRPF38B
PRPF38A

PRPF31
PRPF3
PRPF19
PRPF18
PROSER3
PROSER1
PROCA1
PROB1
PRMT7
PRMT5
PRMT3
PRMT2
PRMT1
PRLHR
PRL
PRKRIP1
PRKRA
PRKDC
PRKD2
PRKCSH
PRKCI
PRKCG
PRKCD
PRKCA
PRKAR1B
PRKAG3
PRKAG1
PRKAB1
PRKAA2
PRIM2
PRIM1
PRICKLE4
PRICKLE3
PREP
PRELID3B
PRELID3A
PRELID2
PRELID1
PREB
PRDX5
PRDX4
PRDX2
PRDX1
PRDM9

PRDM4
PRDM15
PRDM13
PRDM12
PRDM11
PRDM10
PRCC
PRC1
PRAME
PRAG1
PRAF2
PRADC1
PRAC2
PQBP1
PPWD1
PPT2
PPT1
PPRC1
PPP6R3
PPP6R2
PPP6R1
PPP6C
PPP5C
PPP4R1
PPP4C
PPP3CB
PPP2R5E
PPP2R5D
PPP2R5B
PPP2R5A
PPP2R3C
PPP2R3B
PPP2R3A
PPP2R2D
PPP2R2C
PPP2R1A
PPP2CA
PPP1R9B
PPP1R9A
PPP1R8
PPP1R7
PPP1R42
PPP1R3F
PPP1R37

PPP1R35
PPP1R21
PPP1R2
PPP1R1B
PPP1R18; LOC107987457
PPP1R16A
PPP1R15A
PPP1R14D
PPP1R14C
PPP1R14B
PPP1R13L
PPP1R13B
PPP1R12C
PPP1R12A
PPP1R11
PPP1CC
PPP1CB
PPP1CA
PPOX
PPME1
PPM1N
PPM1M
PPM1G
PPM1F
PPM1D
PPIP5K1
PPIL6
PPIL3
PPIL2
PPIL1
PPIH
PPIG
PPIE
PPIC
PPIB
PPIAL4C
PPIAL4A
PPIA
PPHLN1
PPFIA4
PPFIA3
PPEF1
PPDPF
PPCDC

PPAT
PPARG
PPARD
PPAN
PPA1
POU6F1
POU5F2
POU5F1
POU3F3
POU3F2
POU2F1
POTEI
POTEF
PORCN
POPDC3
POPDC2
POP7
POP5
POP4
POP1
PON2
POMZP3
POMT2
POMT1
POMP
POMGNT2
POMGNT1
POM121L2
POM121C
POLRMT
POLR3K
POLR3H
POLR3G
POLR3F
POLR3E
POLR3D
POLR3C
POLR3A
POLR2M
POLR2L
POLR2K
POLR2J
POLR2I
POLR2H

POLR2G
POLR2F
POLR2E
POLR2D
POLR2C
POLR2B
POLR1E
POLR1D
POLR1C
POLR1B
POLR1A
POLQ
POLM
POLL
POLG2
POLE4
POLE3
POLE2
POLE
POLDIP3
POLDIP2
POLD3
POLD2
POLD1
POLA2
POLA1
POGZ
POGLUT2
POGK
POFUT2
PODXL2
PODNL1
POC5
POC1B
POC1A
PNRC2
PNPT1
PNPLA6
PNPLA1
PNO1
PNN
PNMA3
PNMA1
PNLDC1

PNKP
PNISR
PNCK
PMVK
PMS1
PMPCB
PMPCA
PMM1
PML
PMFBP1
PMF1-BGLAP
PMF1
PMEPA1
PMCH
PMAIP1
PM20D2
PLXND1
PLXNB1
PLXNA3
PLXNA2
PLXNA1
PLXDC1
PLVAP
PLTP
PLSCR3
PLRG1
PLPPR5
PLPPR3
PLPPR2
PLPP2
PLP2
PLOD3
PLOD2
PLOD1
PLK5
PLK4
PLK3
PLK1
PLIN3
PLGRKT
PLEKHS1
PLEKHO2
PLEKHO1
PLEKHN1

PLEKHM2
PLEKHM1
PLEKHJ1
PLEKHH3
PLEKHH1
PLEKHG5
PLEKHG4B
PLEKHG4
PLEKHG3
PLEKHG2
PLEKHF2
PLEKHB2
PLEKHB1
PLEKHA8
PLEC
PLD6
PLD3
PLD2
PLCXD1
PLCG1
PLCE1
PLCD4
PLCD3
PLCB3
PLCB1
PLBD2
PLBD1
PLAUR
PLAU
PLAGL2
PLAG1
PLAC9
PLAC8L1
PLAC1
PLA2G7
PLA2G6
PLA2G4F
PLA2G4E
PLA2G4D
PLA2G15
PKP4
PKP3
PKNOX1
PKN3

PKN1
PKMYT1
PKM
PKIG
PKIB
PKIA
PKDCC
PKD1
PJVK
PJA1
PIWIL4
PITX3
PITX2
PITX1
PITRM1
PITPNB
PITHD1
PISD
PIP5KL1
PIP5K1C
PIP5K1A
PIP4P2
PIP4P1
PIP4K2C
PIP4K2B
PIP4K2A
PINX1
PINLYP
PIN4
PIN1
PIMREG
PIM3
PIM2
PILRB
PIK3R6
PIK3R2
PIK3IP1
PIK3C3
PIK3C2B
PIH1D2
PIH1D1
PIGZ
PIGX
PIGW

PIGU
PIGT
PIGS
PIGM
PIGL
PIGH
PIGG
PIGF
PIGC
PIGBOS1
PIGA
PIFO
PIF1
PIEZO1
PIDD1
PICK1
PIAS4
PIAS3
PIAS2
PIANP
PI4KB
PI4K2A
PI3
PI15
PHYHIPL
PHTF2
PHTF1
PHRF1
PHPT1
PHOSPHO2
PHLDB3
PHLDA2
PHKG2
PHKG1
PHKA2
PHIP
PHF5A
PHF23
PHF21B
PHF21A
PHF20L1
PHF20
PHF19
PHF14

PHF13
PHF12
PHF11
PHF10
PHF1
PHEX
PHETA2
PHETA1
PHC2
PHC1
PHB2
PHB
PHAX
PGS1
PGP
PGM3
PGM2L1
PGLYRP4
PGLS
PGK1
PGGHG
PGF
PGD
PGC
PGBD4
PGBD2
PGBD1
PGAP6
PGAP2
PGAM5
PFN4
PFN1
PFKP
PFKM
PFKL
PFKFB4
PFKFB2
PFDN6
PFDN5
PFDN4
PFDN2
PFDN1
PFAS
PEX5L

PEX26
PEX2
PEX16
PEX11B
PET117
PET100
PES1
PERP
PERM1
PELP1
PELO
PELI1
PEG10
PEF1
PEA15
PDZD7
PDZD11
PDSS1
PDRG1
PDPR
PDLIM7
PDLIM4
PDLIM1
PDK1
PDIA4
PDIA3
PDIA2
PDHB
PDHA2
PDHA1
PDGFRL
PDGFRB
PDGFB
PDGFA
PDE9A
PDE7A
PDE6D
PDE6C
PDE6B
PDE6A
PDE5A
PDE4C
PDE4A
PDE1C

PDCL3
PDCL2
PDCL
PDCD7
PDCD6
PDCD5
PDCD4
PDCD2L
PDCD2
PDCD11
PDCD10
PDCD1
PDAP1
PCYT1A
PCYOX1L
PCSK1N
PCP2
PCNX4
PCNX3
PCNX2
PCNT
PCNP
PCNA
PCMTD2
PCMT1
PCLAF
PCIF1
PCID2
PCGF6
PCGF3
PCGF2
PCGF1
PCF11
PCED1B
PCED1A
PCDHGA6
PCDHGA1
PCDHB9
PCDHB2
PCDHB10
PCDHA9
PCDHA7
PCDHA5
PCDHA4

PCDHA3
PCDHA2
PCDHA1
PCDH8
PCBP4
PCBP2
PBXIP1
PBX4
PBX2
PBK
PBDC1
PAXX
PAXIP1
PAXBP1
PAX9
PAX8
PAX6
PAWR
PATZ1
PASK
PARVG
PARVB
PARS2
PARPBP
PARP2
PARP12
PARP1
PARN
PARL
PARK7
PARG
PARD6G
PARD6B
PARD3
PAQR8
PAQR6
PAQR5
PAQR4
PAPSS1
PAPOLG
PAPOLA
PAPLN
PANX3
PANO1

PANK2
PAN3
PAM16
PALM
PALD1
PALB2
PAK4
PAK2
PAK1IP1
PAK1
PAIP2
PAIP1
PAGR1
PAFAH1B3
PAFAH1B2
PAFAH1B1
PAF1
PAEP
PACSIN3
PACSIN2
PACSIN1
PACS2
PACS1
PACRGL
PACC1
PABPN1
PABPC4
PABPC3
PABPC1L
PABPC1
PAAF1
PA2G4
P4HA2
P4HA1
P3H4
P3H1
P2RY6
P2RY11
P2RX5
P2RX4
OXTR
OXLD1
OXCT2
OXA1L

OVOL3
OVGP1
OTX1
OTULIN
OTUD7B
OTUD6B
OTUD5
OTUD3
OTUB1
OTP
OTOG
OSTC
OST4
OSR2
OSGIN2
OSGEPL1
OSGEP
OSER1
OSBPL7
OSBPL3
OSBPL2
OSBP2
ORMDL2
ORMDL1
ORC6
ORC5
ORC4
ORC3
ORC2
ORC1
ORAI2
ORAI1
OR7C1
OR7A5
OR5V1
OR56A5
OR51E2
OR51E1
OR2H2
OR2H1
OR2B6
OR2B2
OR2AG2
OR1Q1

OR1J1
OR1F1
OR14J1
OR13A1
OR12D2
OR12D1
OR11H7
OR11A1
OR10Q1
OPTN
OPRM1
OPRL1
OPRD1
OPN1SW
OPA3
OLIG2
OLFML2B
OLFML2A
OLFM4
OLA1
OIP5
OGT
OGG1
OGFR
OGFOD3
OGFOD2
OGFOD1
OGA
OFD1
ODR4
ODF2
ODC1
ODAPH
OCRL
OCA2
OBSL1
OBSCN
OBP2A
OBI1
OAZ3
OAZ1
OARD1
NYAP1
NXT1

NXPH4
NXPH3
NXNL2
NXF1
NVL
NUTM2G
NUTF2
NUSAP1
NUP93
NUP88
NUP85
NUP62
NUP54
NUP50
NUP43
NUP42
NUP37
NUP35
NUP214
NUP210
NUP205
NUP188
NUP160
NUP155
NUP133
NUP107
NUMBL
NUFIP2
NUFIP1
NUF2
NUDT5
NUDT4
NUDT3
NUDT22
NUDT21
NUDT2
NUDT19
NUDT18
NUDT17
NUDT16L1
NUDT15
NUDT14
NUDT11
NUDT1

NUDCD3
NUDCD1
NUDC
NUCKS1
NUBP2
NUB1
NUAK2
NUAK1
NTS
NTPCR
NTNG2
NTNG1
NTMT1
NT5M
NT5DC4
NT5DC3
NT5DC2
NT5C3B
NT5C3A
NT5C
NSUN7
NSUN5
NSUN4
NSUN2
NSRP1
NSMF
NSMCE4A
NSMCE2
NSMCE1
NSMAF
NSL1
NSG2
NSFL1C
NSF
NSDHL
NSD2
NSD1
NSA2
NRSN2
NRSN1
NRM
NRIP3
NRIP2
NRGN

NRF1
NREP
NRDC
NRBP1
NRAS
NRARP
NR6A1
NR5A1
NR2F6
NR2C2AP
NR2C1
NR1H3
NR1H2
NR0B1
NQO1
NPTX1
NPTN
NPRL3
NPRL2
NPPB
NPPA
NPM3
NPM1
NPLOC4
NPIPB5
NPIPB13
NPIPB11
NPIPA3
NPIPA1
NPHS2
NPHS1
NPHP3
NPHP1
NPFFR1
NPEPPS
NPEPL1
NPDC1
NPC2
NPC1
NPAS2
NPAS1
NOXRED1
NOXA1
NOX4

NOX3
NOX1
NOVA1
NOTCH1
NOSIP
NOP9
NOP58
NOP56
NOP53
NOP2
NOP16
NOP14
NOP10
NONO
NOMO1
NOM1
NOLC1
NOL9
NOL8
NOL7
NOL6
NOL4L
NOL3
NOL12
NOL11
NOL10
NOD1
NOC4L
NOC3L
NOC2L
NOB1
NOA1
NMU
NMT1
NME9
NME8
NME7
NME6
NME4
NME3
NME2
NME1-NME2
NME1
NMB

NLRP1
NLK
NLGN3
NLGN1
NLE1
NKX6-1
NKX3-2
NKX2-5
NKX2-2
NKTR
NKRF
NKPD1
NKIRAS2
NKAPD1
NKAP
NKAIN4
NKAIN1
NISCH
NIPSNAP3B
NIPSNAP2
NIPBL
NIPAL3
NIPAL2
NIPA1
NIP7
NINJ1
NIN
NIFK
NIF3L1
NICN1
NIBAN2
NHP2
NHLH2
NHLH1
NGRN
NGLY1
NGDN
NFYC
NFYA
NFXL1
NFX1
NFU1
NFS1
NFRKB

NFKBIL1
NFKBIE
NFKBID
NFKBIB
NFKB2
NFE2L3
NFE2
NFATC4
NFATC2IP
NFATC2
NF2
NEUROG3
NEUROD2
NEURL4
NEURL3
NEURL2
NEURL1
NEU1
NETO2
NES
NEPRO
NENF
NEMP2
NEMP1
NELL1
NELFE
NELFCD
NELFB
NELFA
NEK8
NEK6
NEK3
NEK2
NEK11
NEIL3
NEFL
NEFH
NEDD9
NEDD8-MDP1
NEDD8
NEDD4L
NEDD1
NECTIN2
NECTIN1

NECAP2
NECAB3
NDUFV3
NDUFV1
NDUFS8
NDUFS7
NDUFS6
NDUFS5
NDUFS4
NDUFS3
NDUFC2
NDUFC1
NDUFB9
NDUFB8
NDUFB7
NDUFB6
NDUFB5
NDUFB4
NDUFB3
NDUFB2
NDUFB11
NDUFB10
NDUFB1
NDUFAF8
NDUFAF7
NDUFAF6
NDUFAF5
NDUFAF4
NDUFAF3
NDUFAF2
NDUFA9
NDUFA8
NDUFA7
NDUFA6
NDUFA4L2
NDUFA4
NDUFA3
NDUFA2
NDUFA13
NDUFA12
NDUFA11
NDUFA10
NDUFA1
NDST2

NDRG3
NDRG1
NDP
NDOR1
NDE1
NDC80
NDC1
NCSTN
NCS1
NCOR2
NCOA6
NCOA5
NCOA3
NCLN
NCL
NCKIPSD
NCKAP5L
NCK2
NCF4
NCF2
NCDN
NCCRP1
NCBP3
NCBP2AS2
NCBP2
NCBP1
NCAPH2
NCAPH
NCAPG2
NCAPG
NCAPD3
NCAPD2
NBPF20
NBPF19
NBN
NBL1; MICOS10-NBL1
NBEAL2
NBDY
NBAS
NAXE
NAT9
NAT8L
NAT14
NAT10

NASP
NARS1
NARF
NAPSA
NAPG
NAPB
NAP1L4
NAP1L1
NANS
NANP
NANOS3
NANOS1
NANOG
NAIF1
NAGPA
NAGK
NAE1
NADSYN1
NACC2
NACC1
NACA2
NACA
NABP2
NABP1
NAB2
NAALADL1
NAA80
NAA60
NAA50
NAA40
NAA38
NAA35
NAA25
NAA20
NAA16
NAA15
NAA10
N6AMT1
N4BP3
N4BP2L2
N4BP1
MZT2B
MZT2A
MZT1

MZF1
MYT1
MYRF
MYPOP
MYPN
MYOZ3
MYO9B
MYO5C
MYO1H
MYO1G
MYO1C
MYO1A
MYO19
MYO15B
MYNN
MYLK3
MYLK2
MYLIP
MYL6B
MYL6
MYL5
MYL4
MYL12B
MYL12A
MYH7
MYH15
MYH13
MYG1
MYEF2
MYDGF
MYCNOS
MYCN
MYCBPAP
MYCBP
MYC
MYBPHL
MYBPC3
MYBPC2
MYBL2
MYBL1
MYBBP1A
MYB
MYADML2
MYADM

MXRA7
MXD4
MXD3
MXD1
MVP
MVK
MVD
MVB12B
MVB12A
MUTYH
MUS81
MUC22
MUC19
MUC12
MUC1
MTX3
MTX2
MTX1
MTRF1L
MTREX
MTRES1
MTR
MTPN
MTPAP
MTMR3
MTMR2
MTMR14
MTMR11
MTMR1
MTLN
MTIF3
MTIF2
MTHFSD
MTHFR
MTHFD2
MTHFD1L
MTG2
MTG1
MTFR2
MTFP1
MTFMT
MTF2
MTERF4
MTERF3

MTERF2
MTERF1
MTDH
MTCL1
MTCH1
MTBP
MTA3
MTA2
MTA1
MSX1
MSTO1
MSS51
MSRB2
MSMB
MSL3
MSL2
MSL1
MSI2
MSI1
MSH6
MSH5
MSH4
MSH2
MSANTD3
MSANTD2
MSANTD1
MS4A8
MS4A15
MRTO4
MRTFA
MRS2
MRRF
MRPS9
MRPS7
MRPS6
MRPS5
MRPS35
MRPS34
MRPS33
MRPS30
MRPS28
MRPS27
MRPS26
MRPS25

MRPS24
MRPS23
MRPS22
MRPS21
MRPS2
MRPS18C
MRPS18B
MRPS18A
MRPS17
MRPS16
MRPS15
MRPS14
MRPS12
MRPS11
MRPS10
MRPL9
MRPL58
MRPL57
MRPL55
MRPL53
MRPL52
MRPL51
MRPL50
MRPL48
MRPL47
MRPL45
MRPL44
MRPL43
MRPL42
MRPL40
MRPL4
MRPL38
MRPL37
MRPL36
MRPL35
MRPL33
MRPL32
MRPL30
MRPL3
MRPL28
MRPL27
MRPL24
MRPL22
MRPL21

MRPL20
MRPL2
MRPL18
MRPL17
MRPL15
MRPL14
MRPL13
MRPL12
MRPL11
MRPL10
MRPL1
MROH6
MROH1
MRNIP
MRM3
MRM2
MRM1
MRI1
MRGBP
MRFAP1L1
MRFAP1
MRE11
MRAP2
MPZL3
MPZL1
MPV17L2
MPV17
MPRIP
MPP6
MPP4
MPP3
MPP2
MPI
MPHOSPH6
MPHOSPH10
MPDU1
MPC2
MOV10
MOSPD3
MOSPD1
MORN2
MORN1
MORF4L2
MORF4L1

MORC2
MON1A
MOK
MOGS
MOCS3
MOB4
MOB3C
MOB3A
MOB2
MOB1A
MNX1
MNT
MND1
MNAT1
MMS22L
MMS19
MMP9
MMP3
MMP26
MMP24OS
MMP17
MMP14
MMP13
MMP12
MMP11
MMP10
MMP1
MMEL1
MMD
MLXIP
MLST8
MLLT6
MLLT3
MLLT10
MLLT1
MLKL
MLH3
MLH1
MLF2
MLEC
MKS1
MKRN3
MKRN2OS
MKRN2

MKRN1
MKNK2
MKNK1
MKKS
MKI67
MIXL1
MITD1
MISP
MIS18BP1
MIS18A
MIS12
MIPOL1
MIOX
MINK1
MINDY4
MINAR1
MIIP
MIGA2
MIF4GD
MIF
MIER2
MIEN1
MIEF2
MIEF1
MIDN
MID1IP1
MID1
MICOS13
MICOS10
MICB
MICALL2
MICALL1
MICAL1
MICA
MIB2
MIA
MGST3
MGME1
MGAT5B
MGAT5
MGAT4A
MGAT1
MGA
MFSD6

MFSD5
MFSD4B
MFSD4A
MFSD3
MFSD2B
MFSD14C
MFSD14B
MFSD13A
MFSD12
MFSD10
MFNG
MFN1
MFHAS1
MFGE8
MFF
MFAP2
MFAP1
MEX3D
MEX3C
MEX3A
METTL9
METTL8
METTL6
METTL5
METTL4
METTL3
METTL2B
METTL2A
METTL26
METTL25
METTL23
METTL22
METTL21A
METTL18
METTL17
METTL16
METTL11B
METTL1
METRNL
METAP2
METAP1D
METAP1
MEST
MESP2

MESP1
MESD
MEPCE
MEP1A
MEN1
MEMO1
MELTF
MELK
MEIS3
MEIS2
MEIS1
MEIG1
MEGF8
MEGF11
MEF2D
MEF2B; BORCS8-MEF2B
MEF2B
MED9
MED8
MED7
MED6
MED4
MED31
MED30
MED29
MED28
MED27
MED26
MED25
MED24
MED22
MED21
MED20
MED19
MED17
MED16
MED15
MED12
MED11
MED10
MED1
MECR
MECP2
MEAK7

MEA1
ME3
ME2
MDS2
MDP1
MDM4
MDK
MDH2
MDH1B
MDFI
MDC1
MCUR1
MCUB
MCU
MCTS1
MCRS1
MCRIP1
MCOLN3
MCOLN1
MCMDC2
MCMBP
MCM8
MCM7
MCM6
MCM5
MCM4
MCM3AP
MCM3
MCM2
MCM10
MCIDAS
MCF2L
MCCD1
MCAT
MCAM
MC1R
MBOAT7
MBLAC1
MBD6
MBD5
MBD3
MBD2
MBD1
MAZ

MAX
MAVS
MAU2
MATR3
MATN4
MATN3
MATN1
MASTL
MAST2
MARVELD1
MARS2
MARS1
MARK4
MARK3
MARK2
MARCKSL1
MARCKS
MARCHF9
MARCHF7
MARCHF4
MARCHF3
MARCHF10
MAPT
MAPRE1
MAPKBP1
MAPKAPK5
MAPKAPK3
MAPKAPK2
MAPKAP1
MAPK9
MAPK8IP3
MAPK8IP2
MAPK7
MAPK3
MAPK1IP1L
MAPK15
MAPK14
MAPK13
MAPK12
MAPK11
MAPK1
MAP7D2
MAP7D1
MAP6D1

MAP4K5
MAP4K4
MAP4K2
MAP4
MAP3K9
MAP3K8
MAP3K7
MAP3K6
MAP3K4
MAP3K3
MAP3K12
MAP3K11
MAP3K10
MAP3K1
MAP2K7
MAP2K5
MAP2K2
MAP1S
MAP1LC3B2
MAP1LC3B
MAP1A
MAP11
MANSC4
MANF
MANBAL
MANBA
MAN2B1
MAN1B1
MAMSTR
MAML1
MALT1
MALSU1
MAL2
MAK16
MAK
MAGOHB
MAGOH
MAGEF1
MAGED4B
MAGED4
MAGED2
MAGED1
MAGEA10-MAGEA5
MAGEA10

MAFG
MAFA
MAF1
MAEA
MADD
MAD2L2
MAD2L1BP
MAD2L1
MAD1L1
MACROH2A2
MACROH2A1
MACROD2
MACIR
M1AP
LZTS3
LZTS2
LZTS1
LZTR1
LYZ
LYSMD4
LYSMD1
LYRM4
LYRM2
LYPLAL1
LYPLA2
LYPD4
LYPD3
LYPD1
LYN
LYL1
LYG2
LYG1
LYAR
LY6K
LY6H
LY6G6C
LY6G5C
LY6G5B
LVRN
LUZP1
LUC7L3
LUC7L2
LUC7L
LTV1

LTO1
LTBR
LTB
LTA4H
LTA
LST1
LSP1
LSMEM1
LSM8
LSM7
LSM6
LSM5
LSM4
LSM3
LSM2
LSM14B
LSM14A
LSM12
LSM11
LSM10
LSM1
LSG1
LRWD1
LRTM2
LRSAM1
LRRIQ1
LRRFIP2
LRRD1
LRRC8B
LRRC75B
LRRC75A
LRRC74B
LRRC74A
LRRC73
LRRC71
LRRC69
LRRC61
LRRC59
LRRC57
LRRC56
LRRC47
LRRC46
LRRC45
LRRC42

LRRC41
LRRC39
LRRC37B
LRRC37A3
LRRC37A
LRRC36
LRRC27
LRRC26
LRRC23
LRRC14
LRRC10B
LRRC1
LRR1
LRPPRC
LRPAP1
LRP8
LRP5L
LRP12
LRP11
LRP10
LRMDA
LRIG2
LRGUK
LRFN4
LRFN3
LRFN1
LRCH3
LPO
LPIN3
LPGAT1
LPCAT4
LPCAT1
LPAR6
LPAR2
LOXL4
LOXL3
LOXL2
LOX
LONP1
LOC730183
LOC730098
LOC728485
LOC388780
LOC112268124

LOC107987373; MRPL23
LOC105376731
LOC105372824; PDXK
LOC102723713; TP53TG3F;
LOC102723655; TP53TG3D;
TP53TG3E
LOC101928120
LOC101927572
LOC100505549
LOC100289561
LOC100287036
LOC100130449
LOC100130357
LOC100129484
LOC100128108
LMX1B
LMTK2
LMO3
LMNTD1
LMNB2
LMNB1
LMNA
LMF2
LMCD1
LMBR1L
LMAN2L
LMAN2
LLPH
LLGL2
LLGL1
LKAAEAR1
LIX1L
LIPT2
LIPT1
LINS1
LINGO1
LINC02875
LINC02210-CRHR1; CRHR1
LINC02054
LINC00452
LIN9
LIN7B
LIN37
LIN28B

LIN28A
LIMS1
LIMK2
LIMK1
LIMD2
LILRB3; LOC107987425;
LOC107987462; LOC102725035
LIG3
LIG1
LIF
LIAS
LHX5
LHX4
LHX1
LHFPL5
LHFPL4
LHFPL3
LHFPL2
LHB
LGI2
LGALSL
LGALS9
LGALS3BP
LGALS3
LGALS2
LGALS14
LGALS1
LFNG
LETMD1
LETM2
LETM1
LEPROTL1
LEO1
LENG9
LENG8
LENG1
LEMD3
LEMD2
LEMD1
LEF1
LDOC1
LDLRAD3
LDHA
LDB1

LDAH
LCTL
LCT
LCORL
LCOR
LCNL1
LCN2
LCN1
LCMT1
LCLAT1
LBR
LBHD1
LAYN
LAT
LASP1
LAS1L
LARS1
LARP4B
LARP1
LARGE2
LAPTM5
LAPTM4B
LANCL2
LANCL1
LAMTOR5
LAMTOR4
LAMTOR2
LAMTOR1
LAMP5
LAMC1
LAMB4
LAMB1
LAMA5
LAMA4
LAIR2
LAIR1
LAGE3
LAD1
L3MBTL2
L3MBTL1
KY
KXD1
KTI12
KSR2

KSR1
KRTDAP
KRTCAP2
KRTAP6-3
KRTAP5-10
KRTAP5-1
KRTAP3-1
KRTAP20-2
KRTAP19-1
KRT8
KRT79
KRT78
KRT40
KRT26
KRT25
KRT23
KRT18
KRT17
KRT15
KRT10
KRR1
KRI1
KREMEN2
KRBOX4
KRBA1
KPTN
KPNB1
KPNA5
KPNA4
KPNA2
KNTC1
KNSTRN
KNOP1
KMT5C
KMT5A
KMT2D
KMT2B
KMT2A
KLRG2
KLRC2
KLK6
KLK14
KLK1
KLHL7

KLHL42
KLHL38
KLHL35
KLHL31
KLHL25
KLHL23; PHOSPHO2-KLHL23
KLHL22
KLHL18
KLHL17
KLHL12
KLHDC8B
KLHDC8A
KLHDC4
KLHDC3
KLF16
KLC3
KLC2
KLC1
KIZ
KITLG
KISS1R
KISS1
KIRREL3
KIRREL2
KIN
KIFC3
KIFC2
KIFC1
KIFBP
KIF9
KIF7
KIF5C
KIF5B
KIF5A
KIF4A
KIF3C
KIF3A
KIF2C
KIF2A
KIF26B
KIF24
KIF23
KIF22
KIF21B

KIF20B
KIF20A
KIF18B
KIF18A
KIF15
KIF14
KIF12
KIF11
KIAA1841
KIAA1614
KIAA1586
KIAA1549L
KIAA1549
KIAA1522
KIAA1328
KIAA1324
KIAA1143
KIAA0930
KIAA0895L
KIAA0753
KIAA0556
KIAA0319L
KIAA0319
KIAA0100
KHSRP
KHDRBS3
KHDRBS1
KHDC4
KHDC1
KEAP1
KDM5C
KDM5B
KDM4C
KDM3B
KDM3A
KDM2B
KDM2A
KDM1A
KDELR3
KDELR2
KDELR1
KCTD8
KCTD7
KCTD6

KCTD5
KCTD20
KCTD2
KCTD19
KCTD17
KCTD15
KCTD13
KCTD11
KCTD10
KCTD1
KCP
KCNV2
KCNV1
KCNRG
KCNQ4
KCNQ3
KCNQ2
KCNQ1
KCNN4
KCNMB4
KCNMB3
KCNK9
KCNK7
KCNK12
KCNJ9
KCNJ14
KCNJ11
KCNIP3
KCNIP2
KCNH4
KCNH3
KCNH2
KCNG3
KCNG2
KCNG1
KCNF1
KCNE5
KCNE3
KCND1
KCNC4
KCNC3
KCNC1
KCNB2
KCNAB3

KCMF1
KBTBD6
KBTBD3
KBTBD2
KAZALD1
KATNBL1
KATNB1
KATNAL2
KATNA1
KAT8
KAT7
KAT5
KAT2A
KAT14
KARS1
KANSL3
KANSL2
KANSL1
KAAG1
JUP
JUND
JTB
JSRP1
JRKL
JRK
JPT2
JPT1
JOSD2
JOSD1
JMJD6
JMJD4
JCAD
JARID2
JAML
JAM3
JAK3
JAGN1
JAG2
JAG1
JADE2
IZUMO1
IWS1
IVNS1ABP
ITPRIPL1

ITPR3
ITPKC
ITPKA
ITPA
ITM2C
ITIH6
ITGB5
ITGB4
ITGB3BP
ITGB1BP2
ITGB1BP1
ITGAX
ITGAE
ITGA6
ITGA5
ITGA3
ITGA2
ITFG2
ISYNA1
ISY1
IST1
ISM2
ISL2
ISL1
ISG20L2
ISG20
ISCU
ISCA2
ISCA1
IRX6
IRX5
IRX3
IRGQ
IRGC
IRF5
IRF3
IRF2BP2
IRF2BP1
IRAK4
IRAK2
IRAK1BP1
IRAK1
IQSEC1
IQGAP3

IQCE
IQCD
IQCC
IQCB1
IQCA1
IPO9
IPO7
IPO5
IPO4
IPO13
IPO11
IP6K2
IP6K1
INTU
INTS9
INTS8
INTS7
INTS5
INTS4
INTS3
INTS14
INTS13
INTS12
INTS11
INTS1
INSYN2A
INSM1
INSL3
INPPL1
INPP5K
INPP5J
INPP5F
INPP5E
INPP5A
INPP4A
INO80E
INO80D
INO80C
INO80B
INO80
INKA2
INKA1
INHA
ING5

ING4
ING3
ING2
INF2
INCENP
INCA1
INAVA
INAFM2
INAFM1
IMPG2
IMPDH2
IMPDH1
IMP4
IMP3
IMMT
IMMP1L
ILVBL
ILKAP
ILF3
ILF2
IL9R
IL4I1
IL3RA
IL37
IL31RA
IL2RA
IL27RA
IL1R2
IL1A
IL18BP
IL18
IL17RD
IL17RA
IL17F
IL17B
IL15RA
IL12A
IL11RA
IL11
IL10RB
IKZF4
IKBKE
IKBKB
IKBIP

IK
IGSF3
IGSF22
IGSF11
IGHMBP2
IGFL4
IGFL2
IGFBPL1
IGF2BP3
IGF2BP2
IGF2BP1
IGDCC4
IGDCC3
IGBP1
IFTAP
IFT81
IFT80
IFT74
IFT52
IFT43
IFT27
IFT22
IFT20
IFT172
IFT140
IFT122
IFRD2
IFRD1
IFNGR2
IFNAR2
IFITM3
IFITM10
IFI35
IFI30
IFI27L2
IFI27L1
IFFO2
IFFO1
IER5L
IER5
IER3IP1
IER3
IDUA
IDI2

IDH3G
IDH3B
IDE
ICMT
ICE2
ICE1
ICA1L
IBSP
IBA57
IARS1
IAH1
HYPK
HYLS1
HYI
HYAL4
HYAL3
HYAL2
HUWE1
HUS1B
HUS1
HTT
HTRA4
HTRA3
HTRA2
HTR3B
HTR3A
HTR2C
HTR1F
HTR1D
HTATSF1
HSPH1
HSPE1-MOB4
HSPE1
HSPD1
HSPBP1
HSPBAP1
HSPB11
HSPB1
HSPA6
HSPA5
HSPA4
HSPA2
HSPA1L
HSPA1B

HSPA1A
HSPA14
HSPA13
HSPA12A
HSP90B1
HSP90AB1
HSP90AA1
HSF4
HSF2BP
HSF2
HSF1
HSDL1
HSD17B7
HSD17B3
HSD17B1
HSD11B2
HSD11B1L
HSCB
HSBP1L1
HSBP1
HS6ST2
HS3ST6
HS3ST5
HS2ST1
HS1BP3
HROB
HRK
HRH3
HRCT1
HRAS
HR
HPSE2
HPS6
HPS4
HPS1
HPF1
HPDL
HPCAL1
HPCA
HOXD9
HOXD8
HOXD4
HOXD3
HOXD13

HOXD11
HOXD10
HOXD1
HOXC9
HOXC8
HOXC6
HOXC4
HOXC13
HOXC12
HOXC11
HOXC10
HOXB9
HOXB8
HOXB7
HOXB13
HOXA9
HOXA6
HOXA5
HOXA3
HOXA2
HOXA11
HOXA10
HOPX
HOOK2
HOMEZ
HOMER3
HOMER1
HNRNPUL2
HNRNPUL1
HNRNPU
HNRNPR
HNRNPM
HNRNPLL
HNRNPL
HNRNPK
HNRNPH3
HNRNPH1
HNRNPF
HNRNPDL
HNRNPD
HNRNPC
HNRNPAB
HNRNPA3
HNRNPA2B1

HNRNPA1L2
HNRNPA1
HNRNPA0
HNF1B
HMX2
HMMR
HMGXB4
HMGXB3
HMGN4
HMGN3
HMGN2
HMGN1
HMGB2
HMGB1
HMGA2
HMGA1
HMG20B
HMCES
HMBS
HM13
HLTF
HLA-A
HKDC1
HK2
HK1
HJURP
HIRA
HIPK4
HIP1R
HINT1
HINFP
HILPDA
HIKESHI
HIGD2A
HIGD1B
HIF1AN
HID1
HIC2
HHIPL2
HHEX
HGS
HGH1
HFM1
HEY1

HEXD
HEXB
HEXA
HES7
HES6
HES4
HES2
HERPUD2
HERC4
HEPACAM2
HEMK1
HELLS
HELB
HECTD2
HEBP2
HEATR6
HEATR3
HEATR1
HDLBP
HDHD5
HDGFL2
HDGF
HDDC3
HDDC2
HDAC8
HDAC7
HDAC5
HDAC4
HDAC3
HDAC2
HDAC11
HDAC10
HDAC1
HCST
HCRT
HCN2
HCFC1R1
HCFC1
HCCS
HCAR1
HBQ1
HAX1
HAVCR1
HAUS8

HAUS7
HAUS6
HAUS5
HAUS3
HAUS2
HAUS1
HAT1
HASPIN
HAS3
HARS2
HARS1
HARBI1
HAPLN3
HAPLN1
HAP1
HAGHL
HACD1
HABP4
H4C9
H4C5
H4C4
H4C3
H4C11
H4-16
H3C7
H3C4
H3C3
H3C13
H3C11
H3-5
H3-3B
H3-3A
H2BU1
H2BC9
H2BC8
H2BC6
H2BC18
H2BC15
H2BC14
H2AZ2
H2AZ1
H2AX
H2AW
H2AJ

H2AC7
H2AC4
H2AC21
H2AC20
H2AC17
H2AC16
H2AC14
H2AC12
H1-5
H1-4
H1-3
H1-10
GZF1
GYS1
GYPC
GYG1
GXYLT2
GUK1
GUF1
GUCY2D
GUCA2A
GTSF1L
GTSE1
GTPBP8
GTPBP6
GTPBP4
GTPBP3
GTPBP2
GTPBP1
GTF3C6
GTF3C5
GTF3C4
GTF3C3
GTF3C2
GTF3C1
GTF3A
GTF2IRD1
GTF2H5
GTF2H4
GTF2H3
GTF2H2C
GTF2H2; GTF2H2C
GTF2H1
GTF2F2

GTF2F1
GTF2E2
GTF2E1
GTF2B
GTF2A2
GTDC1
GSTP1
GSTO2
GSTCD
GSS
GSR
GSPT1
GSK3A
GSE1
GSDME
GSDMD
GSDMB
GSC
GRWD1
GRSF1
GRPEL2
GRN
GRM4
GRK7
GRK6
GRK4
GRK3
GRK2
GRIPAP1
GRINA
GRIN3B
GRIN2D
GRIN2A
GRIN1
GRIK5
GRIK4
GRID2IP
GRID2
GRIA4
GRIA2
GRB7
GRB2
GRASP
GRAMD2A

GRAMD1A
GPX8
GPX7
GPX4
GPX1
GPSM2
GPSM1
GPS2
GPS1
GPRIN1
GPRC6A
GPRC5D
GPR89B
GPR89A
GPR85
GPR84
GPR63
GPR37L1
GPR35
GPR3
GPR22
GPR21; RABGAP1
GPR19
GPR173
GPR161
GPR160
GPR157
GPR156
GPR137C
GPR137
GPR119
GPR108
GPR107
GPN3
GPN2
GPN1
GPM6B
GPKOW
GPI
GPD2
GPD1L
GPCPD1
GPC3
GPC2

GPBP1
GPATCH8
GPATCH4
GPATCH3
GPATCH2L
GPATCH2
GPATCH1
GPAT4
GPAT2
GPANK1
GPALPP1
GPAA1
GOSR2
GORASP2
GORASP1
GORAB
GON7
GON4L
GOLT1B
GOLPH3L
GOLM1
GOLGA8B
GOLGA8A
GOLGA7B
GOLGA6L9
GOLGA6L7
GOLGA6L4
GOLGA6L2
GOLGA6L10; GOLGA6L19
GOLGA3
GOLGA2
GNS
GNRHR
GNRH1
GNPDA2
GNPDA1
GNPAT
GNL3
GNL2
GNL1
GNG8
GNG5
GNG4
GNG13

GNB3
GNB2
GNB1L
GNB1
GNAZ
GNAS
GNAL
GNAI3
GNA15
GNA12
GNA11
GMPS
GMPR2
GMPPB
GMPPA
GMNN
GMIP
GMFG
GMFB
GMEB2
GMEB1
GMDS
GMCL1
GM2A
GLTP
GLT8D1
GLS
GLRX3
GLRX2
GLP1R
GLO1
GLMP
GLMN
GLIS2
GLIPR2
GLI4
GLI1
GLG1
GLE1
GLDN
GLB1L
GLB1
GLA
GKN1

GKAP1
GK5
GJD4
GJC1
GJB6
GJA3
GIT2
GIT1
GIPR
GIPC1
GIP
GINS4
GINS3
GINS2
GINS1
GIGYF2
GIGYF1
GID8
GHRL
GHDC
GGTLC3
GGTLC1
GGT7
GGPS1
GGNBP2
GGN
GGCT
GGA3
GGA2
GGA1
GFY
GFPT1
GFI1
GFER
GET4
GET3
GEMIN8
GEMIN7
GEMIN6
GEMIN5
GEMIN4
GEMIN2
GDPGP1
GDPD5

GDPD3
GDPD2
GDPD1
GDI2
GDI1
GDF15
GDF11
GDF1; CERS1
GDAP1L1
GDAP1
GCNT7
GCNT3
GCNA
GCN1
GCM1
GCFC2
GBX2
GBP2
GBGT1
GBF1
GBA2
GBA
GATD1
GATC
GATAD2B
GATAD2A
GATAD1
GAST
GAS8
GAS7
GAS2L3
GAS2L1
GART
GARS1
GAREM2
GAR1
GAPVD1
GAPDHS
GAPDH
GAP43
GAN
GALR2
GALNT6
GALNT5

GALNT10
GALE
GAL3ST4
GAL3ST3
GAL3ST1
GAL
GAK
GADD45GIP1
GAD2
GAD1
GABRR3
GABRR1
GABRQ
GABRE
GABRD
GABRB1
GABPB2
GABPB1
GABBR1
GABARAPL2
GABARAP
GAB2
G6PD
G6PC3
G3BP1
G2E3
FZR1
FZD9
FZD7
FZD6
FZD2
FXYD5
FXYD3
FXYD2
FXR2
FXR1
FUT9
FUT7
FUT4
FUT3
FUT2
FUT11
FUT1
FUS

FUNDC2
FUNDC1
FUCA2
FUBP1
FTSJ3
FTSJ1
FTL
FTH1
FSTL4
FSTL3
FSD1L
FSCN3
FSCN2
FSCN1
FRS3
FRRS1L
FRMD8
FRMD5
FRMD1
FRG1
FRAT2
FRAS1
FRA10AC1
FOXS1
FOXRED2
FOXP4
FOXP1
FOXN1
FOXM1
FOXL1
FOXK2
FOXK1
FOXJ3
FOXJ1
FOXH1
FOXG1
FOXF2
FOXE3
FOXE1
FOXD4L1
FOXD4
FOXD2
FOXD1
FOXC2

FOLR1
FOCAD
FNTA
FNDC8
FNDC3B
FNDC11
FNDC10
FNBP4
FNBP1L
FNBP1
FN3KRP
FMNL3
FMNL2
FMNL1
FLYWCH2
FLYWCH1
FLVCR1
FLT3LG
FLRT1
FLOT2
FLOT1
FLNC
FLNB
FLNA
FLJ45513
FLII
FLCN
FLAD1
FLACC1
FKRP
FKBPL
FKBP9
FKBP8
FKBP6
FKBP4
FKBP3
FKBP2
FKBP1A
FKBP15
FKBP14
FKBP11
FKBP10
FJX1
FIZ1

FIS1
FIP1L1
FIGNL2
FIGNL1
FIG4
FIBP
FIBCD1
FHOD1
FHL3
FHIT
FHDC1
FGFR4
FGFR3
FGFR1OP2
FGFBP3
FGF8
FGF17
FGF11
FGD6
FGD3
FGD1
FEZ2
FEV
FERMT3
FERMT1
FER1L6
FER1L5
FER
FEN1
FDXACB1
FDX2
FDPS
FDCSP
FCSK
FCHSD1
FCHO1
FCGR2A; FCGR2C
FCGR1B
FCGR1A
FCGBP
FCER1G
FBXW9
FBXW8
FBXW4

FBXW12
FBXO9
FBXO5
FBXO46
FBXO45
FBXO44
FBXO43
FBXO42
FBXO41
FBXO4
FBXO32
FBXO30
FBXO22
FBXO17
FBXO10
FBXL8
FBXL7
FBXL6
FBXL22
FBXL2
FBXL19
FBXL18
FBXL15
FBXL13
FBXL12
FBRSL1
FBRS
FBN3
FBLN1
FBLL1
FBLIM1
FBL
FBH1
FBF1
FAXC
FAU
FATE1
FASTKD5
FASTKD3
FASTKD2
FASTKD1
FASTK
FARSB
FARSA

FARS2
FANK1
FANCM
FANCL
FANCI
FANCG
FANCF
FANCE
FANCD2OS
FANCD2
FANCB
FANCA
FAM98C
FAM98B
FAM98A
FAM91A1
FAM90A1
FAM89B
FAM89A
FAM86C1
FAM83H
FAM83G
FAM83D
FAM81B
FAM81A
FAM78B
FAM78A
FAM72D
FAM72C
FAM72B
FAM72A
FAM71F2
FAM71F1
FAM71E1
FAM71D
FAM53C
FAM53B
FAM50A
FAM3B
FAM3A
FAM32A
FAM25E
FAM24B
FAM241B

FAM234A
FAM229A
FAM228B
FAM227A
FAM222B
FAM222A
FAM220A
FAM219B
FAM219A
FAM217B
FAM217A
FAM216A
FAM214B
FAM20B
FAM209B
FAM209A
FAM207A
FAM204A
FAM200B
FAM200A
FAM199X
FAM193B
FAM193A
FAM189B
FAM186A
FAM184B
FAM178B
FAM177B
FAM174C
FAM171B
FAM171A2
FAM168B
FAM166C
FAM160B2
FAM156A
FAM155B
FAM149B1
FAM136A
FAM135A
FAM133B
FAM131C
FAM131A
FAM122B
FAM120AOS

FAM118B
FAM118A
FAM117B
FAM111B
FAM111A
FAM110A
FAM104B
FAM104A
FAM102B
FAIM
FAF2
FAF1
FADS3
FADD
FABP9
FABP7
FABP6
FABP5
FAAP24
FAAP20
FAAP100
F2RL3
EZR
EZH2
EZH1
EYA3
EYA1
EXTL3
EXTL2
EXTL1
EXT2
EXT1
EXOSC9
EXOSC8
EXOSC7
EXOSC6
EXOSC5
EXOSC4
EXOSC3
EXOSC2
EXOSC10
EXOSC1
EXOG
EXOC7

EXOC6B
EXOC6
EXOC4
EXOC3
EXOC2
EXOC1
EXO5
EXO1
EXD3
EXD1
EWSR1
EVX1
EVPL
EVI5L
EVC2
EVA1B
ETV6
ETV5
ETV4
ETV3L
ETV3
ETV2
ETNK1
ETF1
ETAA1
ESYT3
ESS2
ESRRA
ESPL1
ESM1
ESF1
ESCO2
ESCO1
ERVMER34-1
ERV3-1-ZNF117; ZNF117
ERV3-1
ERP44
ERP29
ERP27
ERO1A
ERMP1
ERLEC1
ERICH4
ERICH2

ERICH1
ERI3
ERI1
ERH
ERGIC3
ERGIC2
ERFL
ERFE
ERF
ERCC8
ERCC6L
ERCC5; BIVM-ERCC5
ERCC3
ERCC2
ERCC1
ERC2
ERBB3
ERAS
ERAL1
EQTN
EPS8L3
EPS8L2
EPS8L1
EPS15L1
EPRS1
EPPK1
EPOR
EPN3
EPN2
EPN1
EPHX4
EPHB3
EPHB2
EPHA8
EPHA5
EPHA10
EPDR1
EPCAM
EPC1
EPB41L2
EP400
EOLA2
EOGT
ENY2

ENTR1
ENTPD6
ENTPD3
ENTPD2
ENTPD1
ENSA
ENOPH1
ENO4
ENO2
ENO1
ENKD1
ENGASE
ENAH
EN2
EN1
EMP3
EML6
EML4
EML3
EML2
EMILIN2
EMG1
EME2
EME1
EMD
EMC9
EMC8
EMC7
EMC4
EMC3
EMC2
EMC10
ELP6
ELP5
ELP4
ELP2
ELP1
ELOVL7
ELOVL4
ELOVL1
ELOF1
ELOC
ELOB
ELOA2

ELMOD3
ELMOD2
ELMO3
ELMO2
ELL3
ELL
ELK1
ELF4
ELF3
ELAVL3
ELAVL1
ELAC2
EIPR1
EIF6
EIF5B
EIF5A2
EIF5A
EIF4G2
EIF4ENIF1
EIF4EBP1
EIF4E2
EIF4E
EIF4B
EIF4A3
EIF4A2
EIF4A1
EIF3M
EIF3K
EIF3J
EIF3I
EIF3H
EIF3G
EIF3F
EIF3E
EIF3D
EIF3CL
EIF3B
EIF2S3B
EIF2S3
EIF2S2
EIF2S1
EIF2D
EIF2B5
EIF2B4

EIF2B3
EIF2B2
EIF2B1
EIF2AK1
EIF2A
EIF1B
EIF1AD
EIF1
EID3
EID2B
EID2
EHMT2
EHMT1
EHD4
EHD2
EHD1
EHBP1L1
EGLN3
EGLN2
EGFL8
EGFL7
EGFL6
EFTUD2
EFR3B
EFNA5
EFNA4
EFNA3
EFNA1
EFL1
EFHD2
EFHC1
EFCAB8
EFCAB7
EFCAB5
EFCAB3
EFCAB2
EFCAB11
EFCAB10
EEPD1
EEFSEC
EEF2KMT
EEF2
EEF1G
EEF1E1

EEF1D
EEF1B2
EEF1AKNMT
EEF1AKMT3
EEF1AKMT2
EEF1AKMT1
EEF1A1
EED
EDRF1
EDIL3
EDF1
EDEM2; MMP24-AS1-EDEM2
EDDM13
EDC3
EDARADD
ECT2
ECSIT
ECPAS
ECE2
ECD
EBNA1BP2
EBF1
EBAG9
EAF2
E4F1
E2F8
E2F7
E2F6
E2F5
E2F4
E2F3
E2F2
E2F1
DZIP3
DZIP1L
DZANK1
DYRK4
DYRK2
DYRK1B
DYNLT1
DYNLRB1
DYNLL1
DYNC2LI1
DYNC1LI1

DYNC1I2
DYNC1H1
DYM
DYDC2
DYDC1
DXO
DVL3
DVL2
DVL1
DUT
DUSP9
DUSP8
DUSP7
DUSP4
DUSP28
DUSP23
DUSP22
DUSP18
DUSP15
DUSP14
DUSP13
DUSP12
DUSP11
DUS4L
DUS3L
DUS2
DUS1L
DUOX1
DTYMK
DTX3
DTX2
DTWD1
DTNBP1
DTNB
DTL
DTD1
DSTYK
DSTN
DSN1
DSCC1
DRP2
DROSHA
DRICH1
DRG2

DRG1
DRD4
DRD2
DRC3
DRC1
DRAXIN
DRAP1
DRAM2
DRAM1
DR1
DQX1
DPYSL5
DPYSL4
DPY30
DPY19L3
DPPA3
DPP9
DPP8
DPP6
DPP3
DPM3
DPM2
DPM1
DPH7
DPH6
DPH5
DPH3
DPH2
DPF2
DPF1
DPCD
DPAGT1
DOT1L
DONSON
DOLK
DOK3
DOK1
DOHH
DOCK6
DOCK3
DNTTIP2
DNTTIP1
DNPH1
DNPEP

DNMT3B
DNMT3A
DNMT1
DNM2
DNM1L
DNM1
DNLZ
DNHD1
DNER
DNASE1L2
DNASE1
DNAL4
DNAL1
DNAJC9
DNAJC8
DNAJC7
DNAJC6
DNAJC5G
DNAJC5
DNAJC4
DNAJC30
DNAJC21
DNAJC2
DNAJC18
DNAJC17
DNAJC10
DNAJC1
DNAJB7
DNAJB6
DNAJB5
DNAJB2
DNAJB13
DNAJB12
DNAJB11
DNAJB1
DNAJA4
DNAH8
DNAH3
DNAH17
DNAH14
DNAH12
DNAAF5
DNAAF4
DNA2

DMWD
DMTF1
DMRT3
DMRT2
DMRT1
DMPK
DMP1
DMKN
DMC1
DMBX1
DMBT1
DMAP1
DMAC2L
DMAC2
DLX6
DLX5
DLX4
DLX3
DLX2
DLX1
DLL4
DLL3
DLK2
DLGAP5
DLGAP4
DLGAP3
DLG5
DLG4
DLG3
DLAT
DKKL1
DKK1
DKC1
DISP3
DIS3L2
DIRAS1
DIPK2B
DIPK1B
DIP2A
DIMT1
DIDO1
DIAPH3
DHX9
DHX8

DHX57
DHX38
DHX37
DHX35
DHX34
DHX33
DHX30
DHX16
DHX15
DHRS7B
DHRS13
DHRS11
DHPS
DHDH
DGUOK
DGKZ
DGKQ
DGKK
DGKI
DGKH
DGKD
DGKA
DGCR8
DGCR2
DGAT1
DFFB
DFFA
DEUP1
DESI2
DESI1
DERL2
DERL1
DEPDC4
DEPDC1B
DEPDC1
DENR
DENND6B
DENND6A
DENND5A
DENND4B
DENND3
DENND2D
DENND1A
DENND10

DELEC1
DEK
DEGS1
DEFB126
DEFA6
DEF8
DEF6
DEDD2
DEDD
DEAF1
DDX59
DDX56
DDX55
DDX54
DDX52
DDX51
DDX50
DDX5
DDX49
DDX47
DDX46
DDX42
DDX41
DDX4
DDX39B
DDX39A
DDX31
DDX28
DDX27
DDX24
DDX23
DDX21
DDX20
DDX19A
DDX18
DDX17
DDX11
DDX10
DDX1
DDRGK1
DDR1
DDOST
DDN
DDIT3

DDIAS
DDHD1
DDB1
DDAH2
DDA1
DCUN1D5
DCUN1D2
DCTPP1
DCTN5
DCTN4
DCTN3
DCTN2
DCTN1
DCP2
DCP1B
DCP1A
DCLRE1C
DCLRE1B
DCDC2B
DCDC2
DCAF7
DCAF4L1
DCAF4
DCAF17
DCAF16
DCAF15
DCAF13
DCAF12
DBR1
DBNL
DBNDD2
DBN1
DBI
DBF4B
DBF4
DAZAP1
DAXX
DAW1
DARS2
DARS1
DAPK3
DAPK2
DAP3
DAP

DAND5
DALRD3
DAGLB
DAGLA
DAD1
DACH2
CZIB
CYTH4
CYTH2
CYSTM1
CYSRT1
CYRIB
CYREN
CYP2W1
CYP2R1
CYP27B1
CYP26B1
CYP24A1
CYP20A1
CYP19A1
CYHR1
CYCS
CYC1
CYBC1
CYBA
CYB5RL
CYB5R3
CYB5R1
CYB5B
CYB561D2
CYB561D1
CXXC5
CXXC1
CXorf65
CXorf56
CXorf38
CXCR4
CXCL5
CXCL3
CXCL17
CWF19L1
CWC27
CWC25
CWC22

CWC15
CUZD1
CUX1
CUTC
CUTA
CUL9
CUL7
CUL4B
CUL2
CUL1
CUEDC2
CUEDC1
CUBN
CTXN1
CTU2
CTU1
CTTNBP2NL
CTTN
CTSV
CTSK
CTSC
CTSA
CTRL
CTRC
CTPS2
CTNS
CTND2
CTNNBL1
CTNNBIP1
CTNNAL1
CTNNA1
CTLA4
CTHRC1
CTDSPL2
CTDSPL
CTDSP2
CTDSP1
CTDP1
CTDNEP1
CTCF
CTBP2
CTBP1
CT83
CT55

CT45A10
CSTL1
CSTF3
CSTF2
CSTF1
CSTB
CST9L
CST8
CST6
CST3
CST2
CST1
CSRNP2
CSPP1
CSPG5
CSPG4
CSNK2B
CSNK2A2
CSNK2A1
CSNK1G2
CSNK1G1
CSNK1E
CSNK1D
CSKMT
CSK
CSF1
CSE1L
CS
CRYZL1
CRYGS
CRYBG2
CRYBB3
CRYBB2
CRYBA1
CRTC3
CRTC2
CRTC1
CRTAP
CROCC
CRNKL1
CRNDE
CRMP1
CRLF3
CRLF1

CRKL
CRISP2
CRIPT
CRIP1
CRHR2
CRELD2
CRELD1
CREG2
CREB5
CREB3L4
CREB3L2
CREB3
CREB1
CRCP
CRB2
CRAMP1
CRACR2B
CRACDL
CRABP2
CPXM1
CPVL
CPT1C
CPT1B
CPSF7
CPSF6
CPSF4L
CPSF4
CPSF3
CPSF2
CPSF1
CPNE9
CPNE7
CPNE5
CPNE2
CPNE1
CPLX2
CPLX1
CPEB1
CPD
CPA6
CPA2
COX8A
COX7C
COX7B

COX7A2L
COX7A2
COX6C
COX6B2
COX6B1
COX6A1
COX5B
COX5A
COX4I2
COX4I1
COX20
COX19
COX17
COX16
COX14
COX11
COX10
CORO7
CORO6
CORO2A
CORO1C
CORO1B
COQ8B
COQ7
COQ5
COQ4
COQ3
COPZ1
COPS9
COPS8
COPS7B
COPS7A
COPS6
COPS5
COPS4
COPS3
COPS2
COPRS
COPG2
COPG1
COPE
COPB2
COPB1
COPA

COP1
COMTD1
COMP
COMMD9
COMMD8
COMMD7
COMMD6
COMMD5
COMMD4
COMMD3
COMMD2
COMMD1
COLQ
COLGALT2
COLGALT1
COLEC12
COLCA2
COLCA1
COL9A2
COL9A1
COL7A1
COL5A2
COL4A2
COL4A1
COL2A1
COL27A1
COL22A1
COL11A2
COL11A1
COIL
COG8
COG7
COG5
COG4
COG2
COG1
COCH
COASY
COA8
COA7
COA6
COA5
COA4
COA3

COA1
CNTROB
CNTRL
CNTNAP5
CNTNAP1
CNTD1
CNPY4
CNPY3
CNPY2
CNPPD1
CNP
CNOT9
CNOT8
CNOT6
CNOT4
CNOT3
CNOT2
CNOT11
CNOT10
CNNM4
CNNM2
CNNM1
CNN2
CNIH4
CNIH3
CNIH2
CNGB1
CNGA3
CNFN
CNBD2
CMTR1
CMTM7
CMTM4
CMTM3
CMTM1
CMSS1
CMIP
CMC2
CMC1
CLUL1
CLUAP1
CLTB
CLTA
CLSTN3

CLSTN1
CLSPN
CLPTM1L
CLPTM1
CLPSL2
CLPSL1
CLPP
CLP1
CLNS1A
CLN6
CLN3
CLLU1-AS1
CLK4
CLK3
CLK2
CLK1
CLIP2
CLIC3
CLIC1
CLHC1
CLEC4A
CLEC2L
CLEC2D
CLEC18C
CLEC18B
CLEC18A
CLDND2
CLDN6
CLDN18
CLCNKB
CLCNKA
CLCN7
CLCN6
CLCN2
CLCF1
CLBA1
CLASRP
CKS2
CKS1B
CKMT1B
CKMT1A
CKM
CKLF-CMTM1
CKLF

CKB
CKAP5
CKAP4
CKAP2L
CKAP2
CIZ1
CITED4
CITED1
CIT
CISD1
CIRBP
CIR1
CIP2A
CINP
CILP2
CIC
CIB2
CIB1
CIART
CIAPIN1
CIAO3
CIAO2B
CIAO1
CHURC1-FNTB; FNTB
CHUK
CHTOP
CHTF18
CHST6
CHST2
CHST14
CHST12
CHST11
CHST10
CHST1
CHRNG
CHRNB2
CHRNA9
CHRNA7
CHRNA5
CHRNA10
CHRNA1
CHRM1
CHRD
CHRAC1

CHPF2
CHORDC1
CHMP7
CHMP6
CHMP4C
CHMP4B
CHMP4A
CHMP3
CHMP2B
CHMP2A
CHMP1A
CHML
CHKB
CHKA
CHID1
CHI3L2
CHGB
CHGA
CHFR
CHERP
CHEK2
CHEK1
CHD8
CHD7
CHD5
CHD4
CHD3
CHD2
CHD1L
CHCHD7
CHCHD6
CHCHD5
CHCHD4
CHCHD3
CHCHD2
CHCHD1
CHAF1B
CHAF1A
CGRRF1
CGREF1
CGGBP1
CGB7
CGAS
CFLAR

CFL1
CFDP1
CFAP92
CFAP77
CFAP73
CFAP65
CFAP61
CFAP53
CFAP45
CFAP44
CFAP43
CFAP410
CFAP36
CFAP300
CFAP298
CFAP157
CFAP100
CETN3
CETN2
CERS5
CERS3
CERK
CERCAM
CER1
CEP95
CEP89
CEP85
CEP83
CEP78
CEP72
CEP68
CEP63
CEP55
CEP41
CEP350
CEP295NL
CEP295
CEP290
CEP250
CEP192
CEP19
CEP170B
CEP170
CEP164

CEP162
CEP152
CEP135
CEP131
CEP126
CEP112
CENPX
CENPW
CENPV
CENPU
CENPT
CENPS
CENPQ
CENPP
CENPO
CENPN
CENPM
CENPL
CENPK
CENPJ
CENPI
CENPH
CENPF
CENPE
CENPB
CENPA
CEND1
CELSR3
CELF4
CELF3
CELA3B
CEL
CEBPZOS
CEBPZ
CEBPG
CEACAM7
CEACAM19
CDYL
CDX2
CDV3
CDT1
CDS2
CDRT4
CDR2L

CDPF1
CDON
CDKN3
CDKN2D
CDKN2C
CDKN2B
CDKN2AIPNL
CDKN2A
CDKL4
CDKL3
CDKAL1
CDK9
CDK8
CDK7
CDK5RAP3
CDK5RAP2
CDK5RAP1
CDK5R2
CDK5R1
CDK5
CDK4
CDK3
CDK2AP2
CDK2AP1
CDK20
CDK2
CDK19
CDK16
CDK13
CDK12
CDK11B
CDK11A
CDK10
CDK1
CDIPT
CDHR4
CDHR3
CDH9
CDH8
CDH6
CDH3
CDH24
CDH18
CDH16

CDH10
CDCP2
CDCP1
CDCA8
CDCA7L
CDCA7
CDCA5
CDCA4
CDCA3
CDCA2
CDC7
CDC6
CDC45
CDC42SE2
CDC42SE1
CDC42EP5
CDC42EP4
CDC42EP2
CDC42EP1
CDC42
CDC37
CDC34
CDC27
CDC26
CDC25C
CDC25B
CDC25A
CDC23
CDC20B
CDC20
CDC16
CDC123
CDAN1
CD99
CD83
CD79B
CD72
CD70
CD7
CD68
CD63
CD58
CD3EAP
CD3D

CD34
CD320
CD300LF
CD2BP2
CD2AP
CD276
CD248
CD24
CD200
CD1A
CD177
CD151
CD101
CCZ1B
CCZ1
CCT8
CCT7
CCT6A
CCT5
CCT4
CCT3
CCT2
CCSAP
CCRL2
CCR3
CCR10
CCNY
CCNT2
CCNT1
CCNQ
CCNO
CCNL2
CCNL1
CCNJL
CCNJ
CCNI2
CCNH
CCNG2
CCNF
CCNE2
CCNE1
CCND3
CCND2
CCNB3

CCNB2
CCNB1
CCNA2
CCM2
CCL26
CCL20
CCKBR
CCIN
CCHCR1
CCER2
CCDC97
CCDC96
CCDC93
CCDC92
CCDC91
CCDC9
CCDC88C
CCDC88B
CCDC88A
CCDC86
CCDC85B
CCDC84
CCDC81
CCDC78
CCDC77
CCDC74B
CCDC74A
CCDC73
CCDC71
CCDC66
CCDC65
CCDC61
CCDC6
CCDC59
CCDC58
CCDC57
CCDC51
CCDC50
CCDC43
CCDC40
CCDC36
CCDC34
CCDC32
CCDC30

CCDC28B
CCDC28A-AS1
CCDC24
CCDC22
CCDC200
CCDC192
CCDC191
CCDC189
CCDC188
CCDC185
CCDC183
CCDC181
CCDC180
CCDC18
CCDC174
CCDC17
CCDC167
CCDC166
CCDC163
CCDC159
CCDC157
CCDC154
CCDC153
CCDC150
CCDC149
CCDC148
CCDC142
CCDC14
CCDC138
CCDC137
CCDC136
CCDC134
CCDC130
CCDC127
CCDC124
CCDC122
CCDC120
CCDC12
CCDC116
CCDC115
CCDC114
CCDC112
CCDC107
CCDC102B

CCDC102A
CCAR2
CCAR1
CC2D2A
CC2D1B
CC2D1A
CBY2
CBY1
CBX8
CBX6
CBX5
CBX4
CBX3
CBX2
CBX1
CBWD6
CBWD5
CBWD3; CBWD5
CBWD2
CBWD1
CBR3
CBLL1
CBL
CBFB
CBFA2T2
CBARP
CAVIN4
CAVIN3
CATSPERD
CATSPER3
CATSPER2
CATSPER1
CASTOR3
CASQ1
CASP8
CASP6
CASP5
CASP4
CASP3
CASP2
CASKIN2
CASKIN1
CASC3
CARS2

CARS1
CARMIL2
CARM1
CARD9
CARD8
CARD19
CARD14
CARD10
CAPZB
CAPZA2
CAPZA1
CAPSL
CAPS
CAPRIN2
CAPRIN1
CAPNS1
CAPN9
CAPN8
CAPN2
CAPN15
CAPN12
CAPN10
CAPN1
CAPG
CAP1
CANX
CANT1
CAND1
CAMTA1
CAMSAP3
CAMSAP2
CAMSAP1
CAMLG
CAMKV
CAMKMT
CAMKK2
CAMKK1
CAMK2G
CALY
CALU
CALR
CALML6
CALM3
CALM2

CALHM3
CALB2
CAGE1
CADPS
CAD
CACYBP
CACUL1
CACTIN
CACNG8
CACNG4
CACNB4
CACNB3
CACNB1
CACNA2D4
CACNA2D3
CACNA2D2
CACNA1G
CACNA1F
CACNA1E
CACNA1B
CACNA1A
CACFD1
CABYR
CABP7
CABP1
CABLES2
CABIN1
CAB39L
CA9
CA7
CA12
CA11
C9orf85
C9orf50
C9orf40
C9orf163
C9orf16
C9orf153
C9orf139
C9orf116
C8orf87
C8orf82
C8orf76
C8orf58

C8orf44
C8orf33
C7orf61
C7orf57
C7orf50
C7orf31
C7orf26
C7orf25
C6orf89
C6orf62
C6orf52
C6orf47
C6orf226
C6orf223
C6orf163
C6orf136
C6orf132
C5orf66
C5orf63
C5orf47
C5orf46
C5orf34
C5orf22
C4orf51
C4orf48
C4orf47
C4orf46
C3orf86
C3orf62
C3orf52
C3orf38
C3orf35
C3orf33
C3orf22
C3orf14
C2orf92
C2orf76
C2orf68
C2orf66
C2orf50
C2orf49
C2orf15
C2CD6
C2CD5

C2CD3
C2CD2L
C22orf46
C22orf42
C22orf39
C22orf34
C22orf24
C22orf23
C21orf58
C20orf96
C20orf27
C20orf204
C20orf203
C20orf202
C20orf197
C20orf194
C20orf144
C1QTNF8
C1QTNF6
C1QTNF12
C1QL4
C1QBP
C1orf56
C1orf54
C1orf52
C1orf50
C1orf43
C1orf35
C1orf229
C1orf216
C1orf198
C1orf194
C1orf174
C1orf159
C1orf146
C1orf131
C1orf127
C1orf122
C1orf116
C1orf112
C1orf109
C1orf100
C1GALT1
C19orf84

C19orf81
C19orf67
C19orf57
C19orf54
C19orf53
C19orf48
C19orf47
C19orf44
C19orf33
C19orf25
C19orf18
C18orf54
C18orf21
C17orf99
C17orf80
C17orf75
C17orf58
C17orf50
C17orf49
C17orf100
C16orf91
C16orf90
C16orf74
C15orf65
C15orf61
C15orf56
C15orf48
C15orf41
C15orf40
C15orf39
C14orf93
C14orf39
C14orf119
C12orf76
C12orf75
C12orf73
C12orf65
C12orf57
C12orf56
C12orf49
C12orf45
C12orf43
C12orf4
C11orf98

C11orf95
C11orf91
C11orf88
C11orf80
C11orf68
C11orf58
C11orf49
C11orf45
C11orf16
C10orf95
C10orf88
C10orf62
C10orf55
C10orf126
BZW2
BYSL
BUD31
BUD23
BUD13
BUB3
BUB1B
BUB1
BTRC
BTNL10
BTN2A1
BTN1A1
BTG3
BTG1
BTF3L4
BTF3
BTBD9
BTBD7
BTBD3
BTBD2
BTBD17
BTBD10
BTBD1
BSPRY
BSND
BSG
BRSK2
BRSK1
BRS3
BRPF1

BROX
BRMS1
BRK1
BRIX1
BRICD5
BRI3BP
BRI3
BRF1
BRD9
BRD8
BRD7
BRD4
BRD3OS
BRD2
BRD1
BRCA2
BRCA1
BRAT1
BRAP
BRAF
BPNT1
BPIFB6
BPIFB4
BPIFB2
BPIFB1
BPIFA2
BPIFA1
BPGM
BORCS8
BORCS6
BORCS5
BORA
BOP1
BOLL
BOLA3
BOLA2-SMG1P6
BOLA2B
BOLA1
BOD1
BNIPL
BNIP1
BMT2
BMS1
BMPR1A

BMP8B
BMP8A
BMP7
BMF
BLZF1
BLVRA
BLOC1S5
BLOC1S4
BLOC1S3
BLOC1S2
BLOC1S1
BLMH
BLM
BIRC7
BIRC5
BIN1
BIK
BID
BICRA
BICDL1
BICD2
BICD1
BICC1
BHMG1
BGLAP
BFSP2
BFSP1
BFAR
BEX4
BEX3
BEX2
BET1L
BET1
BEST4
BEST3
BEST1
BEND3
BECN2
BDKRB1
BCS1L
BCR
BCORL1
BCLAF3
BCLAF1

BCL9
BCL7C
BCL7B
BCL7A
BCL6
BCL3
BCL2L2-PABPN1
BCL2L2
BCL2L15
BCL2L14
BCL2L12
BCL2L1
BCL11A
BCL10
BCDIN3D
BCCIP
BCAT1
BCAS4
BCAS1
BCAR1
BCAP31
BCAP29
BCAN
BCAM
BBS7
BBS4
BBS1
BBIP1
BBC3
BAZ2A
BAZ1B
BAZ1A
BAX
BATF3
BATF
BARX2
BARHL1
BARD1
BAP1
BANP
BANF2
BANF1
BAMBI
BAK1

BAIAP2L2
BAIAP2L1
BAHD1
BAG6
BAG5
BAG3
BAG2
BAG1
BAD
BABAM2
BABAM1
B9D2
B9D1
B4GALT7
B4GALT5
B4GALT4
B4GALT3
B4GALT2
B4GALNT4
B4GALNT1
B3GNTL1
B3GNT8
B3GNT4
B3GNT3
B3GAT3
B3GALT6
B3GALT4
B3GALNT1
AZIN1
AXIN1
AXDND1
AVPR1B
AVL9
AVEN
AURKC
AURKB
AURKAIP1
AURKA
AUP1
AUNIP
ATXN7L3B
ATXN7L3
ATXN7L2
ATXN2L

ATXN2
ATXN10
ATRAID
ATR
ATPSCKMT
ATPAF2
ATP9A
ATP8A2
ATP6V1H
ATP6V1G3
ATP6V1G1
ATP6V1FNB
ATP6V1F
ATP6V1E2
ATP6V1E1
ATP6V1D
ATP6V1C2
ATP6V1C1
ATP6V1B1
ATP6V0E1
ATP6V0D2
ATP6V0D1
ATP6V0B
ATP6V0A4
ATP6V0A2
ATP6V0A1
ATP6AP2
ATP6AP1L
ATP6AP1
ATP5PO
ATP5PD
ATP5PB
ATP5MPL
ATP5MG
ATP5MF-PTCD1
ATP5MF
ATP5ME
ATP5MD
ATP5MC3
ATP5MC2
ATP5MC1
ATP5F1E
ATP5F1D
ATP5F1C

ATP5F1B
ATP2C2
ATP2B1
ATP2A1
ATP1B3
ATP1B1
ATP1A4
ATP1A1
ATP13A2
ATP13A1
ATN1
ATIC
ATG9B
ATG9A
ATG7
ATG4D
ATG4B
ATG3
ATG16L2
ATG16L1
ATG14
ATG13
ATG12
ATG101
ATG10
ATF7; NPFF
ATF7
ATF6B
ATF4
ATF2
ATCAY
ATAT1
ATAD5
ATAD3B
ATAD3A
ATAD2
ATAD1
ASXL1
ASTE1
ASRGL1
ASPSCR1
ASPM
ASPHD2
ASPHD1

ASNSD1
ASNS
ASMT
ASIC4
ASIC3
ASIC1
ASF1B
ASF1A
ASCL5
ASCL2
ASCC2
ASCC1
ASB6
ASB3
ASB16
ASB14
ASB1
ASAP1
ARVCF
ARV1
ARTN
ART3
ARSI
ARSG
ARRDC5
ARRDC2
ARRDC1
ARRB2
ARRB1
ARR3
ARPP19
ARPIN
ARPC5L
ARPC5
ARPC4
ARPC3
ARPC2
ARPC1B
ARPC1A
ARNTL2
ARNT2
ARMH3
ARMC9
ARMC8

ARMC7
ARMC3
ARMC2
ARMC12
ARMC10
ARMC1
ARL9
ARL8B
ARL8A
ARL6IP6
ARL6IP4
ARL4C
ARL4A
ARL3
ARL2
ARL17A
ARL16
ARL14EP
ARL10
ARL1
ARIH2OS
ARIH2
ARID5B
ARID5A
ARID4B
ARID3B
ARID3A
ARID2
ARHGEF7
ARHGEF40
ARHGEF39
ARHGEF38
ARHGEF33
ARHGEF3
ARHGEF25
ARHGEF2
ARHGEF19
ARHGEF11
ARHGEF1
ARHGDIG
ARHGDIB
ARHGDIA
ARHGAP45
ARHGAP40

ARHGAP4
ARHGAP39
ARHGAP33
ARHGAP27
ARHGAP26
ARHGAP22
ARHGAP19
ARHGAP18
ARHGAP17
ARHGAP12
ARHGAP11A
ARHGAP1
ARGLU1
ARFRP1
ARFIP2
ARFGEF3
ARFGEF1
ARFGAP3
ARFGAP2
ARFGAP1
ARF6
ARF5
ARF4
ARF3
ARF1
AREL1
ARAP1
APTX
APRT
APPL2
APPL1
APOO
APOBR
APOBEC2
APLP1
APLN
APIP
APH1B
APH1A
APEX2
APEX1
APEH
APCDD1L
APBB1

APBA3
AP5Z1
AP5S1
AP5B1
AP4M1
AP4E1
AP4B1
AP3S1
AP3M2
AP3M1
AP3D1
AP3B2
AP3B1
AP2S1
AP2M1
AP2B1
AP2A2
AP2A1
AP1S3
AP1S2
AP1S1
AP1M2
AP1M1
AP1G2
AP1B1
AOPEP
ANXA9
ANXA5
ANXA2R
ANXA2
ANXA11
ANTKMT
ANP32E
ANP32B
ANP32A
ANO9
ANO7
ANO4
ANO10
ANLN
ANKZF1
ANKS6
ANKS3
ANKS1A

ANKRD9
ANKRD7
ANKRD65
ANKRD61
ANKRD54
ANKRD52
ANKRD49
ANKRD40
ANKRD39
ANKRD36C
ANKRD36B
ANKRD36
ANKRD34A
ANKRD27
ANKRD26
ANKRD23
ANKRD2
ANKRD18B
ANKRD16
ANKRD13D
ANKRD13B
ANKRD13A
ANKRD11
ANKRD10
ANKRD1
ANKMY2
ANKMY1
ANKLE2
ANKLE1
ANKK1
ANKHD1-EIF4EBP3
ANKHD1
ANKDD1B
ANKDD1A
ANKAR
ANGPTL5
ANGPT2
ANGEL2
ANGEL1
ANAPC7
ANAPC5
ANAPC4
ANAPC2
ANAPC16

ANAPC15
ANAPC13
ANAPC11
ANAPC1
AMZ2
AMPD3
AMPD2
AMMECR1L
AMMECR1
AMIGO3
AMH
AMDHD2
AMD1
AMBRA1
ALYREF
ALX4
ALX1
ALS2CL
ALPP
ALPK3
ALOXE3
ALOX12B
ALMS1
ALKBH6
ALKBH4
ALKBH2
ALKBH1
ALG9
ALG8
ALG6
ALG5
ALG3
ALG2
ALG1L2
ALG14
ALG13
ALG1
ALDOA
ALDH3B2
ALDH3B1
ALDH18A1
ALDH16A1
ALCAM
AKT1S1

AKR1C3
AKNAD1
AKIRIN2
AKIP1
AKAP8L
AKAP8
AKAP17A
AKAP10
AK9
AK6
AJM1
AIRE
AIP
AIMP2
AIMP1
AIFM3
AIFM2
AICDA
AHSA1
AHCY
AHCTF1
AGTRAP
AGRN
AGR2
AGPS
AGPAT4
AGPAT1
AGO2
AGK
AGFG1
AGER
AGBL5
AGBL3
AGAP9
AGAP6
AGAP4
AGAP3
AGAP2
AGAP1
AFTPH
AFP
AFG1L
AFAP1L1
AFAP1

AEN
AEBP2
ADSS2
ADSL
ADRM1
ADRB3
ADRA2C
ADRA1D
ADPRS
ADPRM
ADPGK
ADORA2B
ADORA1
ADO
ADNP2
ADNP
ADM5
ADM2
ADM
ADIPOR1
ADGRL1
ADGRG1
ADGRF4
ADGRF2
ADGRE5
ADGRD2
ADGRB2
ADGRB1
ADD3
ADD2
ADD1
ADCY6
ADCY2
ADCK5
ADCK2
ADCK1
ADAT3
ADAT2
ADAR
ADAP1
ADAMTS9
ADAMTS7
ADAMTS6
ADAMTS16

ADAMTS14
ADAM9
ADAM8
ADAM32
ADAM22
ADAM18
ADAM15
ADAM12
ADAM11
ADAM10
ADA
ACYP1
ACVRL1
ACVR2B
ACTR8
ACTR6
ACTR5
ACTR3B
ACTR3
ACTR1B
ACTR1A
ACTR10
ACTN4
ACTN3
ACTN1
ACTL8
ACTL6A
ACTL10
ACTG2
ACTG1
ACTBL2
ACTB
ACTA1
ACSS1
ACSL4
ACSL3
ACSBG2
ACSBG1
ACRV1
ACP6
ACP4
ACP3
ACP1
ACOXL

ACOT9
ACOT8
ACOT7
ACLY
ACIN1
ACER3
ACE
ACD
ACBD6
ACBD3
ACAP3
ACAN
ACAD9
ACACA
ABTB1
ABT1
ABRAXAS2
ABRAXAS1
ABRA
ABR
ABLIM2
ABL2
ABL1
ABITRAM
ABI2
ABI1
ABHD8
ABHD4
ABHD3
ABHD17C
ABHD17A
ABHD14A
ABHD12
ABHD11
ABCF3
ABCF2
ABCF1
ABCE1
ABCD1
ABCC8
ABCC5
ABCC4
ABCC10
ABCC1

ABCB9
ABCB8
ABCB6
ABCB5
ABCA7
ABCA4
ABCA3
ABCA12
AATK
AATF
AARSD1
AARS2
AARS1
AARD
AAR2
AANAT
AAMP
AAK1
AAGAB
AACS
AAAS
A4GNT
A4GALT

**SNHG17 negative-related genes in TCGA**	**Upregulated genes in RNA-seq data**	**Overlap**
ZNF385B	AC009086.2	MAT1A
ZMYND12	AL158066.1	COL25A1
ZG16	AC138696.1	HSD17B13
ZFP1	AC068234.1	DIO1
ZBTB16	AC006547.3	TDO2
XDH	ZFP91-CNTF	BMPER
XAF1	BORCS7-ASMT	ABCB4
WNK3	MAT1A	RASGEF1B
VWA8	BIVM-ERCC5	C4orf19
VIPR1	TBC1D3I	DUSP10
USH2A	COL25A1	FGB
UROC1	LINC02015	RDH5
UPP2	MMP23B	TSLP
UPB1	STRC	PDK4
UGT3A1	AC022028.2	RUNDC3B
UGT2B7	C1QTNF9	SERPING1
UGT2B4	PAQR9	NTN3

UGT2B17
UGT2B15
UGT2B10
UGT1A9
UGT1A4
UGT1A3
UGT1A1
UGP2
UBXN10
TUBE1
TTR
TTPA
TTC39B
TTC36
TTBK1
TSLP
TSKU
TRPM8
TRPC5
TRO
TRIM22
TREH
TRANK1
TPPP2
TOB1
TNXB
TMPRSS6
TMOD1
TMEM82
TMEM47
TMEM25
TMEM220
TMEM192
TMEM131L
TMEM100
TM6SF2
TLR4
TLR3
TLCD4
TIGD2
TIAM1
THRSP
TFR2
TF
TRIM6-TRIM34
AC005154.6
ABCA17P
AC009927.1
OLFML3
NPPC
DHH
LINGO1
PPFIA2
HULC
BTN1A1
AL139039.1
AL445483.1
CLEC20A
AP001107.5
TONSL-AS1
AL136309.2
GPR34
AC008982.1
AL713999.1
SLC4A10
ZNF98
RPL12P28
PDCD6IPP1
AP003419.4
AC009336.3
CLPSL2
LINC01885
SORCS1
AC068594.1
AC023824.7
AC100803.1
IL1R2
AP001062.3
CCDC197
SMTNL1
IGFBP7-AS1
AC025165.1
MOBP
TMEM150C
AC009097.1
DHRS9
AC005034.4
TRIM60P18
RSPO3
DLG2

TEX26	AC091868.1
TERB2	TEKT3
TEK	AC025287.1
TDO2	COL6A5
TCTEX1D1	CCDC54
TBX20	AC005052.1
TAT	AC100788.2
TAS2R60	NKAIN4
TAPT1	AC145423.2
TANGO6	CATSPERD
SYTL5	TGM7
SYT9	AC145285.4
SYT10	AC023644.1
SYNPO2	FBXL7
SYNE1	HSD17B13
SUOX	AL591475.1
SULT2A1	TRIM63
SULT1B1	AC084782.3
SUCNR1	LY96
SUCLG2	CCDC81
STX1B	AC012512.1
STEAP4	AC021549.1
STEAP3	MAGEB3
STBD1	PSMD4P1
STARD5	HSPB2
STAB2	HIST1H1T
ST8SIA6	HOXA11-AS1_2
ST6GAL1	AC005096.1
ST3GAL6	RPL12P42
SRL	SOX5
SRD5A2	CD80
SRD5A1	SLAMF7
SPTBN2	AC244102.1
SPRYD4	AL359551.1
SPP2	AL161719.1
SPDYC	AC113607.1
SPATA18	AC026464.2
SPACA7	LINC02404
SOWAHB	AL158196.1
SORL1	COX6CP2
SORD	AC113139.1
SORBS2	MIAT_exon5_3
SOCS2	GPR18
SMIM14	ZRANB2-AS1

SMARCA2	AP002812.1
SLITRK3	AL359853.2
SLCO2B1	CPLX2
SLCO1B1	ANKRD62
SLC9B2	SNORD16
SLC7A2	SIGLEC9
SLC6A13	AC010240.2
SLC6A12	AC093901.1
SLC6A1	AC009163.3
SLC51A	AC100788.1
SLC4A4	AC107982.1
SLC47A1	AC010997.3
SLC46A3	AC064801.1
SLC41A2	PRLR
SLC39A8	DIO1
SLC39A14	SNORA58B
SLC38A4	CCDC177
SLC38A3	SNORA1
SLC35D1	AP001527.1
SLC34A1	AC090617.6
SLC31A1	AC011489.1
SLC30A4	FYB1
SLC30A1	RAB28P5
SLC2A9	AL645465.1
SLC2A2	LINC02009
SLC2A12	TUBG1P
SLC28A1	AL450326.1
SLC27A5	RGS4
SLC27A2	CLDN14
SLC25A47	MYH16
SLC25A34	AC087190.4
SLC25A25	PLA1A
SLC25A20	MAGI2-AS3
SLC25A15	AC012254.2
SLC22A7	RHCG
SLC22A25	RN7SL832P
SLC22A10	AL354919.2
SLC22A1	IGDCC4
SLC1A2	MAGEB18
SLC1A1	HSPD1P11
SLC17A4	HMGA1P8
SLC17A2	AP002840.2
SLC17A1	NME9
SLC16A2	TNFRSF14

SLC13A5
SLC10A1
SHMT1
SHE
SHBG
SH3D19
SFXN1
SETD7
SERTM2
SERPING1
SERPINF2
SERPIND1
SERPINC1
SERPINA7
SERPINA6
SERPINA4
SERPINA11
SERPINA10
SEPSECS
SELP
SELENOP
SELENBP1
SELE
SEC24D
SEC14L3
SEC14L2
SDS
SDC1
SCP2
SC5D
SARDH
SAR1B
SALL1
SAA4
SAA2-SAA4
SAA2
SAA1
RUNDC3B
RTP3
RTL4
RSPO3
RORC
RORA
ROPN1B

VCX
PAEP
TP63
AC026471.6
AC020907.1
AC037198.3
PNMA6A
KLHL10
NRIP3
AC110015.1
RN7SL657P
FRMPD4
RHAG
AC004637.1
AC021028.1
PDXDC2P
LINC01293
AC121761.2
HTR3A
AC126603.1
ABHD14A-ACY1
CLVS1
DCLK1
HOXD4
SCN2A
AL049830.3
AC245096.1
HLA-J
PLEKHB1
AL049697.1
AC138028.6
DLGAP3
AC015818.2
AC106820.3
TNFRSF14-AS1
AC104452.1
CATSPERG
CELF5
AC120498.9
AGTR1
AC007731.5
SOX21-AS1
BHLHB9
TDO2

RNF152	RN7SL751P
RNF144B	MYO7A
RNF125	BMS1P4
RNASE4	PACSIN1
RMDN2	GAGE12H
RIPOR3	AL117329.1
RIDA	CRISPLD1
RHOB	LINC02274
RHCE	AC110597.3
RGS7BP	ZNF724
RGN	LINC02535
RFPL1	SCHIP1
RETREG1	AC122129.1
REPS2	ELFN2
REEP6	AC005225.5
RDH5	AL162258.2
RDH16	CCNT2-AS1
RD3L	AREG
RCAN1	HMCN1
RBP5	AC090970.3
RBP4	P2RX2
RASGEF1B	PPP1R36
RAPH1	C11orf96
RAPGEF4	ZNF624
RAPGEF2	MANEA-AS1
RANBP3L	AC002470.1
RAMP3	AC245060.5
QDPR	DDO
PZP	CYP1B1
PXMP2	ZNF658B
PTPRB	CDRT4
PTGR1	SYCE2
PSD3	AL353743.4
PRRG4	CDK14
PROZ	RASGRF2
PROS1	PRKG1
PRKAG2	MAPK8IP2
PRG4	AC100803.2
PRAMEF33	AC096887.1
PRAMEF10	DUX4L27
PPP4R4	RBBP8NL
PPP1R3B	AL591684.2
PPL	AJ271736.1
PPARGC1A	LINC02029

PPARA	LRRC37A11P
PON3	VCX3A
PON1	CD8A
PNPO	SOBP
PNPLA7	AL512408.1
PLSCR4	RELN
PLPP3	ZNF100
PLG	BMPER
PLCXD3	POU5F1B
PLCL2	CDH5
PIPOX	IL20
PINK1	EGF
PIK3R1	ACP5
PIK3C2G	AC024257.3
PIGV	LINC00880
PIGR	LINC02475
PI4K2B	GPR3
PHYHD1	HIST1H2BJ
PHYH	AL138976.2
PGRMC1	DLEU2_1
PGM1	AC010503.4
PGLYRP2	AC093495.1
PFKFB1	SP9
PEX11G	PIWIL2
PECR	CCDC150P1
PEBP4	ZNF430
PDZRN4	AL137002.2
PDLIM5	GAGE2A
PDK4	AL117209.1
PDE7B	TARID
PDE3B	TEX14
PDE2A	AC008406.3
PCYOX1	LHX9
PCSK6	PTHLH
PCK2	AC112220.4
PCK1	RNF157
PCCB	C1QTNF2
PC	P4HA3
PBLD	PTN
PAPSS2	TMEM200C
PAPPA2	KCNMB2-AS1
PANK1	TPM4
PALM2AKAP2	NAMPTP1
PAH	S1PR1

P2RX3	ABCB4
OTC	AC242426.3
ORM2	AC092279.2
ORM1	MXD1
OMD	ATF3
OIT3	PELI2
OGDHL	AC009163.7
OAF	PLAU
NXF3	AGT
NUGGC	AC087623.4
NUDT7	PGM2L1
NUDT6	AC008764.1
NUDT12	TRPM2-AS
NTN3	LINC01535
NTF3	C3orf52
NR3C2	AC016831.1
NR1I3	PTGS2
NR1I2	TNC
NPY1R	SLC6A14
NOL4	DAND5
NNT	PCDH15
NLRP6	ARHGAP6
NLRP14	AC068768.1
NFIA	AC008894.3
NDST3	RASGEF1B
NDRG2	KLHL35
ND6	PLPPR4
ND5	GANAB
ND4L	PLAT
NAT2	DLEU2_6
NAT1	ANPEP
NAAA	NFIX
N4BP2L1	NCAM1
MYRIP	ADGRF4
MYO1B	AC022400.6
MYO16	PACERR
MYLK	AC098614.1
MTUS2	LINC00882
MTTP	COCH
MTHFS	AC004477.1
MTHFD1	LINC01556
MTARC2	DMC1
MT2A	FNDC4
MT1X	CADPS2

MT1M	PRTFDC1
MT1G	NR4A3
MT1E	ZNF253
MSRA	VGF
MROH2A	AC126544.1
MRO	ZNF658
MRC1	C4orf19
MPDZ	NECAB1
MOGAT2	LINC01126
MOGAT1	ZNF616
MMUT	SPOCD1
MME	ZNF93
MMAA	ZBTB43
MLYCD	KCNS3
MLIP	CXCL2
MIP	NEUROG2
MICU3	EMP1
MGST1	TMEM33
MFSD2A	AP001350.2
MFAP3L	GEM
METTL7A	DUSP10
MEP1B	C1orf140
MEGF10	USP51
MCC	G3BP2
MBL2	AC017100.1
MAT1A	STX12
MASP2	MESP2
MASP1	SEPT4
MAP3K5	AC092720.1
MAOB	ZNF184
MAN1C1	AL162377.1
LYVE1	VASH2
LYPD2	ZNF14
LRRC3	CRYAB
LRRC2	CXCL8
LRCOL1	VDAC1P8
LPIN2	ZNF714
LPA	TAPT1-AS1
LONP2	AL162595.1
LOC107987479; CYP2D6; LOC107987478	MEMO1P1
LOC101929773	SLC25A21
LOC100132813	SYT11
LIPG	MRAS

LINGO4	OLFML2B
LILRB5	RPL18P13
LGI1	LGALS8-AS1
LEPR	AC004803.1
LECT2	LYPD6
LEAP2	RNF185
LDHD	FGB
LDB2	AL513523.1
LCAT	RYR2
KNG1	ZNF736
KMO	HIST1H2BD
KLRF1	AC087499.1
KLKB1	SGIP1
KLHL2	IGFBP1
KLF9	ZFAND4
KLF15	TOX3
KIAA2012	COL24A1
KHK	AL451074.2
KDR	TM4SF1
KDM8	SELPLG
KCNN2	LINC01116
KCNK17	ZNF91
KCNJ8	EXOC3-AS1
KCND3	ZNF204P
KCNB1	ZNF154
KBTBD11	GNPDA1
KAT2B	RDH5
IYD	YEATS2-AS1
IVD	AL135838.1
ITIH3	RNF138
ITIH1	TSLP
ISOC1	NES
IQSEC3	DPY19L2P2
IQGAP2	INHBA
INS-IGF2	CRCP
INSIG1	CREB5
INMT	SLC2A3
INHBC	LMTK3
IL33	PRSS22
IL27	EOMES
IL1RL1	SESN2
IL1RAPL2	ERV3-1
IL18R1	ASB1
IGFBP4	ZNF25

IGFALS	AC092143.3
IGF1	AC096733.2
IFIT1	ZNF426
IDO2	C19orf57
IDNK	PDK4
HTR2A	MAGEA2B
HSD3B2	NKX3-1
HSD3B1	EFHC2
HSD17B6	PAG1
HSD17B4	TRIB3
HSD17B13	NFATC2
HSD11B1	CXXC4
HRG	ZNF680
HPX	RUNDC3B
HPR	RRAGD
HPD	SPINK1
HP	AC074091.1
HORMAD2	GOLT1A
HOMER2	AC080080.1
HMGN5	LINC00957
HMGCS2	NACAD
HMGCLL1	AC133065.4
HMGCL	IL6R
HLF	ZNF572
HJV	LIMK1
HIBADH	LINC00518
HHIP	LPP-AS2
HGFAC	ZNF766
HGD	ITGA7
HERC5	B4GALT1-AS1
HERC3	RRM2
HEPACAM	KLF6
HDC	ZNF554
HAO2	THBS1
HAO1	AC009779.3
HAMP	IGFN1
HAGH	ZDHHC16
HADH	B4GALT4-AS1
HAAO	SERPING1
GYS2	RCN3
GSTZ1	ZMAT1
GSTA2	RGS2
GSTA1	AL606834.2
GRIN2B	AC073072.1

GRIA3	CTSH
GRHPR	ZNF726
GREM2	LONRF1
GRAMD1C	THRB
GPT2	SAMD12
GPT	TXNIP
GPR88	MIR503HG
GPR182	GALNT13
GPR146	TBC1D9
GPM6A	ZNF845
GPLD1	FGD4
GPHN	AC005332.7
GPD1	SLC9A3
GOT2	TRIML2
GOLGA6B	GEN1
GOLGA6A	ZNF429
GNMT; CNPY3-GNMT	AC015660.1
GNE	RASAL2
GNAO1	MIATNB
GNA14	ZNF528-AS1
GMNC	C3orf67
GLYCTK	PERP
GLYATL3	AC006252.1
GLYATL1	TMX3
GLYAT	ADAMTS3
GLS2	BAALC-AS1
GLOD5	MOXD1
GJB2	SCARB2
GIPC2	GNA11
GHR	AP000695.2
GFRA1	CHST14
GDNF	SNX10
GDF2	AL355001.2
GDA	RGS19
GCKR	NTN3
GCK	PARM1
GCH1	C1QTNF6
GCGR	AC093010.3
GCDH	FAM173B
GC	NLGN4X
GBP7	TTC33
GBA3	AC073508.2
GATM	LINGO2
GASK1A	IKZF2

GALNT15	LINC02228
GADD45A	ZNF134
GABARAPL1	AL358472.4
G6PC	HSD17B14
FYB2	AL158151.3
FXYD1	DGAT2
FUCA1	GPR173
FTCDNL1	EPDR1
FTCD	HIVEP3
FRRS1	C18orf54
FRMD7	CTSL
FREM2	HIST1H4H
FOSB	RDM1
FOLH1B	MEX3D
FOLH1	LBX2-AS1
FNDC5	FICD
FMO5	AC092645.1
FMO4	SNAI2
FMO3	FOXP4
FMO2	ZNF860
FITM1	GALNT3
FGGY	CLUHP3
FGG	SERPINB4
FGB	HABP4
FGA	TCF19
FETUB	ATP8B2
FCN3	ZNF211
FCN2	AC096887.2
FCAMR	AL117336.3
FBXO8	DENND5B-AS1
FBP1	GUCY1A2
FAXDC2	ZNF615
FAT4	WDFY3-AS2
FAS	CRISPLD2
FAM180A	TVP23A
FAM163B	ADAMTS5
FAM149A	HIST1H3D
FAM13A	HIST2H2BE
FAH	DLEU2
F9	SYT1
F8	UBE2D1
F7	CXCL3
F5	ZNF165
F2	ADTRP

F13B	ZFP37
F12	FLT3LG
F11	SERPINB3
EXPH5	PSMB8-AS1
ETNPPL	KALRN
ETNK2	AL133346.1
ETFRF1	LCORL
ETFDH	C14orf28
ETFBKMT	AC037198.1
ESR1	PLCL1
ESPN	KCNH1
ERICH3	CTAGE7P
EPHX2	RSPO3
EPHX1	GPR183
ENTPD5	BIRC7
ENPP7	MYBL1
ENPEP	VIL1
ELFN1	MED11
EHHADH	AL513550.1
EDNRB	DLG2
EDEM1	AL606489.1
ECM2	ZNF17
ECHS1	DDIT3
ECHDC2	KRBA2
DUSP10	ZNF675
DUSP1	SLC7A8
DSG1	AL121944.1
DRD1	AC241585.1
DPYS	ZNF26
DPT	ZNF45
DPF3	SPOPL
DNASE1L3	FUT4
DNAJC25	FNDC10
DNAH6	IL6
DMGDH	ATF1
DMD	BOLA3-AS1
DLGAP2	ZMYND10
DLG2	BHLHE41
DIRAS3	LATS2
DIO1	MYO15B
DHTKD1	B4GALT1
DHRS4L1	GPR143
DHRS1	AC092295.2
DHODH	GRIN1

DEPDC7
DDX60L
DDX60
DCXR
DCAF11
DBT
DBH
DAO
CYTB
CYP8B1
CYP7B1
CYP7A1
CYP4X1
CYP4V2
CYP4F3
CYP4F2
CYP4F12
CYP4F11
CYP4A22
CYP4A11
CYP3A43
CYP3A4
CYP39A1
CYP2U1
CYP2J2
CYP2C9
CYP2C8
CYP2C19
CYP2C18
CYP2B6
CYP2A7
CYP2A6
CYP2A13
CYP27A1
CYP26A1
CYP1A2
CYP1A1
CYB5A
CXorf66
CWH43
CUX2
CTSO
CTNNA3
CTH
PRR16
UBE2Q2P1
PDE9A
CREBRF
GPAT3
ZNF227
ZNF484
POMK
ZNF257
ZNF84
ETAA1

CTBS
CRYZL2P-SEC16B; SEC16B
CRYL1
CRYBG1
CRYAA
CRHBP
CPT2
CPS1
CPN2
CPED1
CPEB3
CPB2
CP
COLEC10
COL25A1
COL18A1
COBLL1
CNTN3
CNDP1
CMBL
CLYBL
CLTRN
CLEC4M
CLEC4G
CLEC3B
CLEC1B
CIDEB
CHRNA4
CHRM2
CHAD
CGNL1
CFI
CFHR5
CFHR4
CFHR3
CFHR2
CFHR1
CFH
CFB
CFAP57
CETP
CES5A
CES4A
CES3

CES2
CES1
CDO1
CDHR5
CDH23
CDC37L1
CDC14B
CDA
CD5L
CD302
CD300LG
CD1D
CD14
CCL23
CCL16
CCL14
CCDC38
CCDC196
CCDC158
CCBE1
CBR4
CAT
CAMK2B
CA5A
CA2
C9
C8B
C8A
C7
C6
C4orf19
C4BPA
C3orf85
C3
C1S
C1RL
C1R
C14orf180
C11orf54
BTD
BMX
BMPER
BMP10
BMERB1

BHMT2
BHMT
BDH1
BCO2
BCKDHB
BCHE
BBOX1
BAAT
AZGP1
AVPR1A
AUTS2
ATP11C
ATOH8
ASXL3
ASS1
ASPDH
ASPA
ARSF
ARSD
ARRDC4
ARPP21
ARID3C
ARHGAP42
ARHGAP20
ARG1
AR
AQP9
APOL6
APOH
APOF
APOC4
APOC3
APOB
APOA5
APCS
APBA1
AOX1
ANXA10
ANO3
ANO1
ANKS4B
ANGPTL6
ANGPTL3
ANGPTL1

ANG
AMDHD1
AMBP
ALPL
ALDOB
ALDH9A1
ALDH8A1
ALDH7A1
ALDH6A1
ALDH5A1
ALDH2
ALDH1L1
ALDH1A1
ALB
ALAD
AKR7A3
AKR1D1
AKAP6
AKAP3
AHSG
AGXT2
AGXT
AGMO
AGMAT
AGL
AFM
ADRB2
ADRA2B
ADRA1B
ADRA1A
ADI1
ADHFE1
ADH7
ADH6
ADH4
ADH1C
ADH1B
ADH1A
ADGRG7
ADGRA3
ADCY1
ADAMTSL3
ACY3
ACVR1C

ACSM5
ACSM3
ACSM2B
ACSM2A
ACSL5
ACSL1
ACOX2
ACOX1
ACOT12
ACO1
ACMSD
ACKR2
ACE2
ACBD4
ACAT1
ACADSB
ACADS
ACADM
ACADL
ACAD11
ACACB
ACAA2
ACAA1
ABHD6
ABHD2
ABCG8
ABCG5
ABCG2
ABCC9
ABCC6
ABCC11
ABCB4
ABCB11
ABCA9
ABCA8
ABCA6
ABAT
AASS
AADAT
AADAC
A1CF
A1BG
